# Supplementary material for: Where is your field going? A machine learning approach to study the relative motion of the domains of physics
Source: PLoS One. 2020 Jun 18;15(6):e0233997. doi: 10.1371/journal.pone.0233997 (PMC7302634; doi:10.1371/journal.pone.0233997)
Supplement: S1 File — (PDF) [file pone.0233997.s033.pdf]

00. GENERAL

01. Communication, education, history, and philosophy

01.10.−m Announcements, news, and organizational activities

01.10.Cr Announcements, news, and awards
01.10.Fv Conferences, lectures, and institutes
01.10.Hx Physics organizational activities

01.20.+x Communication forms and techniques (written, oral, electronic, etc.)

01.30.−y Physics literature and publications

01.30.Bb Publications of lectures (advanced institutes, summer schools, etc.)
01.30.Cc Conference proceedings
01.30.Ee Monographs and collections
01.30.Kj Handbooks, dictionaries, tables, and data compilations
01.30.Mm Textbooks for graduates and researchers
01.30.Pp Textbooks for undergraduates
01.30.Rr Surveys and tutorial papers; resource letters
01.30.Tt Bibliographies
01.30.Vv Book reviews
01.30.Xx Publications in electronic media *(for the topic of electronic publishing, see 01.20.+x)*

01.40.−d Education

01.40.Di Course design and evaluation
01.40.Ej Science in elementary and secondary school
01.40.Fk Physics education research (cognition, problem solving, etc.)
01.40.Gm Curricula; teaching methods, strategies, theory of testing and evaluation
01.40.Jp Teacher training

01.50.−i Educational aids

01.50.Fr Audio and visual aids, films
01.50.Ht Instructional computer use
01.50.Kw Techniques of testing
01.50.Lc Laboratory computer use *(see also 01.50.Pa)*
01.50.My Demonstration experiments and apparatus
01.50.Pa Laboratory experiments and apparatus *(see also 01.50.Lc)*
01.50.Qb Laboratory course design, organization, and evaluation
01.50.Wg Physics of toys

01.52.+r National and international laboratory facilities

01.55.+b General physics

01.60.+q Biographies, tributes, personal notes, and obituaries

01.65.+g History of science

01.70.+w Philosophy of science

01.75.+m Science and society *(for science and government, see 01.78.+p)*

01.78.+p Science and government (funding, politics, etc.)

01.80.+b Physics of sports

01.90.+g Other topics of general interest (restricted to new topics in section 01)

02. Mathematical methods in physics

02.10.−v Logic, set theory, and algebra

02.10.Ab Logic and set theory
02.10.De Algebraic structures and number theory
02.10.Hh Rings and algebras
02.10.Kn Knot theory
02.10.Ox Combinatorics; graph theory
02.10.Ud Linear algebra
02.10.Xm Multilinear algebra
02.10.Yn Matrix theory

02.20.−a Group theory *(for algebraic methods in quantum mechanics, see 03.65.Fd; for symmetries in elementary particle physics, see 11.30.−j)*

02.20.Bb General structures of groups
02.20.Hj Classical groups
02.20.Qs General properties, structure, and representation of Lie groups
02.20.Rt Discrete subgroups of Lie groups
02.20.Sv Lie algebras of Lie groups
02.20.Tw Infinite-dimensional Lie groups
02.20.Uw Quantum groups

02.30.−f Function theory, analysis

02.30.Cj Measure and integration
02.30.Em Potential theory
02.30.Fn Several complex variables and analytic spaces
02.30.Gp Special functions
02.30.Hq Ordinary differential equations
02.30.Ik Integrable systems
02.30.Jr Partial differential equations
02.30.Ks Delay and functional equations
02.30.Lt Sequences, series, and summability
02.30.Mv Approximations and expansions
02.30.Nw Fourier analysis
02.30.Oz Bifurcation theory *(see also 47.20.Ky in fluid dynamics)*
02.30.Px Abstract harmonic analysis
02.30.Rz Integral equations
02.30.Sa Functional analysis
02.30.Tb Operator theory
02.30.Uu Integral transforms
02.30.Vv Operational calculus
02.30.Xx Calculus of variations
02.30.Yy Control theory
02.30.Zz Inverse problems

02.40.−k Geometry, differential geometry, and topology *(see also section 04 Relativity and gravitation)*

02.40.Dr Euclidean and projective geometries
02.40.Ft Convex sets and geometric inequalities
02.40.Gh Noncommutative geometry
02.40.Hw Classical differential geometry
02.40.Ky Riemannian geometries
02.40.Ma Global differential geometry
02.40.Pc General topology
02.40.Re Algebraic topology
02.40.Sf Manifolds and cell complexes
02.40.Tt Complex manifolds
02.40.Vh Global analysis and analysis on manifolds
02.40.Xx Singularity theory *(see also 05.45.−a in statistical physics, thermodynamics, and nonlinear dynamical systems)*
02.40.Yy Geometric mechanics *(see also 45.20.Jj in formalisms in classical mechanics)*

02.50.−r Probability theory, stochastic processes, and statistics *(see also section 05 Statistical physics, thermodynamics, and nonlinear dynamical systems)*

02.50.Cw Probability theory
02.50.Ey Stochastic processes
02.50.Fz Stochastic analysis
02.50.Ga Markov processes
02.50.Le Decision theory and game theory
02.50.Ng Distribution theory and Monte Carlo studies
02.50.Sk Multivariate analysis
02.50.Tt Inference methods

02.60.−x Numerical approximation and analysis

02.60.Cb Numerical simulation; solution of equations
02.60.Dc Numerical linear algebra
02.60.Ed Interpolation; curve fitting
02.60.Gf Algorithms for functional approximation
02.60.Jh Numerical differentiation and integration
02.60.Lj Ordinary and partial differential equations; boundary value problems
02.60.Nm Integral and integrodifferential equations
02.60.Pn Numerical optimization

02.70.−c **Computational techniques** *(for quantum computation, see 03.67.Lx)*
02.70.Bf Finite-difference methods
02.70.Dh Finite-element and Galerkin methods
02.70.Hm Spectral methods
02.70.Jn Collocation methods
02.70.Ns Molecular dynamics and particle methods
02.70.Pt Boundary-integral methods
02.70.Rr General statistical methods
02.70.Ss Quantum Monte Carlo methods
02.70.Tt Justifications or modifications of Monte Carlo methods
02.70.Uu Applications of Monte Carlo methods *(see also 02.50.Ng in probability theory, stochastic processes, and statistics, and 05.10.Ln in statistical physics)*
02.70.Wz Symbolic computation (computer algebra)

02.90.+p **Other topics in mathematical methods in physics (restricted to new topics in section 02)**

03. Quantum mechanics, field theories, and special relativity *(see also section 11 General theory of fields and particles)*

03.30.+p **Special relativity**

03.50.−z **Classical field theories**
03.50.De Classical electromagnetism, Maxwell equations *(for applied classical electromagnetism, see 41.20.−q)*
03.50.Kk Other special classical field theories

03.65.−w **Quantum mechanics** *(see also 03.67.−a Quantum information; 05.30.−d Quantum statistical mechanics)*
03.65.Ca Formalism
03.65.Db Functional analytical methods
03.65.Fd Algebraic methods *(see also 02.20.−a Group theory)*
03.65.Ge Solutions of wave equations: bound states
03.65.Nk Scattering theory
03.65.Pm Relativistic wave equations
03.65.Sq Semiclassical theories and applications
03.65.Ta Foundations of quantum mechanics; measurement theory *(for optical tests of quantum theory, see 42.50.Xa)*
03.65.Ud Entanglement and quantum nonlocality (e.g. EPR paradox, Bell's inequalities, GHZ states, etc.) *(for entanglement production in quantum information, see 03.67.Mn; for entanglement in Bose-Einstein condensates, see 03.75.Gg)*
03.65.Vf Phases: geometric; dynamic or topological
03.65.Wj State reconstruction, quantum tomography
03.65.Xp Tunneling, traversal time, quantum Zeno dynamics
03.65.Yz Decoherence; open systems; quantum statistical methods *(see also 03.67.Pp in quantum information; for decoherence in Bose-Einstein condensates, see 03.75.Gg)*

03.67.−a **Quantum information**
03.67.Dd Quantum cryptography
03.67.Hk Quantum communication
03.67.Lx Quantum computation
03.67.Mn Entanglement production, characterization and manipulation *(see also 03.65.Ud Entanglement and quantum nonlocality; for entanglement in Bose-Einstein condensates, see 03.75.Gg)*
03.67.Pp Quantum error correction and other methods for protection against decoherence *(see also 03.65.Yz Decoherence; open systems; quantum statistical methods; for decoherence in Bose-Einstein condensates, see 03.75.Gg)*

03.70.+k **Theory of quantized fields** *(see also 11.10.−z Field theory)*

03.75.−b **Matter waves** *(for atom interferometry techniques, see 39.20.+q—in atomic and molecular physics)*
03.75.Be Atom and neutron optics
03.75.Dg Atom and neutron interferometry
03.75.Gg Entanglement and decoherence in Bose-Einstein condensates
03.75.Hh Static properties of condensates; thermodynamical, statistical and structural properties.
03.75.Kk Dynamic properties of condensates; collective and hydrodynamic excitations, superfluid flow
03.75.Lm Tunneling, Josephson effect, Bose-Einstein condensates in periodic potentials, solitons, vortices and topological excitations
03.75.Mn Multicomponent condensates; spinor condensates
03.75.Nt Other Bose-Einstein condensation phenomena
03.75.Pp Atom lasers
03.75.Ss Degenerate Fermi gases

04. General relativity and gravitation *(see also 95.30.Sf in astronomy)*

. . . . *Special relativity, see 03.30.+p*

04.20.−q **Classical general relativity** *(see also 02.40.−k Geometry, differential geometry, and topology)*
04.20.Cv Fundamental problems and general formalism
04.20.Dw Singularities and cosmic censorship
04.20.Ex Initial value problem, existence and uniqueness of solutions
04.20.Fy Canonical formalism, Lagrangians, and variational principles
04.20.Gz Spacetime topology, causal structure, spinor structure
04.20.Ha Asymptotic structure
04.20.Jb Exact solutions

04.25.−g **Approximation methods; equations of motion**
04.25.Dm Numerical relativity
04.25.Nx Post-Newtonian approximation; perturbation theory; related approximations

04.30.−w **Gravitational waves: theory**
04.30.Db Wave generation and sources
04.30.Nk Wave propagation and interactions

04.40.−b **Self-gravitating systems; continuous media and classical fields in curved spacetime**
04.40.Dg Relativistic stars: structure, stability, and oscillations *(see also 97.60.−s Late stages of stellar evolution)*
04.40.Nr Einstein−Maxwell spacetimes, spacetimes with fluids, radiation or classical fields

04.50.+h **Gravity in more than four dimensions, Kaluza−Klein theory, unified field theories; alternative theories of gravity** *(see also 11.25.Mj Compactification and four-dimensional models)*

04.60.−m **Quantum gravity**
04.60.Ds Canonical quantization
04.60.Gw Covariant and sum-over-histories quantization
04.60.Kz Lower dimensional models; minisuperspace models
04.60.Nc Lattice and discrete methods
04.60.Pp Loop quantum gravity, quantum geometry, spin foams

04.62.+v **Quantum field theory in curved spacetime**

04.65.+e **Supergravity** *(see also 12.60.Jv Supersymmetric models)*

04.70.−s **Physics of black holes** *(see also 97.60.Lf—in astronomy)*
04.70.Bw Classical black holes
04.70.Dy Quantum aspects of black holes, evaporation, thermodynamics

04.80.−y **Experimental studies of gravity**
04.80.Cc Experimental tests of gravitational theories
04.80.Nn Gravitational wave detectors and experiments *(see also 95.55.Ym—in astronomy)*

04.90.+e Other topics in general relativity and gravitation (restricted to new topics in section 04)

05. Statistical physics, thermodynamics, and nonlinear dynamical systems *(see also 02.50.−r Probability theory, stochastic processes, and statistics)*

05.10.−a Computational methods in statistical physics and nonlinear dynamics *(see also 02.70.−c in mathematical methods in physics)*

05.10.Cc Renormalization group methods

05.10.Gg Stochastic analysis methods (Fokker–Planck, Langevin, etc.)

05.10.Ln Monte Carlo methods *(see also 02.70.Tt, Uu in mathematical methods in physics; for Monte Carlo methods in plasma simulation, see 52.65.Pp)*

05.20.−y Classical statistical mechanics

05.20.Dd Kinetic theory *(see also 51.10.+y Kinetic and transport theory of gases)*

05.20.Gg Classical ensemble theory

05.20.Jj Statistical mechanics of classical fluids *(see also 47.10.+g General theory in fluid dynamics)*

05.30.−d Quantum statistical mechanics

05.30.Ch Quantum ensemble theory

05.30.Fk Fermion systems and electron gas *(see also 71.10.−w Theories and models of many-electron systems)*

05.30.Jp Boson systems *(for static and dynamic properties of Bose-Einstein condensates, see 03.75.Hh and 03.75.Kk)*

05.30.Pr Fractional statistics systems (anyons, etc.)

05.40.−a Fluctuation phenomena, random processes, noise, and Brownian motion *(for fluctuations in superconductivity, see 74.40.+k; for statistical theory and fluctuations in nuclear reactions, see 24.60.−k; for fluctuations in plasma, see 52.25.Gj)*

05.40.Ca Noise

05.40.Fb Random walks and Levy flights

05.40.Jc Brownian motion

05.45.−a Nonlinear dynamics and nonlinear dynamical systems *(see also section 45 Classical mechanics of discrete systems)*

05.45.Ac Low-dimensional chaos

05.45.Df Fractals *(see also 47.53.+n Fractals in fluid dynamics)*

05.45.Gg Control of chaos, applications of chaos

05.45.Jn High-dimensional chaos

05.45.Mt Quantum chaos; semiclassical methods

05.45.Pq Numerical simulations of chaotic systems

05.45.Ra Coupled map lattices

05.45.Tp Time series analysis

05.45.Vx Communication using chaos

05.45.Xt Synchronization; coupled oscillators

05.45.Yv Solitons *(see 52.35.Sb for solitons in plasma; for solitons in acoustics, see 43.25.Rq—in acoustics appendix; see 42.50.Md, 42.65.Tg, 42.81.Dp for solitons in optics; see also 03.75.Lm Tunneling, Josephson effect, Bose-Einstein condensates in periodic potentials, solitons, vortices and topological excitations)*

05.50.+q Lattice theory and statistics (Ising, Potts, etc.) *(see also 64.60.Cn Order–disorder transformations and statistical mechanics of model systems and 75.10.Hk Classical spin models)*

05.60.−k Transport processes

05.60.Cd Classical transport

05.60.Gg Quantum transport

05.65.+b Self-organized systems *(see also 45.70.−n in classical mechanics of discrete systems)*

05.70.−a Thermodynamics *(see also section 64 Equations of state, phase equilibria, and phase transitions, and section 65 Thermal properties of condensed matter; for chemical thermodynamics, see 82.60.−s; for thermodynamics of plasmas, see 52.25.Kn)*

. . . . *Thermodynamics of nanoparticles, see 82.60.Qr*

05.70.Ce Thermodynamic functions and equations of state *(see also 51.30.+i Thermodynamic properties, equations of state in physics of gases)*

05.70.Fh Phase transitions: general studies

05.70.Jk Critical point phenomena

05.70.Ln Nonequilibrium and irreversible thermodynamics *(see also 82.40.Bj Oscillations, chaos, and bifurcations in physical chemistry and chemical physics)*

05.70.Np Interface and surface thermodynamics *(see also 68.35.Md Surface thermodynamics, surface energies in surfaces and interfaces)*

05.90.+m Other topics in statistical physics, thermodynamics, and nonlinear dynamical systems (restricted to new topics in section 05)

06. Metrology, measurements, and laboratory procedures *(for laser applications in metrology, see 42.62.Eh)*

06.20.−f Metrology

06.20.Dk Measurement and error theory

06.20.Fn Units and standards

06.20.Jr Determination of fundamental constants

06.30.−k Measurements common to several branches of physics and astronomy

06.30.Bp Spatial dimensions (e.g., position, lengths, volume, angles, and displacements)

06.30.Dr Mass and density

06.30.Ft Time and frequency

06.30.Gv Velocity, acceleration, and rotation

06.60.−c Laboratory procedures

06.60.Ei Sample preparation (including design of sample holders)

06.60.Jn High-speed techniques (microsecond to femtosecond)

06.60.Mr Testing and inspecting procedures

06.60.Sx Positioning and alignment; manipulating, remote handling

06.60.Vz Workshop procedures (welding, machining, lubrication, bearings, etc.)

06.60.Wa Laboratory safety procedures

. . . . *National and international laboratory facilities, see 01.52.+r*

06.90.+v Other topics in metrology, measurements, and laboratory procedures (restricted to new topics in section 06)

07. Instruments, apparatus, and components common to several branches of physics and astronomy *(see also each subdiscipline for specialized instrumentation and techniques)*

07.05.−t Computers in experimental physics

. . . . *Computers in physics education, see 01.50.Ht and 01.50.Lc*

. . . . *Computational techniques, see 02.70.−c—in mathematical methods in physics*

. . . . *Quantum computation, see 03.67.Lx in quantum mechanics*

07.05.Bx Computer systems: hardware, operating systems, computer languages, and utilities

07.05.Dz Control systems

07.05.Fb Design of experiments

07.05.Hd Data acquisition: hardware and software

07.05.Kf Data analysis: algorithms and implementation; data management

07.05.Mh Neural networks, fuzzy logic, artificial intelligence

07.05.Pj Image processing *(see also 42.30.Va in optics; 87.57.−s Medical imaging: general in biological and medical physics)*

07.05.Rm Data presentation and visualization: algorithms and implementation

07.05.Tp Computer modeling and simulation

07.05.Wr Computer interfaces

07.07.−a General equipment

07.07.Df Sensors (chemical, optical, electrical, movement, gas, etc.); remote sensing

07.07.Hj Display and recording equipment, oscilloscopes, TV cameras, etc.

07.07.Mp Transducers

07.07.Tw Servo and control equipment; robots

07.07.Vx Hygrometers

07.10.−h Mechanical instruments and equipment

07.10.Cm Micromechanical devices and systems *(for micro- and nano-electromechanical systems (MEMS/NEMS), see 85.85.+j in electronic and magnetic devices)*

07.10.Fq Vibration isolation

07.10.Lw Balance systems, tensile machines, etc.

07.10.Pz Instruments for strain, force, and torque

07.20.−n Thermal instruments and apparatus

07.20.Dt Thermometers

07.20.Fw Calorimeters *(for calorimeters as radiation detectors, see 29.40.Vj)*

07.20.Hy Furnaces; heaters

07.20.Ka High-temperature instrumentation; pyrometers

07.20.Mc Cryogenics; refrigerators, low-temperature equipment

07.20.Pe Heat engines; heat pumps; heat pipes

07.30.−t Vacuum apparatus

07.30.Bx Degasification, residual gas

07.30.Cy Vacuum pumps

07.30.Dz Vacuum gauges

07.30.Hd Vacuum testing methods; leak detectors

07.30.Kf Vacuum chambers, auxiliary apparatus, and materials

07.35.+k High-pressure apparatus; shock tubes; diamond anvil cells

07.50.−e Electrical and electronic instruments and components

07.50.Ek Circuits and circuit components *(see also 84.30.−r Electronic circuits and 84.32.−y Passive circuit components)*

07.50.Hp Electrical noise and shielding equipment

07.50.Ls Electrometers

07.50.Qx Signal processing electronics *(see also 84.40.Ua in radiowave and microwave technology)*

07.55.−w Magnetic instruments and components

07.55.Db Generation of magnetic fields; magnets *(for superconducting magnets, see 84.71.Ba)*

07.55.Ge Magnetometers for magnetic field measurements

07.55.Jg Magnetometers for susceptibility, magnetic moment, and magnetization measurements

07.55.Nk Magnetic shielding in instruments

07.57.−c Infrared, submillimeter wave, microwave and radiowave instruments and equipment *(for infrared and radio telescopes, see 95.55.Cs, 95.55.Fw, and 95.55.Jz in astronomy)*

07.57.Hm Infrared, submillimeter wave, microwave, and radiowave sources

07.57.Kp Bolometers; infrared, submillimeter wave, microwave, and radiowave receivers and detectors *(see also 85.60.Gz Photodetectors in electronic and magnetic devices, and 95.55.Rg Photoconductors and bolometers in astronomy)*

07.57.Pt Submillimeter wave, microwave and radiowave spectrometers; magnetic resonance spectrometers, auxiliary equipment, and techniques

07.57.Ty Infrared spectrometers, auxiliary equipment, and techniques

07.60.−j Optical instruments and equipment

. . . . *Optical sources, see 42.72.−g*

. . . . *Optical elements, devices, and systems 42.79.−e*

. . . . *Optoelectronic devices 85.60.−q*

. . . . *Optical telescopes, see 95.55.Cs*

07.60.Dq Photometers, radiometers, and colorimeters

07.60.Fs Polarimeters and ellipsometers

07.60.Hv Refractometers and reflectometers

07.60.Ly Interferometers

07.60.Pb Conventional optical microscopes *(for near-field scanning optical microscopes, see 07.79.Fc; for x-ray microscopes, see 07.85.Tt)*

07.60.Rd Visible and ultraviolet spectrometers

07.60.Vg Fiber-optic instruments *(see also 42.81.−i Fiber optics—in optics)*

07.64.+z Acoustic instruments and equipment *(see also 43.58.+z—in acoustics)*

07.68.+m Photography, photographic instruments; xerography

07.75.+h Mass spectrometers *(see also 82.80.Ms, 82.80.Nj, and 82.80.Rt in physical chemistry and chemical physics)*

07.77.−n Atomic, molecular, and charged-particle sources and detectors

07.77.Gx Atomic and molecular beam sources and detectors *(see also 39.10.+j in atomic and molecular physics)*

07.77.Ka Charged-particle beam sources and detectors *(see also 29.40.−n in nuclear physics)*

07.78.+s Electron, positron, and ion microscopes; electron diffractometers

07.79.−v Scanning probe microscopes and components *(see also 68.37.−d in surfaces and interfaces)*

07.79.Cz Scanning tunneling microscopes

07.79.Fc Near-field scanning optical microscopes

07.79.Lh Atomic force microscopes

07.79.Pk Magnetic force microscopes

07.79.Sp Friction force microscopes

07.81.+a Electron and ion spectrometers *(see also 29.30.−h in nuclear physics)*

07.85.−m X- and γ-ray instruments *(for x- and γ-ray telescopes, see 95.55.Ka in astronomy)*

07.85.Fv X- and γ-ray sources, mirrors, gratings, and detectors

07.85.Jy Diffractometers

07.85.Nc X-ray and γ-ray spectrometers

07.85.Qe Synchrotron radiation instrumentation

07.85.Tt X-ray microscopes

07.87.+v Spaceborne and space research instruments, apparatus, and components (satellites, space vehicles, etc.) *(for aeronomy and magnetospheric instrumentation, see 94.80.+g; see also 95.55.Fw and 95.40.+s in astronomy)*

07.88.+y Instruments for environmental pollution measurements

07.89.+b Environmental effects on instruments (e.g., radiation and pollution effects) *(for environmental effects on optical elements, devices, and systems, see 42.88.+h)*

07.90.+c Other topics in instruments, apparatus, and components common to several branches of physics and astronomy (restricted to new topics in section 07)

10. THE PHYSICS OF ELEMENTARY PARTICLES AND FIELDS *(for cosmic rays, see 96.40.−z in astronomy; for experimental methods and instrumentation for elementary-particle physics, see section 29)*

11. General theory of fields and particles *(see also 03.65.−w Quantum mechanics and 03.70.+k Theory of quantized fields)*

11.10.−z **Field theory** *(for gauge field theories, see 11.15.−q)*
11.10.Cd Axiomatic approach
11.10.Ef Lagrangian and Hamiltonian approach
11.10.Gh Renormalization
11.10.Hi Renormalization group evolution of parameters
11.10.Jj Asymptotic problems and properties
11.10.Kk Field theories in dimensions other than four *(see also 04.50.+h Gravity in more than four dimensions; 04.60.Kz Lower dimensional models in quantum gravity)*
11.10.Lm Nonlinear or nonlocal theories and models *(see also 11.27.+d Extended classical solutions; cosmic strings, domain walls, texture)*
11.10.Nx Noncommutative field theory
11.10.St Bound and unstable states; Bethe−Salpeter equations
11.10.Wx Finite-temperature field theory
. . . . *Relativistic wave equations, see 03.65.Pm*

11.15.−q **Gauge field theories**
11.15.Bt General properties of perturbation theory
11.15.Ex Spontaneous breaking of gauge symmetries
11.15.Ha Lattice gauge theory *(see also 12.38.Gc Lattice QCD calculations)*
11.15.Kc Classical and semiclassical techniques
11.15.Me Strong-coupling expansions
11.15.Pg Expansions for large numbers of components (e.g., $1/N_c$ expansions)
11.15.Tk Other nonperturbative techniques

11.25.−w **Strings and branes** *(for cosmic strings, see 98.80.Cq in cosmology; see also 11.27.+d Extended classical solutions; cosmic strings, domain walls, texture)*
11.25.Db Properties of perturbation theory
11.25.Hf Conformal field theory, algebraic structures
11.25.Mj Compactification and four-dimensional models
11.25.Pm Noncritical string theory
11.25.Sq Nonperturbative techniques; string field theory
11.25.Tq Gauge/string duality
11.25.Uv D branes
11.25.Wx String and brane phenomenology
11.25.Yb M theory

11.27.+d **Extended classical solutions; cosmic strings, domain walls, texture** *(see also 98.80.Cq in cosmology; 11.25.−w Strings and branes)*

11.30.−j **Symmetry and conservation laws** *(see also 02.20.−a Group theory)*
11.30.Cp Lorentz and Poincaré invariance
11.30.Er Charge conjugation, parity, time reversal, and other discrete symmetries
11.30.Fs Global symmetries (e.g., baryon number, lepton number)
11.30.Hv Flavor symmetries
11.30.Ly Other internal and higher symmetries
11.30.Na Nonlinear and dynamical symmetries (spectrum-generating symmetries)
11.30.Pb Supersymmetry *(see also 12.60.Jv Supersymmetric models)*
11.30.Qc Spontaneous and radiative symmetry breaking
11.30.Rd Chiral symmetries

11.40.−q **Currents and their properties**
11.40.Dw General theory of currents
11.40.Ex Formal properties of current algebras *(see also 12.39.Fe Chiral Lagrangians)*
11.40.Ha Partially conserved axial-vector currents

11.55.−m **S-matrix theory; analytic structure of amplitudes**
11.55.Bq Analytic properties of S matrix
11.55.Ds Exact S matrices
11.55.Fv Dispersion relations
11.55.Hx Sum rules
11.55.Jy Regge formalism *(see also 12.40.Nn in strong interactions)*

11.80.−m **Relativistic scattering theory**
11.80.Cr Kinematical properties (helicity and invariant amplitudes, kinematic singularities, etc.)
11.80.Et Partial-wave analysis
11.80.Fv Approximations (eikonal approximation, variational principles, etc.)
11.80.Gw Multichannel scattering
11.80.Jy Many-body scattering and Faddeev equation
11.80.La Multiple scattering

11.90.+t **Other topics in general theory of fields and particles (restricted to new topics in section 11)**

12. Specific theories and interaction models; particle systematics

12.10.−g **Unified field theories and models** *(see also 04.50.+h—in general relativity and gravitation, 11.25.Mj Compactification and four-dimensional models)*
12.10.Dm Unified theories and models of strong and electroweak interactions
12.10.Kt Unification of couplings; mass relations

12.15.−y **Electroweak interactions**
. . . . *Extensions of gauge or Higgs sector, see 12.60.Cn or 12.60.Fr*
12.15.Ff Quark and lepton masses and mixing *(see also 14.60.Pq Neutrino mass and mixing)*
12.15.Hh Determination of Kobayashi−Maskawa matrix elements
12.15.Ji Applications of electroweak models to specific processes
12.15.Lk Electroweak radiative corrections *(see also 13.40.Ks Electromagnetic corrections to strong- and weak-interaction processes)*
12.15.Mm Neutral currents

12.20.−m **Quantum electrodynamics**
12.20.Ds Specific calculations
12.20.Fv Experimental tests *(for optical tests in quantum electrodynamics, see 42.50.Xa)*

12.38.−t **Quantum chromodynamics**
. . . . *Quarks, gluons, and QCD in nuclei and nuclear processes, see 24.85.+p*
12.38.Aw General properties of QCD (dynamics, confinement, etc.)
12.38.Bx Perturbative calculations
12.38.Cy Summation of perturbation theory
12.38.Gc Lattice QCD calculations *(see also 11.15.Ha Lattice gauge theory)*
12.38.Lg Other nonperturbative calculations
12.38.Mh Quark−gluon plasma *(see also 25.75.Nq Quark deconfinement, quark−gluon plasma production and phase transitions in relativistic heavy ion collisions)*
12.38.Qk Experimental tests

12.39.−x **Phenomenological quark models**
12.39.Ba Bag model

12.39.Dc *Skyrmions*
12.39.Fe Chiral Lagrangians
12.39.Hg Heavy quark effective theory
12.39.Jh Nonrelativistic quark model
12.39.Ki Relativistic quark model
12.39.Mk Glueball and nonstandard multi-quark/gluon states
12.39.Pn Potential models
12.39.St Factorization

12.40.−y Other models for strong interactions
12.40.Ee Statistical models
12.40.Nn Regge theory, duality, absorptive/optical models *(see also 11.55.Jy Regge formalism)*
12.40.Vv Vector-meson dominance
12.40.Yx Hadron mass models and calculations

12.60.−i Models beyond the standard model
. . . . *Unified field theories and models, see 12.10.−g*
12.60.Cn Extensions of electroweak gauge sector
12.60.Fr Extensions of electroweak Higgs sector
12.60.Jv Supersymmetric models *(see also 04.65.+e Supergravity)*
12.60.Nz Technicolor models
12.60.Rc Composite models

12.90.+b Miscellaneous theoretical ideas and models (restricted to new topics in section 12)

13. Specific reactions and phenomenology

13.15.+g Neutrino interactions

13.20.−v Leptonic, semileptonic, and radiative decays of mesons
13.20.Cz Decays of π mesons
13.20.Eb Decays of K mesons
13.20.Fc Decays of charmed mesons
13.20.Gd Decays of J/ψ, Υ, and other quarkonia
13.20.He Decays of bottom mesons
13.20.Jf Decays of other mesons

13.25.−k Hadronic decays of mesons
13.25.Cq Decays of π mesons
13.25.Es Decays of K mesons
13.25.Ft Decays of charmed mesons
13.25.Gv Decays of J/ψ, Υ, and other quarkonia
13.25.Hw Decays of bottom mesons
13.25.Jx Decays of other mesons

13.30.−a Decays of baryons
13.30.Ce Leptonic, semileptonic, and radiative decays
13.30.Eg Hadronic decays

13.35.−r Decays of leptons
13.35.Bv Decays of muons
13.35.Dx Decays of taus
13.35.Hb Decays of heavy neutrinos

13.38.−b Decays of intermediate bosons
13.38.Be Decays of W bosons
13.38.Dg Decays of Z bosons

13.40.−f Electromagnetic processes and properties
13.40.Dk Electromagnetic mass differences
13.40.Em Electric and magnetic moments
13.40.Gp Electromagnetic form factors
13.40.Hq Electromagnetic decays
13.40.Ks Electromagnetic corrections to strong- and weak-interaction processes

13.60.−r Photon and charged-lepton interactions with hadrons *(for neutrino interactions, see 13.15.+g)*
13.60.Fz Elastic and Compton scattering
13.60.Hb Total and inclusive cross sections (including deep-inelastic processes)
13.60.Le Meson production
13.60.Rj Baryon production

13.66.−a Lepton-lepton interactions
13.66.Bc Hadron production in ee$^+$ interactions
13.66.De Lepton production in ee$^+$ interactions
13.66.Fg Gauge and Higgs boson production in ee$^+$ interactions
13.66.Hk Production of non-standard model particles in ee$^+$ interactions
13.66.Jn Precision mesurements in ee$^+$ interactions
13.66.Lm Processes in other lepton-lepton interactions

13.75.−n Hadron-induced low- and intermediate-energy reactions and scattering (energy $\leqslant$ 10 GeV) *(for higher energies, see 13.85.−t)*
13.75.Cs Nucleon–nucleon interactions (including antinucleons, deuterons, etc.) *(for N–N interactions in nuclei, see 21.30.−x)*
13.75.Ev Hyperon–nucleon interactions
13.75.Gx Pion–baryon interactions
13.75.Jz Kaon–baryon interactions
13.75.Lb Meson–meson interactions

13.85.−t Hadron-induced high- and super-high-energy interactions (energy > 10 GeV) *(for low energies, see 13.75.−n)*
13.85.Dz Elastic scattering
13.85.Fb Inelastic scattering: two-particle final states
13.85.Hd Inelastic scattering: many-particle final states
13.85.Lg Total cross sections
13.85.Ni Inclusive production with identified hadrons
13.85.Qk Inclusive production with identified leptons, photons, or other nonhadronic particles
13.85.Rm Limits on production of particles
13.85.Tp Cosmic-ray interactions *(see also 96.40.−z Cosmic rays in astronomy)*

13.87.−a Jets in large-Q^2 scattering
13.87.Ce Production
13.87.Fh Fragmentation into hadrons

13.88.+e Polarization in interactions and scattering

13.90.+i Other topics in specific reactions and phenomenology of elementary particles (restricted to new topics in section 13)

14. Properties of specific particles

14.20.−c Baryons (including antiparticles)
14.20.Dh Protons and neutrons
14.20.Gk Baryon resonances with S=0
14.20.Jn Hyperons
14.20.Lq Charmed baryons
14.20.Mr Bottom baryons
14.20.Pt Dibaryons

14.40.−n Mesons
14.40.Aq Π, K, and η mesons
14.40.Cs Other mesons with S=C=0, mass < 2.5 GeV
14.40.Ev Other strange mesons
14.40.Gx Mesons with S=C=B=0, mass > 2.5 GeV (including quarkonia)
14.40.Lb Charmed mesons
14.40.Nd Bottom mesons

14.60.−z Leptons
14.60.Cd Electrons (including positrons)
14.60.Ef Muons
14.60.Fg Taus
14.60.Hi Other charged heavy leptons
14.60.Lm Ordinary neutrinos (ν_e, ν, ν_τ)
14.60.Pq Neutrino mass and mixing *(see also 12.15.Ff Quark and lepton masses and mixing)*
14.60.St Non-standard-model neutrinos, right-handed neutrinos, etc.

14.65.−q Quarks
14.65.Bt Light quarks
14.65.Dw Charmed quarks
14.65.Fy Bottom quarks
14.65.Ha Top quarks

14.70.−e Gauge bosons
14.70.Bh Photons
14.70.Dj Gluons
14.70.Fm W bosons
14.70.Hp Z bosons

14.70.Pw Other gauge bosons

14.80.−j Other particles (including hypothetical)

14.80.Bn Standard-model Higgs bosons

14.80.Cp Non-standard-model Higgs bosons

14.80.Hv Magnetic monopoles

14.80.Ly Supersymmetric partners of known particles

14.80.Mz Axions and other Nambu−Goldstone bosons (Majorons, familons, etc.)

20. NUCLEAR PHYSICS

21. Nuclear structure *(for nucleon structure, see 14.20.Dh Properties of protons and neutrons; 13.40.−f for electromagnetic processes and properties; 13.60.Hb for deep-inelastic structure functions)*

21.10.−k Properties of nuclei; nuclear energy levels *(for properties of specific nuclei listed by mass ranges, see section 27)*

21.10.Dr Binding energies and masses
21.10.Ft Charge distribution
21.10.Gv Mass and neutron distributions
21.10.Hw Spin, parity, and isobaric spin
21.10.Jx Spectroscopic factors
21.10.Ky Electromagnetic moments
21.10.Ma Level density
21.10.Pc Single-particle levels and strength functions
21.10.Re Collective levels
21.10.Sf Coulomb energies
21.10.Tg Lifetimes

21.30.−x Nuclear forces *(see also 13.75.Cs Nucleon–nucleon interactions)*

21.30.Cb Nuclear forces in vacuum
21.30.Fe Forces in hadronic systems and effective interactions

21.45.+v Few-body systems

21.60.−n Nuclear structure models and methods

21.60.Cs Shell model
21.60.Ev Collective models
21.60.Fw Models based on group theory
21.60.Gx Cluster models
21.60.Jz Hartree–Fock and random-phase approximations
21.60.Ka Monte Carlo models

21.65.+f Nuclear matter

. . . . *Exotic atoms and molecules, see 36.10.−k*

21.80.+a Hypernuclei

21.90.+f Other topics in nuclear structure (restricted to new topics in section 21)

23. Radioactive decay and in-beam spectroscopy

23.20.−g Electromagnetic transitions

23.20.En Angular distribution and correlation measurements
23.20.Gq Multipole mixing ratios
23.20.Js Multipole matrix elements
23.20.Lv γ transitions and level energies
23.20.Nx Internal conversion and extranuclear effects
23.20.Ra Internal pair production

23.40.−s decay; double β decay; electron and muon capture

23.40.Bw Weak-interaction and lepton (including neutrino) aspects *(see also 14.60.Pq Neutrino mass and mixing)*
23.40.Hc Relation with nuclear matrix elements and nuclear structure

23.50.+z Decay by proton emission

23.60.+e decay

23.70.+j Heavy-particle decay

23.90.+w Other topics in radioactive decay and in-beam spectroscopy (restricted to new topics in section 23)

24. Nuclear reactions: general

24.10.−i Nuclear reaction models and methods

24.10.Cn Many-body theory
24.10.Eq Coupled-channel and distorted-wave models
24.10.Ht Optical and diffraction models
24.10.Jv Relativistic models
24.10.Lx Monte Carlo simulations (including hadron and parton cascades and string breaking models)
24.10.Nz Hydrodynamic models
24.10.Pa Thermal and statistical models

24.30.−v Resonance reactions

24.30.Cz Giant resonances
24.30.Gd Other resonances

24.50.+g Direct reactions

24.60.−k Statistical theory and fluctuations

24.60.Dr Statistical compound-nucleus reactions
24.60.Gv Statistical multistep direct reactions
24.60.Ky Fluctuation phenomena
24.60.Lz Chaos in nuclear systems

24.70.+s Polarization phenomena in reactions

24.75.+i General properties of fission

24.80.+y Nuclear tests of fundamental interactions and symmetries

24.85.+p Quarks, gluons, and QCD in nuclei and nuclear processes

24.90.+d Other topics in nuclear reactions: general (restricted to new topics in section 24)

25. Nuclear reactions: specific reactions

25.10.+s Nuclear reactions involving few-nucleon systems

25.20.−x Photonuclear reactions

25.20.Dc Photon absorption and scattering
25.20.Lj Photoproduction reactions

25.30.−c Lepton-induced reactions

25.30.Bf Elastic electron scattering
25.30.Dh Inelastic electron scattering to specific states
25.30.Fj Inelastic electron scattering to continuum
25.30.Hm Positron scattering
25.30.Mr Muon scattering (including the EMC effect)
25.30.Pt Neutrino scattering
25.30.Rw Electroproduction reactions

25.40.−h Nucleon-induced reactions *(see also 28.20.−v Neutron physics)*

25.40.Cm Elastic proton scattering
25.40.Dn Elastic neutron scattering
25.40.Ep Inelastic proton scattering
25.40.Fq Inelastic neutron scattering
25.40.Hs Transfer reactions
25.40.Kv Charge-exchange reactions
25.40.Lw Radiative capture
25.40.Ny Resonance reactions
25.40.Qa (p, π) reactions
25.40.Sc Spallation reactions
25.40.Ve Other reactions above meson production thresholds (energies $>$ 400 MeV)

25.43.+t Antiproton-induced reactions

25.45.−z ^{2}H-induced reactions

25.45.De Elastic and inelastic scattering
25.45.Hi Transfer reactions
25.45.Kk Charge-exchange reactions

25.55.−e ^{3}H-, ^{3}He-, and ^{4}He-induced reactions

25.55.Ci Elastic and inelastic scattering
25.55.Hp Transfer reactions
25.55.Kr Charge-exchange reactions

25.60.−t Reactions induced by unstable nuclei

25.60.Bx Elastic scattering
25.60.Dz Interaction and reaction cross sections
25.60.Gc Breakup and momentum distributions
25.60.Je Transfer reactions
25.60.Lg Charge-exchange reactions
25.60.Pj Fusion reactions

25.70.−z Low and intermediate energy heavy-ion reactions

25.70.Bc Elastic and quasielastic scattering
25.70.De Coulomb excitation

25.70.Ef Resonances
25.70.Gh Compound nucleus
25.70.Hi Transfer reactions
25.70.Jj Fusion and fusion–fission reactions
25.70.Kk Charge-exchange reactions
25.70.Lm Strongly damped collisions
25.70.Mn Projectile and target fragmentation
25.70.Pq Multifragment emission and correlations

25.75.−q Relativistic heavy-ion collisions *(collisions induced by light ions studied to calibrate relativistic heavy-ion collisions should be classified under both 25.75.−q and sections 13 or 25 appropriate to the light ions)*
25.75.Dw Particle and resonance production
25.75.Gz Particle correlations
25.75.Ld Collective flow
25.75.Nq Quark deconfinement, quark-gluon plasma production, and phase transitions *(see also 12.38.Mh Quark–gluon plasma in quantum chromodynamics)*

25.80.−e Meson- and hyperon-induced reactions
25.80.Dj Pion elastic scattering
25.80.Ek Pion inelastic scattering
25.80.Gn Pion charge-exchange reactions
25.80.Hp Pion-induced reactions
25.80.Ls Pion inclusive scattering and absorption
25.80.Nv Kaon-induced reactions
25.80.Pw Hyperon-induced reactions

25.85.−w Fission reactions
25.85.Ca Spontaneous fission
25.85.Ec Neutron-induced fission
25.85.Ge Charged-particle-induced fission
25.85.Jg Photofission

25.90.+k Other topics in nuclear reactions: specific reactions (restricted to new topics in section 25)

26. Nuclear astrophysics *(see also 95.30.−k Fundamental aspects of astrophysics in astronomy)*

26.20.+f Hydrostatic stellar nucleosynthesis *(see also 97.10.Cv Stellar structure, interiors, evolution, nucleosynthesis, ages in astronomy)*

26.30.+k Nucleosynthesis in novae, supernovae and other explosive environments

26.35.+c Big Bang nucleosynthesis *(see also 98.80.Ft Origin, formation, and abundances of the elements in astronomy)*

26.40.+r Cosmic ray nucleosynthesis

26.50.+x Nuclear physics aspects of novae, supernovae, and other explosive environments

26.60.+c Nuclear matter aspects of neutron stars

26.65.+t Solar neutrinos

27. Properties of specific nuclei listed by mass ranges *(an additional heading must be chosen with these entries, where the given mass number limits are, to some degree, arbitrary)*

27.10.+h $A \leqslant 5$

27.20.+n $6 \leqslant A \leqslant 19$

27.30.+t $20 \leqslant A \leqslant 38$

27.40.+z $39 \leqslant A \leqslant 58$

27.50.+e $59 \leqslant A \leqslant 89$

27.60.+j $90 \leqslant A \leqslant 149$

27.70.+q $150 \leqslant A \leqslant 189$

27.80.+w $190 \leqslant A \leqslant 219$

27.90.+b $220 \leqslant A$

28. Nuclear engineering and nuclear power studies

28.20.−v Neutron physics *(see also 25.40.−h Nucleon-induced reactions and 25.85.Ec Neutron-induced fission)*
28.20.Cz Neutron scattering
28.20.Fc Neutron absorption
28.20.Gd Neutron transport: diffusion and moderation

28.41.−i Fission reactors
28.41.Ak Theory, design, and computerized simulation
28.41.Bm Fuel elements, preparation, reloading, and reprocessing
28.41.Fr Reactor coolants, reactor cooling, and heat recovery
28.41.Kw Radioactive wastes, waste disposal
28.41.My Reactor control systems
28.41.Pa Moderators
28.41.Qb Structural and shielding materials
28.41.Rc Instrumentation
28.41.Te Protection systems, safety, radiation monitoring, accidents, and dismantling

28.50.−k Fission reactor types
28.50.Dr Research reactors
28.50.Ft Fast and breeder reactors
28.50.Hw Power and production reactors
28.50.Ky Propulsion reactors
28.50.Ma Auxiliary generators

28.52.−s Fusion reactors *(see also 52.55.−s, 52.57.−z, and 52.58.−c in physics of plasmas)*
28.52.Av Theory, design, and computerized simulation
28.52.Cx Fueling, heating and ignition
28.52.Fa Materials
28.52.Lf Components and instrumentation
28.52.Nh Safety

28.60.+s Isotope separation and enrichment

28.70.+y Nuclear explosions *(see also 47.40.−x Compressional flows; shock and detonation phenomena; for radiation protection from fallout, see 87.52.−g in biological and medical physics)*

28.90.+i Other topics in nuclear engineering and nuclear power studies (restricted to new topics in section 28)

29. Experimental methods and instrumentation for elementary-particle and nuclear physics

29.17.+w Electrostatic, collective, and linear accelerators

29.20.−c Cyclic accelerators and storage rings
29.20.Dh Storage rings
29.20.Fj Betatrons
29.20.Hm Cyclotrons
29.20.Lq Synchrotrons

29.25.−t Particle sources and targets *(see also 52.59.−f in physics of plasmas)*
29.25.Bx Electron sources
29.25.Dz Neutron sources
29.25.Lg Ion sources: polarized
29.25.Ni Ion sources: positive and negative
29.25.Pj Polarized and other targets
29.25.Rm Sources of radioactive nuclei

29.27.−a Beams in particle accelerators *(for low energy charged-particle beams, see 41.75.−i)*
29.27.Ac Beam injection and extraction
29.27.Bd Beam dynamics; collective effects and instabilities
29.27.Eg Beam handling; beam transport
29.27.Fh Beam characteristics
29.27.Hj Polarized beams

29.30.−h Spectrometers and spectroscopic techniques
29.30.Aj Charged-particle spectrometers: electric and magnetic
29.30.Dn Electron spectroscopy
29.30.Ep Charged-particle spectroscopy
29.30.Hs Neutron spectroscopy
29.30.Kv X- and γ-ray spectroscopy

29.30.Lw Nuclear orientation devices

. . . . *Energy loss and stopping power, see 34.50.Bw and 61.85.+p in atomic and molecular physics and condensed matter, respectively)*

29.40.−n Radiation detectors *(for mass spectrometers, see 07.75.+h)*

29.40.Cs Gas-filled counters: ionization chambers, proportional, and avalanche counters

29.40.Gx Tracking and position-sensitive detectors

29.40.Ka Cherenkov detectors

29.40.Mc Scintillation detectors

29.40.Rg Nuclear emulsions

29.40.Vj Calorimeters

29.40.Wk Solid-state detectors

29.50.+v Computer interfaces *(see also 07.05.Wr in computers in experimental physics)*

29.85.+c Computer data analysis

29.90.+r Other topics in elementary-particle and nuclear physics experimental methods and instrumentation (restricted to new topics in section 29)

30. ATOMIC AND MOLECULAR PHYSICS

31. Electronic structure of atoms and molecules: theory

31.10.+z Theory of electronic structure, electronic transitions, and chemical binding

31.15.−p Calculations and mathematical techniques in atomic and molecular physics (excluding electron correlation calculations) *(see also 02.70.−c computational techniques, in mathematical methods in physics)*

31.15.Ar Ab initio calculations
31.15.Bs Statistical model calculations (including Thomas–Fermi and Thomas–Fermi–Dirac models)
31.15.Ct Semi-empirical and empirical calculations (differential overlap, Hückel, PPP methods, etc.)
31.15.Dv Coupled-cluster theory
31.15.Ew Density-functional theory
31.15.Fx Finite-difference schemes
31.15.Gy Semiclassical methods
31.15.Hz Group theory
31.15.Ja Hyperspherical methods
31.15.Kb Path-integral methods
31.15.Lc Quasiparticle methods
31.15.Md Perturbation theory
31.15.Ne Self-consistent-field methods
31.15.Pf Variational techniques
31.15.Qg Molecular dynamics and other numerical methods
31.15.Rh Valence bond calculations

31.25.−v Electron correlation calculations for atoms and molecules

31.25.Eb Electron correlation calculations for atoms and ions: ground state
31.25.Jf Electron correlation calculations for atoms and ions: excited states
31.25.Nj Electron correlation calculations for diatomic molecules
31.25.Qm Electron correlation calculations for polyatomic molecules

31.30.−i Corrections to electronic structure

31.30.Gs Hyperfine interactions and isotope effects, Jahn–Teller effect
31.30.Jv Relativistic and quantum electrodynamic effects in atoms and molecules

31.50.−x Potential energy surfaces *(for potential energy surfaces for chemical reactions, see 82.20.Kh; for collisions, see 34.20.Mq)*

31.50.Bc Potential energy surfaces for ground electronic states
31.50.Df Potential energy surfaces for excited electronic states
31.50.Gh Surface crossings, non-adiabatic couplings

31.70.−f Effects of atomic and molecular interactions on electronic structure *(see also section 34 Atomic and molecular collision processes and interactions)*

31.70.Dk Environmental and solvent effects
31.70.Hq Time-dependent phenomena: excitation and relaxation processes, and reaction rates *(for chemical kinetics aspects, see 82.20.Rp)*
31.70.Ks Molecular solids

31.90.+s Other topics in the theory of the electronic structure of atoms and molecules (restricted to new topics in section 31)

32. Atomic properties and interactions with photons

32.10.−f Properties of atoms

32.10.Bi Atomic masses, mass spectra, abundances, and isotopes *(for mass spectroscopy, see 07.75.+h in instruments, and 82.80.Ms, Nj, Rt in physical chemistry and chemical physics)*
32.10.Dk Electric and magnetic moments, polarizability
32.10.Fn Fine and hyperfine structure
32.10.Hq Ionization potentials, electron affinities

32.30.−r Atomic spectra

32.30.Bv Radio-frequency, microwave, and infrared spectra
32.30.Dx Magnetic resonance spectra
32.30.Jc Visible and ultraviolet spectra
32.30.Rj X-ray spectra

32.50.+d Fluorescence, phosphorescence (including quenching)

32.60.+i Zeeman and Stark effects

32.70.−n Intensities and shapes of atomic spectral lines

32.70.Cs Oscillator strengths, lifetimes, transition moments
32.70.Fw Absolute and relative intensities
32.70.Jz Line shapes, widths, and shifts

32.80.−t Photon interactions with atoms *(see also 42.50.−p Quantum optics)*

32.80.Bx Level crossing and optical pumping
32.80.Cy Atomic scattering, cross sections, and form factors; Compton scattering
32.80.Dz Autoionization
32.80.Fb Photoionization of atoms and ions
32.80.Gc Photodetachment of atomic negative ions
32.80.Hd Auger effect and inner-shell excitation or ionization
32.80.Lg Mechanical effects of light on atoms, molecules, and ions
32.80.Pj Optical cooling of atoms; trapping
32.80.Qk Coherent control of atomic interactions with photons
32.80.Rm Multiphoton ionization and excitation to highly excited states (e.g., Rydberg states)
32.80.Wr Other multiphoton processes
32.80.Ys Weak-interaction effects in atoms

32.90.+a Other topics in atomic properties and interactions of atoms with photons (restricted to new topics in section 32)

33. Molecular properties and interactions with photons

33.15.−e Properties of molecules

33.15.Bh General molecular conformation and symmetry; stereochemistry
33.15.Dj Interatomic distances and angles
33.15.Fm Bond strengths, dissociation energies
33.15.Hp Barrier heights (internal rotation, inversion, rotational isomerism, conformational dynamics)
33.15.Kr Electric and magnetic moments (and derivatives), polarizability, and magnetic susceptibility
33.15.Mt Rotation, vibration, and vibration–rotation constants
33.15.Pw Fine and hyperfine structure
33.15.Ry Ionization potentials, electron affinities, molecular core binding energy
33.15.Ta Mass spectra
33.15.Vb Correlation times in molecular dynamics

33.20.−t Molecular spectra

33.20.Bx Radio-frequency and microwave spectra
33.20.Ea Infrared spectra
33.20.Fb Raman and Rayleigh spectra (including optical scattering)
33.20.Kf Visible spectra
33.20.Lg Ultraviolet spectra
33.20.Ni Vacuum ultraviolet spectra
33.20.Rm X-ray spectra
33.20.Sn Rotational analysis
33.20.Tp Vibrational analysis
33.20.Vq Vibration–rotation analysis
33.20.Wr Vibronic, rovibronic, and rotation–electron-spin interactions

33.25.+k Nuclear resonance and relaxation

(see also 76.60.−k Nuclear magnetic resonance and relaxation in condensed matter and 82.56.−b in physical chemistry and chemical physics)

33.35.+r Electron resonance and relaxation *(see also 76.30.−v Electron paramagnetic resonance and relaxation in condensed matter)*

33.40.+f Multiple resonances (including double and higher-order resonance processes, such as double nuclear magnetic resonance, electron double resonance, and microwave optical double resonance) *(see also 76.70.−r Magnetic double resonances and cross effects in condensed matter)*

33.45.+x Mössbauer spectra *(see also 76.80.+y Mössbauer effect; other x-ray spectroscopy)*

33.50.−j Fluorescence and phosphorescence; radiationless transitions, quenching (intersystem crossing, internal conversion) *(for energy transfer, see also section 34)*

33.50.Dq Fluorescence and phosphorescence spectra

33.50.Hv Radiationless transitions, quenching

33.55.−b Optical activity and dichroism; magnetooptical and electrooptical spectra

33.55.Ad Optical activity, optical rotation; circular dichroism

33.55.Be Zeeman and Stark effects

33.55.Fi Other magnetooptical and electrooptical effects

33.60.−q Photoelectron spectra

33.60.Cv Ultraviolet and vacuum ultraviolet photoelectron spectra

33.60.Fy X-ray photoelectron spectra

33.70.−w Intensities and shapes of molecular spectral lines and bands

33.70.Ca Oscillator and band strengths, lifetimes, transition moments, and Franck–Condon factors

33.70.Fd Absolute and relative line and band intensities

33.70.Jg Line and band widths, shapes, and shifts

33.80.−b Photon interactions with molecules *(see also 42.50.−p Quantum optics)*

33.80.Be Level crossing and optical pumping

33.80.Eh Autoionization, photoionization, and photodetachment

33.80.Gj Diffuse spectra; predissociation, photodissociation

33.80.Ps Optical cooling of molecules; trapping

33.80.Rv Multiphoton ionization and excitation to highly excited states (e.g., Rydberg states)

33.80.Wz Other multiphoton processes

33.90.+h Other topics in molecular properties and interactions with photons (restricted to new topics in section 33)

34. Atomic and molecular collision processes and interactions *(for atomic, molecular, and ionic collisions in plasma, see 52.20.Hv)*

34.10.+x General theories and models of atomic and molecular collisions and interactions (including statistical theories, transition state, stochastic and trajectory models, etc.)

34.20.−b Interatomic and intermolecular potentials and forces, potential energy surfaces for collisions

34.20.Cf Interatomic potentials and forces

34.20.Gj Intermolecular and atom–molecule potentials and forces

34.20.Mq Potential energy surfaces for collisions *(see also 82.20.Kh Potential energy surfaces for chemical reactions; for potential energy surface in electronic structure calculations, see 31.50.−x)*

34.30.+h Intramolecular energy transfer; intramolecular dynamics; dynamics of van der Waals molecules

34.50.−s Scattering of atoms and molecules

34.50.Bw Energy loss and stopping power

34.50.Dy Interactions of atoms and molecules with surfaces; photon and electron emission; neutralization of ions *(for surface characterization by particle-surface scattering, see 68.49.−h in surfaces, interfaces, thin films, and low-dimensional structures)*

34.50.Ez Rotational and vibrational energy transfer

34.50.Fa Electronic excitation and ionization of atoms (including beam–foil excitation and ionization)

34.50.Gb Electronic excitation and ionization of molecules; intermediate molecular states (including lifetimes, state mixing, etc.)

34.50.Lf Chemical reactions, energy disposal, and angular distribution, as studied by atomic and molecular beams

34.50.Pi State-to-state scattering analyses

34.50.Rk Laser-modified scattering and reactions

34.60.+z Scattering in highly excited states (e.g., Rydberg states)

34.70.+e Charge transfer *(for charge transfer reactions, see 82.30.Fi in physical chemistry and chemical physics)*

34.80.−i Electron scattering *(for electron collisions in plasma, see 52.20.Fs in physics of plasmas)*

34.80.Bm Elastic scattering of electrons by atoms and molecules

34.80.Dp Atomic excitation and ionization by electron impact

34.80.Gs Molecular excitation and ionization by electron impact

34.80.Ht Dissociation and dissociative attachment by electron impact

34.80.Kw Electron–ion scattering; excitation and ionization

34.80.Lx Electron–ion recombination and electron attachment

34.80.My Fundamental electron inelastic processes in weakly ionized gases

34.80.Nz Spin dependence of cross sections; polarized electron beam experiments

34.80.Pa Coherence and correlation in electron scattering

34.80.Qb Laser-modified scattering

34.85.+x Positron scattering

34.90.+q Other topics in atomic and molecular collision processes and interactions (restricted to new topics in section 34)

36. Exotic atoms and molecules; macromolecules; clusters

36.10.−k Exotic atoms and molecules (containing mesons, muons, and other unusual particles)

36.10.Dr Positronium, muonium, muonic atoms and molecules

36.10.Gv Mesonic atoms and molecules, hyperonic atoms and molecules

36.20.−r Macromolecules and polymer molecules *(for polymer reactions and polymerization, see 82.35.−x; for biological macromolecules and polymers, see 87.14.−g and 87.15.−v)*

36.20.Cw Molecular weights, dispersity

36.20.Ey Conformation (statistics and dynamics)

36.20.Fz Constitution (chains and sequences)

36.20.Hb Configuration (bonds, dimensions)

36.20.Kd Electronic structure and spectra

36.20.Ng Vibrational and rotational structure, infrared and Raman spectra

36.40.−c Atomic and molecular clusters

(see also 61.46.+w in condensed matter)

36.40.Cg Electronic and magnetic properties of clusters

36.40.Ei Phase transitions in clusters

36.40.Gk Plasma and collective effects in clusters

36.40.Jn Reactivity of clusters

36.40.Mr Spectroscopy and geometrical structure of clusters

36.40.Qv Stability and fragmentation of clusters

36.40.Sx Diffusion and dynamics of clusters

36.40.Vz Optical properties of clusters

36.40.Wa Charged clusters

36.90.+f Other exotic atoms and molecules; macromolecules; clusters (restricted to new topics in section 36)

39. Instrumentation and techniques for atomic and molecular physics

39.10.+j Atomic and molecular beam sources and techniques

39.20.+q Atom interferometry techniques *(see also 03.75.−b Matter waves, and 03.75.Dg Atom and neutron interferometry in quantum mechanics)*

39.25.+k Atom manipulation (scanning probe microscopy, laser cooling, etc.) *(see also 82.37.Gk STM and AFM manipulations of a single molecule in physical chemistry and chemical physics; for atom manipulation in nanofabrication and processing, see 81.16.Ta)*

39.30.+w Spectroscopic techniques *(see also 78.47.+p Time-resolved optical spectroscopies and other ultrafast optical measurements in condensed matter and 82.53.Kp Coherent spectroscopy of atoms and molecules in physical chemistry and chemical physics)*

39.90.+d Other instrumentation and techniques for atomic and molecular physics (restricted to new topics in section 39)

40. ELECTROMAGNETISM, OPTICS, ACOUSTICS, HEAT TRANSFER, CLASSICAL MECHANICS, AND FLUID MECHANICS

41. Electromagnetism; electron and ion optics

41.20.−q Applied classical electromagnetism

41.20.Cv Electrostatics; Poisson and Laplace equations, boundary-value problems

41.20.Gz Magnetostatics; magnetic shielding, magnetic induction, boundary-value problems

41.20.Jb Electromagnetic wave propagation; radiowave propagation *(for light propagation, see 42.25.Bs; for electromagnetic waves in plasma, see 52.35.Hr; for ionospheric and magnetospheric propagation, see 94.20.Bb and 94.30.Tt)*

41.50.+h X-ray beams and x-ray optics *(see also 07.85.Fv in instruments)*

41.60.−m Radiation by moving charges

41.60.Ap Synchrotron radiation *(for synchrotron radiation instrumentation, see 07.85.Qe)*

41.60.Bq Cherenkov radiation

41.60.Cr Free-electron lasers *(see also 52.59.Rz Free-electron devices—in plasma physics)*

41.75.−i Charged-particle beams

41.75.Ak Positive-ion beams

41.75.Cn Negative-ion beams

41.75.Fr Electron and positron beams

41.75.Ht Relativistic electron and positron beams

41.75.Jv Laser-driven acceleration *(see also 52.38.−r Laser-plasma interactions in plasma physics)*

41.75.Lx Other advanced accelerator concepts

41.85.−p Beam optics *(see also 07.77.Ka Charged-particle beam sources and detectors; 29.27.−a Beams in particle accelerators)*

41.85.Ar Beam extraction, beam injection

41.85.Ct Beam shaping, beam splitting

41.85.Ew Beam profile, beam intensity

41.85.Gy Chromatic and geometrical aberrations

41.85.Ja Beam transport

41.85.Lc Beam focusing and bending magnets, wiggler magnets, and quadrupoles *(see also 07.55.Db—in instruments; for superconducting magnets, see 84.71.Ba)*

41.85.Ne Electrostatic lenses, septa

41.85.Qg Beam analyzers, beam monitors, and Faraday cups

41.85.Si Beam collimators, monochromators

41.90.+e Other topics in electromagnetism; electron and ion optics (restricted to new topics in section 41)

42. Optics *(for optical properties of gases, see 51.70.+f; for optical properties of bulk materials and thin films, see 78.20.−e; for x-ray optics, see 41.50.+h)*

42.15.−i Geometrical optics

42.15.Dp Wave fronts and ray tracing

42.15.Eq Optical system design

42.15.Fr Aberrations

42.25.−p Wave optics

42.25.Bs Wave propagation, transmission and absorption *(see also 41.20.Jb—in electromagnetism; for propagation in atmosphere, see 42.68.Ay; see also 52.40.Db Electromagnetic (nonlaser) radiation interactions with plasma and 52.38-r Laser-plasma interactions—in plasma physics)*

42.25.Dd Wave propagation in random media

42.25.Fx Diffraction and scattering

42.25.Gy Edge and boundary effects; reflection and refraction

42.25.Hz Interference

42.25.Ja Polarization

42.25.Kb Coherence

42.25.Lc Birefringence

42.30.−d Imaging and optical processing

42.30.Kq Fourier optics

42.30.Lr Modulation and optical transfer functions

42.30.Ms Speckle and moire patterns

42.30.Rx Phase retrieval

42.30.Sy Pattern recognition

42.30.Tz Computer vision; robotic vision

42.30.Va Image forming and processing

42.30.Wb Image reconstruction; tomography

42.40.−i Holography

42.40.Eq Holographic optical elements; holographic gratings

42.40.Ht Hologram recording and readout methods *(see also 42.70.Ln Holographic recording materials; optical storage media)*

42.40.Jv Computer-generated holograms

42.40.Kw Holographic interferometry; other holographic techniques *(see also 07.60.Ly Interferometers)*

42.40.Lx Diffraction efficiency, resolution, and other hologram characteristics

42.40.My Applications

42.40.Pa Volume holograms

42.50.−p Quantum optics *(for lasers, see 42.55.−f and 42.60.−v; see also 42.65.−k Nonlinear optics; 03.65.−w Quantum mechanics)*

42.50.Ar Photon statistics and coherence theory

42.50.Ct Quantum description of interaction of light and matter; related experiments

42.50.Dv Nonclassical states of the electromagnetic field, including entangled photon states; quantum state engineering and measurements *(see also 03.65.Ud Entanglement and quantum nonlocality (e.g. EPR paradox, Bell's inequalities, GHZ states, etc.)*

42.50.Fx Cooperative phenomena in quantum optical systems

42.50.Gy Effects of atomic coherence on propagation, absorption, and amplification of light; electromagnetically induced transparency and absorption

42.50.Hz Strong-field excitation of optical transitions in quantum systems; multi-photon processes; dynamic Stark shift *(for multiphoton ionization and excitation of atoms and molecules, see 32.80.Rn, and 33.80.Rm, respectively)*

42.50.Lc Quantum fluctuations, quantum noise, and quantum jumps

42.50.Md Optical transient phenomena: quantum beats, photon echo, free-induction decay, dephasings and revivals, optical nutation, and self-induced transparency

. . . . *Dynamics of nonlinear optical systems; optical instabilities, optical chaos, and optical spatio−temporal dynamics, see 42.65.Sf*

. . . . *Optical solitons; nonlinear guided waves, see 42.65.Tg*

42.50.Nn Quantum optical phenomena in absorbing, dispersive and conducting media

42.50.Pq Cavity quantum electrodynamics; micromasers

42.50.St Nonclassical interferometry, subwavelength lithography

42.50.Vk Mechanical effects of light on atoms, molecules, electrons, and ions *(see also 32.80.Pj and 33.80.Ps Optical cooling and trapping of atoms and molecules, respectively)*

. . . . *Optical tests of fundamental laws and forces, see 12.20.Fv Experimental tests in quantum electrodynamics and 03.65.Ta Foundations of quantum mechanics; measurement theory*

42.50.Xa Optical tests of quantum theory

42.55.−f Lasers

42.55.Ah General laser theory

42.55.Ks Chemical lasers *(for chemiluminescence, see 78.60.Ps)*

42.55.Lt Gas lasers including excimer and metal-vapor lasers

42.55.Mv Dye lasers

42.55.Px Semiconductor lasers; laser diodes

42.55.Rz Doped-insulator lasers and other solid state lasers

42.55.Sa Microcavity and microdisk lasers

42.55.Tv Photonic crystal lasers and coherent effects

42.55.Vc X- and γ-ray lasers

42.55.Wd Fiber lasers

42.55.Xi Diode-pumped lasers

42.55.Ye Raman lasers *(see also 42.65.Dr Stimulated Raman scattering; CARS)*

. . . . *Free-electron lasers, see 41.60.Cr and 52.59.Rz in electromagnetism and plasma physics, respectively*

42.55.Zz Random lasers

42.60.−v Laser optical systems: design and operation

42.60.By Design of specific laser systems

42.60.Da Resonators, cavities, amplifiers, arrays, and rings

42.60.Fc Modulation, tuning, and mode locking

42.60.Gd Q-switching

42.60.Jf Beam characteristics: profile, intensity, and power; spatial pattern formation

42.60.Lh Efficiency, stability, gain, and other operational parameters

42.60.Mi Dynamical laser instabilities; noisy laser behavior

42.60.Pk Continuous operation

42.60.Rn Relaxation oscillations and long pulse operation

. . . . *Ultrashort pulse generation, see 42.65.Tg*

. . . . *Dynamics of nonlinear optical systems, see 42.65.Sf*

42.62.−b Laser applications

42.62.Be Biological and medical applications *(see also 87.50.Hj, 87.54.Fj, 87.63.Lk, and 87.80.Cc in biological and medical physics)*

42.62.Cf Industrial applications

42.62.Eh Metrological applications; optical frequency synthesizers for precision spectroscopy *(see also 06.20.−f Metrology, and 06.30.−k Measurements common to several branches of physics and astronomy)*

42.62.Fi Laser spectroscopy

42.65.−k Nonlinear optics

42.65.An Optical susceptibility, hyperpolarizability *(see also 33.15.Mt Electric and magnetic moments, polarizability and magnetic susceptibility of molecules)*

42.65.Dr Stimulated Raman scattering; CARS *(for Raman lasers, see 42.55.Ye)*

42.65.Es Stimulated Brillouin and Rayleigh scattering

42.65.Hw Phase conjugation; photorefractive and Kerr effects

42.65.Jx Beam trapping, self-focusing and defocusing; self-phase modulation

42.65.Ky Frequency conversion; harmonic generation, including higher-order harmonic generation *(see also 42.79.Nv Optical frequency converters)*

42.65.Lm Parametric down conversion and production of entangled photons *(see also 42.50.Dv Nonclassical states of the electromagnetic field, including entangled photon states; quantum state engineering and measurements; for optical parametric oscillators and amplifiers, see 42.65.Yj)*

42.65.Pc Optical bistability, multistability, and switching, including local field effects *(see also 42.60.Gd Q-switching; 42.79.Ta Optical computers, logic elements, interconnects, switches; neural networks)*

42.65.Re Ultrafast processes; optical pulse generation and pulse compression

42.65.Sf Dynamics of nonlinear optical systems; optical instabilities, optical chaos and complexity, and optical spatio−temporal dynamics

42.65.Tg Optical solitons; nonlinear guided waves *(for solitons in fibers, see 42.81.Dp)*

42.65.Wi Nonlinear waveguides

42.65.Yj Optical parametric oscillators and amplifiers *(see also 42.65.Lm Parametric down conversion and production of entangled photons)*

42.66.−p Physiological optics

42.66.Ct Anatomy and optics of eye

42.66.Ew Physiology of eye; optic-nerve structure and function

42.66.Lc Vision: light detection, adaptation, and discrimination

42.66.Ne Color vision: color detection, adaptation, and discrimination

42.66.Qg Scales for light and color detection

42.66.Si Psychophysics of vision, visual perception; binocular vision

42.68.−w Atmospheric and ocean optics

42.68.Ay Propagation, transmission, attenuation, and radiative transfer *(see also 92.60.Ta Interaction of atmosphere with electromagnetic waves; propagation)*

42.68.Bz Atmospheric turbulence effects *(see also 92.60.Ek Convection, turbulence, and diffusion in meteorology)*

42.68.Ca Spectral absorption by atmospheric gases *(see also 94.10.Gb Absorption and scattering of radiation in physics of the neutral atmosphere)*

42.68.Ge Effects of clouds and water; ice crystal phenomena *(see also 92.60.Jq Water in the atmosphere; 92.60.Nv Cloud physics in meteorology)*

42.68.Jg Effects of aerosols *(see also 92.60.Mt Particles and aerosols in meteorology)*

42.68.Kh Effects of air pollution *(see also 92.60.Sz Air quality and air pollution in meteorology)*

42.68.Mj Scattering, polarization *(see also 94.10.Gb Absorption and scattering of radiation in physics of the neutral atmosphere)*

42.68.Sq Image transmission and formation

42.68.Wt Remote sensing; LIDAR and adaptive systems

42.68.Xy Ocean optics *(see also 92.10.Pt Optical properties of sea water in physics of the oceans)*

42.70.−a Optical materials *(see also 81.05.−t Specific materials: fabrication, treatment, testing and analysis)*

42.70.Ce Glasses, quartz

42.70.Df Liquid crystals *(for structure of liquid crystals, see 61.30.−v)*

42.70.Gi Light-sensitive materials

42.70.Hj Laser materials

42.70.Jk Polymers and organics

42.70.Km Infrared transmitting materials

42.70.Ln Holographic recording materials; optical storage media

42.70.Mp Nonlinear optical crystals *(see also 77.84.−s Dielectric, piezoelectric, and ferroelectric materials)*

42.70.Nq Other nonlinear optical materials; photorefractive and semiconductor materials

42.70.Qs Photonic bandgap materials *(for photonic crystal lasers, see 42.55.Tv)*

42.72.−g Optical sources and standards *(for lasers, see 42.55.−f; see also 07.57.Hm in instruments)*

42.72.Ai Infrared sources

42.72.Bj Visible and ultraviolet sources

42.79.−e Optical elements, devices, and systems *(for integrated optics, see 42.82.−m; for fiber optics, see 42.81.−i)*

. . . . *Optical instruments, equipment and techniques, see 07.60.−j and 07.57.−c*

. . . . *Optical spectrometers, see 07.57.Ty and 07.60.Rd*

. . . . *Photography, photographic instruments and techniques, see 07.68.+m*

. . . . *Magnetooptical devices, see 85.70.Sq*

42.79.Ag Apertures, collimators

42.79.Bh Lenses, prisms and mirrors

42.79.Ci Filters, zone plates, and polarizers

42.79.Dj Gratings *(for holographic gratings, see 42.40.Eq)*

42.79.Ek Solar collectors and concentrators *(see also 84.60.Jt Photoelectric conversion: solar cells and arrays)*

42.79.Fm Reflectors, beam splitters, and deflectors

42.79.Gn Optical waveguides and couplers *(for fiber waveguides and waveguides in integrated optics, see 42.81.Qb and 42.82.Et, respectively)*

42.79.Hp Optical processors, correlators, and modulators

42.79.Jq Acousto-optical devices *(see also 43.38.Zp—in acoustics appendix)*

42.79.Kr Display devices, liquid-crystal devices *(see also 85.60.Pg Display systems)*

42.79.Ls Scanners, image intensifiers, and image converters *(see also 85.60.−q Optoelectronic devices)*

42.79.Mt Schlieren devices

42.79.Nv Optical frequency converters

42.79.Pw Imaging detectors and sensors *(see also 85.60.Gz Photodetectors)*

42.79.Qx Range finders, remote sensing devices; laser Doppler velocimeters, SAR, and LIDAR *(see also 42.68.Wt Remote sensing; LIDAR and adaptive systems)*

42.79.Ry Gradient-index (GRIN) devices *(for fiber GRIN devices, see 42.81.Ht)*

42.79.Sz Optical communication systems, multiplexers, and demultiplexers *(for fiber networks, see 42.81.Uv)*

42.79.Ta Optical computers, logic elements, interconnects, switches; neural networks

42.79.Vb Optical storage systems, optical disks *(see also 42.40.Ht Hologram recording and readout methods)*

42.79.Wc Optical coatings

42.81.−i Fiber optics

. . . . *Fiber-optic instruments, see 07.60.Vg*

42.81.Bm Fabrication, cladding, and splicing

42.81.Cn Fiber testing and measurement of fiber parameters

42.81.Dp Propagation, scattering, and losses; solitons

42.81.Gs Birefringence, polarization

42.81.Ht Gradient-index (GRIN) fiber devices

42.81.Pa Sensors, gyros

42.81.Qb Fiber waveguides, couplers, and arrays

42.81.Uv Fiber networks *(see also 42.79.Sz Optical communication systems, multiplexers, and demultiplexers)*

42.81.Wg Other fiber-optical devices *(for fiber lasers, see 42.55.Wd)*

42.82.−m Integrated optics

42.82.Bq Design and performance testing of integrated-optical systems

42.82.Cr Fabrication techniques; lithography, pattern transfer *(see also 85.40.−e Microelectronics: LSI, VLSI, ULSI; integrated circuit fabrication technology)*

42.82.Ds Interconnects, including holographic interconnects *(see also 42.79.Td Optical computers, logic elements, interconnects, switches; neural networks)*

42.82.Et Waveguides, couplers, and arrays *(for fiber waveguides, see 42.81.Qb)*

42.82.Fv Hybrid systems

42.82.Gw Other integrated-optical elements and systems

42.86.+b Optical workshop techniques

42.87.−d Optical testing techniques

42.87.Bg Phase shifting interferometry *(see also 07.60.Ly Interferometers)*

42.88.+h Environmental and radiation effects on optical elements, devices, and systems *(see also 07.89.+b Environmental effects on instruments)*

42.90.+m Other topics in optics (restricted to new topics in section 42)

43. Acoustics *(for more detailed headings, see Appendix to section 43)*

43.20.+g General linear acoustics

43.25.+y Nonlinear acoustics

43.28.+h Aeroacoustics and atmospheric sound *(see also 92.60.−e Meteorology)*

43.30.+m Underwater sound *(see also 92.10.Vz—in physics of oceans)*

43.35.+d Ultrasonics, quantum acoustics, and physical effects of sound

. . . . *Phonons in crystal lattices, see 63.20.−e*

. . . . *Acoustical properties of rocks and minerals, see 91.60.Lj*

. . . . *Sound waves in plasma, see 52.35.Dm*

. . . . *Low-temperature acoustics and sound in liquid helium, see section 67*

. . . . *Acoustical properties of solids, see 62.65.+k; for ultrasonic relaxation, see 62.80.+f*

. . . . *Acoustic properties of thin films, see 68.60.Bs*

. . . . *Acoustoelectric effects, see 72.50.+b and 73.50.Rb*

. . . . *Magnetoacoustic effects, oscillations, and resonance, see 72.55.+s, 73.50.Rb, and 75.80.+q*

. . . . *Acoustic holography, see 43.60.Sx in acoustics appendix; for acoustooptical effects, see 78.20.Hp*

43.38.+n Transduction; acoustical devices for the generation and reproduction of sound

43.40.+s Structural acoustics and vibration

43.50.+y Noise: its effects and control

43.55.+p Architectural acoustics

43.58.+z Acoustical measurements and instrumentation

43.60.+d Acoustic signal processing

43.64.+r Physiological acoustics

. . . . *Biological effects of sound and ultrasound, see 87.50.Kk*

43.66.+y Psychological acoustics

43.70.+i Speech production

43.71.+m Speech perception

43.72.+q Speech processing and communication systems

43.75.+a Music and musical instruments

43.80.+p Bioacoustics

43.90.+v Other topics in acoustics (restricted to new topics in section 43)

44. Heat transfer

44.05.+e Analytical and numerical techniques

44.10.+i Heat conduction *(see also 66.60.+a and 66.70.+f in transport properties of condensed matter)*

44.15.+a Channel and internal heat flow

44.20.+b Boundary layer heat flow

44.25.+f Natural convection *(see also 47.27.Te Convection and heat transfer in fluid dynamics)*

44.27.+g **Forced convection**

44.30.+v **Heat flow in porous media**

44.35.+c **Heat flow in multiphase systems**

44.40.+a **Thermal radiation**

44.90.+c **Other topics in heat transfer (restricted to new topics in section 44)**

45. Classical mechanics of discrete systems

45.05.+x **General theory of classical mechanics of discrete systems**

45.10.−b **Computational methods in classical mechanics** *(see also 02.70.−c Computational techniques in mathematical methods in physics)*

45.10.Db Variational and optimization methods

45.10.Hj Perturbation and fractional calculus methods

45.10.Na Geometrical and tensorial methods

45.20.−d **Formalisms in classical mechanics**

45.20.Dd Newtonian mechanics

45.20.Jj Lagrangian and Hamiltonian mechanics

45.30.+s **General linear dynamical systems** *(for nonlinear dynamical systems, see 05.45.−a)*

45.40.−f **Dynamics and kinematics of rigid bodies**

45.40.Cc Rigid body and gyroscope motion

45.40.Gj Ballistics (projectiles; rockets)

45.40.Ln Robotics

45.50.−j **Dynamics and kinematics of a particle and a system of particles**

45.50.Dd General motion

45.50.Jf Few- and many-body systems

45.50.Pk Celestial mechanics *(see also 95.10.Ce in fundamental astronomy)*

45.50.Tn Collisions

45.70.−n **Granular systems** *(see also 05.65.+b Self-organized systems)*

45.70.Cc Static sandpiles; granular compaction

45.70.Ht Avalanches

45.70.Mg Granular flow: mixing, segregation and stratification

45.70.Qj Pattern formation

45.70.Vn Granular models of complex systems; traffic flow

45.80.+r **Control of mechanical systems** *(see also 46.80.+j Measurement methods and techniques in continuum mechanics of solids)*

45.90.+t **Other topics in classical mechanics of discrete systems (restricted to new topics in section 45)**

46. Continuum mechanics of solids *(see also 83.10.Ff in rheology)*

46.05.+b **General theory of continuum mechanics of solids**

46.15.−x **Computational methods in continuum mechanics** *(see also 02.70.−e Computational techniques in mathematical methods in physics)*

46.15.Cc Variational and optimizational methods

46.15.Ff Perturbation and complex analysis methods

46.25.−y **Static elasticity**

46.25.Cc Theoretical studies

46.25.Hf Thermoelasticity and electromagnetic elasticity (electroelasticity, magnetoelasticity)

46.32.+x **Static buckling and instability**

46.35.+z **Viscoelasticity, plasticity, viscoplasticity** *(see also 83.60.Bc, Df, in rheology)*

46.40.−f **Vibrations and mechanical waves** *(see also 43.40.+s Structural acoustics and vibration; 62.30.+d Mechanical and elastic waves; vibrations in mechanical properties of solids)*

46.40.Cd Mechanical wave propagation (including diffraction, scattering, and dispersion)

46.40.Ff Resonance, damping and dynamic stability

46.40.Jj Aeroelasticity and hydroelasticity

46.50.+a **Fracture mechanics, fatigue and cracks** *(see also 62.20.Mk Fatigue, brittleness, fracture, and cracks in mechanical properties of solids)*

46.55.+d **Tribology and mechanical contacts** *(see also 81.40.Pq Friction, lubrication and wear in materials science; 62.20.Qp Tribology and hardness in mechanical properties of solids)*

46.65.+g **Random phenomena and media** *(see also 05.40.−a in statistical physics, thermodynamics and nonlinear dynamical systems)*

46.70.−p **Application of continuum mechanics to structures**

46.70.De Beams, plates and shells

46.70.Hg Membranes, rods and strings

46.70.Lk Other structures

46.80.+j **Measurement methods and techniques in continuum mechanics of solids** *(see also 07.10.−h Mechanical instruments, equipment, and techniques)*

46.90.+s **Other topics in continuum mechanics of solids (restricted to new topics in section 46)**

47. Fluid dynamics *(for fluid dynamics of quantum fluids, see 67; see also section 83 Rheology; for sound generation by fluid flow, see section 43.28.Ra—in acoustics appendix)*

47.10.+g **General theory** *(see also 83.10.−y—in rheology)*

47.11.+j **Computational methods in fluid dynamics** *(see also 83.85.Pt Computational fluid dynamics—in rheology; 02.70.−c Computational techniques in mathematical methods in physics)*

47.15.−x **Laminar flows**

47.15.Cb Laminar boundary layers

47.15.Fe Stability of laminar flows

47.15.Gf Low-Reynolds-number (creeping) flows

47.15.Hg Potential flows

47.15.Ki Inviscid flows with vorticity

47.15.Pn Laminar suspensions

47.15.Rq Laminar flows in cavities

47.17.+e **Mechanical properties of fluids** *(see also 62.10.+s Mechanical properties of liquids)*

47.20.−k **Hydrodynamic stability**

47.20.Bp Buoyancy-driven instability

47.20.Cq Inviscid instability

47.20.Dr Surface-tension-driven instability

47.20.Ft Instability of shear flows

47.20.Gv Viscous instability

47.20.Hw Morphological instability; phase changes *(see also section 64 Equations of state, phase equilibria, and phase transitions)*

47.20.Ky Nonlinearity (including bifurcation theory)

47.20.Lz Secondary instability

47.20.Ma Interfacial instability

47.20.Pc Receptivity

. . . . *Chaotic phenomena, see 47.52.+j and 05.45.−a*

47.27.−i **Turbulent flows, convection, and heat transfer**

47.27.Ak Fundamentals

47.27.Cn Transition to turbulence

47.27.Eq Turbulence simulation and modeling

47.27.Gs Isotropic turbulence; homogeneous turbulence

47.27.Jv High-Reynolds-number turbulence

47.27.Lx Wall-bounded thin shear flows

47.27.Nz Boundary layer and shear turbulence

47.27.Pa Thick shear flows

47.27.Qb Turbulent diffusion

47.27.Rc Turbulence control

47.27.Sd Noise (turbulence generated)

47.27.Te Convection and heat transfer *(see also 44.25.+f in heat transfer)*

47.27.Vf Wakes

47.27.Wg Jets

47.32.−y Rotational flow and vorticity

47.32.Cc Vortex dynamics

47.32.Ff Separated flows

47.35.+i Hydrodynamic waves

47.37.+q Hydrodynamic aspects of superfluidity *(see also 67.40.Hf and 67.57.De-in quantum fluids and solids)*

47.40.−x Compressible flows; shock and detonation phenomena *(see also 28.70.+y Nuclear explosions; 52.35.Tc Shock waves and discontinuities in plasma; 83.60.Uv—in rheology; 43.25.Cb, 43.28.Mw and 43.40.Jc—in acoustics appendix)*

47.40.Dc General subsonic flows

47.40.Hg Transonic flows

47.40.Ki Supersonic and hypersonic flows

47.40.Nm Shock wave interactions and shock effects *(for shock wave initiated chemical reactions, see 82.40.Fp)*

47.45.−n Rarefied gas dynamics

47.45.Dt Free molecular flows

47.45.Gx Slip flows

47.45.Nd Accommodation

47.50.+d Non-Newtonian fluid flows *(see also 83.50.−v Deformation and flow)*

47.52.+j Chaos *(see also 05.45.−a Nonlinear dynamics and nonlinear dynamical systems; 83.60.Wc Flow instabilities)*

47.53.+n Fractals

47.54.+r Pattern selection; pattern formation

47.55.−t Nonhomogeneous flows

47.55.Bx Cavitation

47.55.Dz Drops and bubbles

47.55.Hd Stratified flows

· · · · *Rotational flows, see 47.32.−y*

47.55.Kf Multiphase and particle-laden flows

47.55.Mh Flows through porous media *(for heat transfer in porous media, see 44.30.+v)*

47.60.+i Flows in ducts, channels, nozzles, and conduits *(see also 83.50.Ha—in rheology)*

· · · · *Biological fluid dynamics, see 87.19.Tt*

47.62.+q Flow control

47.65.+a Magnetohydrodynamics and electrohydrodynamics *(for MHD in plasma, see 52.30.Cv)*

47.70.−n Reactive, radiative, or nonequilibrium flows

47.70.Fw Chemically reactive flows *(see also 83.80.Jx—in rheology)*

47.70.Mc Radiation gas dynamics

47.70.Nd Nonequilibrium gas dynamics

47.75.+f Relativistic fluid dynamics *(for astrophysical aspects, see 95.30.Lz and 95.30.Qd in astronomy)*

47.80.+v Instrumentation for fluid dynamics *(see also 83.85.−c—in rheology; 07.30.−t Vacuum apparatus and techniques)*

47.85.−g Applied fluid mechanics

47.85.Dh Hydrodynamics, hydraulics, hydrostatics

47.85.Gj Aerodynamics

47.85.Kn Hydraulic and pneumatic machinery

47.85.Np Fluidics

47.90.+a Other topics in fluid dynamics (restricted to new topics in section 47)

50. PHYSICS OF GASES, PLASMAS, AND ELECTRIC DISCHARGES

51. Physics of gases

51.10.+y **Kinetic and transport theory of gases** *(see also 05.20.Dd Kinetic theory in classical statistical mechanics)*

51.20.+d **Viscosity, diffusion, and thermal conductivity**

51.30.+i **Thermodynamic properties, equations of state** *(see also 05.70.Ce Thermodynamic functions and equations of state in thermodynamics)*

51.35.+a **Mechanical properties; compressibility**

51.40.+p **Acoustical properties** *(see also 43.28.−g Aeroacoustics and atmospheric sound in acoustics appendix; for ultrasonic relaxation in gases, see 43.35.Fj—in acoustics appendix)*

51.50.+v **Electrical properties (ionization, breakdown, electron and ion mobility, etc.)** *(see also 52.80.−s Electric discharges in physics of plasmas)*

51.60.+a **Magnetic properties**

51.70.+f **Optical and dielectric properties**

. . . . *Sorption, see 68.43.−h in surfaces and interfaces, thin films and low-dimensional structures*

. . . . *Gas sensors and detectors, see 07.07.Df*

51.90.+r **Other topics in the physics of gases (restricted to new topics in section 51)**

52. Physics of plasmas and electric discharges *(for astrophysical plasmas, see 95.30.Qd; for physics of the ionosphere and magnetosphere, see 94.20.−y and 94.30.−d respectively)*

52.20.−j **Elementary processes in plasmas**

52.20.Dq Particle orbits

52.20.Fs Electron collisions

52.20.Hv Atomic, molecular, ion, and heavy-particle collisions

52.25.−b **Plasma properties** *(for chemical reactions in plasma, see 82.33.Xj)*

52.25.Dg Plasma kinetic equations

52.25.Fi Transport properties

52.25.Gj Fluctuation and chaos phenomena *(for plasma turbulence, see 52.35.Ra; see also 05.45.−a Nonlinear dynamics and nonlinear dynamical systems)*

52.25.Jm Ionization of plasmas

52.25.Kn Thermodynamics of plasmas

52.25.Mq Dielectric properties

52.25.Os Emission, absorption, and scattering of electromagnetic radiation

52.25.Tx Emission, absorption, and scattering of particles

52.25.Vy Impurities in plasmas

52.25.Xz Magnetized plasmas

52.25.Ya Neutrals in plasmas

52.27.−h **Basic studies of specific kinds of plasmas**

52.27.Aj Single-component, electron-positive-ion plasmas

52.27.Cm Multicomponent and negative-ion plasmas

52.27.Ep Electron-positron plasmas

52.27.Gr Strongly-coupled plasmas

52.27.Jt Nonneutral plasmas

52.27.Lw Dusty or complex plasmas; plasma crystals

52.27.Ny Relativistic plasmas

52.30.−q **Plasma dynamics and flow**

52.30.Cv Magnetohydrodynamics (including electron magnetohydrodynamics) *(see also 47.65.+a in fluid dynamics; for MHD generators, see 52.75.Fk)*

52.30.Ex Two-fluid and multi-fluid plasmas

52.30.Gz Gyrokinetics

52.35.−g **Waves, oscillations, and instabilities in plasmas and intense beams**

52.35.Bj Magnetohydrodynamic waves (e.g., Alfven waves)

52.35.Dm Sound waves

52.35.Fp Electrostatic waves and oscillations (e.g., ion-acoustic waves)

52.35.Hr Electromagnetic waves (e.g., electron-cyclotron, Whistler, Bernstein, upper hybrid, lower hybrid)

52.35.Kt Drift waves

52.35.Lv Other linear waves

52.35.Mw Nonlinear phenomena: waves, wave propagation, and other interactions (including parametric effects, mode coupling, ponderomotive effects, etc.)

52.35.Py Macroinstabilities (hydromagnetic, e.g., kink, fire-hose, mirror, ballooning, tearing, trapped-particle, flute, Rayleigh-Taylor, etc.)

52.35.Qz Microinstabilities (ion-acoustic, two-stream, loss-cone, beam-plasma, drift, ion- or electron-cyclotron, etc.)

52.35.Ra Plasma turbulence

52.35.Sb Solitons; BGK modes

52.35.Tc Shock waves and discontinuities

52.35.Vd Magnetic reconnection

52.35.We Plasma vorticity

52.38.−r **Laser−plasma interactions** *(for plasma production and heating by laser beams, see 52.50.Jm)*

52.38.Bv Rayleigh scattering; stimulated Brillouin and Raman scattering

52.38.Dx Laser light absorption in plasmas (collisional, parametric, etc.)

52.38.Fz Laser-induced magnetic fields in plasmas

52.38.Hb Self-focussing, channeling, and filamentation in plasmas

52.38.Kd Laser-plasma acceleration of electrons and ions *(see also 41.75.Jv Laser-driven acceleration in electromagnetism; electron and ion optics)*

52.38.Mf Laser ablation *(see also 79.20.Ds, Laser-beam impact phenomena)*

52.38.Ph X-ray, γ-ray and particle generation

52.40.−w **Plasma interactions (nonlaser)**

52.40.Db Electromagnetic (nonlaser) radiation interactions with plasma

52.40.Fd Plasma interactions with antennas; plasma-filled waveguides

52.40.Hf Plasma−material interactions; boundary layer effects

52.40.Kh Plasma sheaths

52.40.Mj Particle beam interactions in plasmas

52.50.−b **Plasma production and heating** *(for Electric discharges, see 52.80.−s)*

52.50.Dg Plasma sources

52.50.Gj Plasma heating by particle beams

52.50.Jm Plasma production and heating by laser beams (laser−foil, laser−cluster, etc.)

52.50.Lp Plasma production and heating by shock waves and compression

52.50.Nr Plasma heating by DC fields; ohmic heating, arcs

52.50.Qt Plasma heating by radio-frequency fields; ICR, ICP, helicons

52.50.Sw Plasma heating by microwaves; ECR, LH, collisional heating

52.55.−s **Magnetic confinement and equilibrium** *(see also 28.52.−s Fusion reactors)*

52.55.Dy General theory and basic studies of plasma lifetime, particle and heat loss, energy balance, field structure, etc.

52.55.Ez Theta pinch

52.55.Fa Tokamaks, spherical tokamaks

52.55.Hc Stellarators, torsatrons, heliacs, bumpy tori, and other toroidal confinement devices

52.55.Ip Spheromaks

52.55.Jd Magnetic mirrors, gas dynamic traps

52.55.Lf Field-reversed configurations, rotamaks, astrons, ion rings, magnetized target fusion, and cusps

52.55.Pi Fusion products effects (e.g., alpha-particles, etc.), fast particle effects

52.55.Rk Power exhaust; divertors

52.55.Tn Ideal and resistive MHD modes; kinetic modes

52.55.Wq Current drive; helicity injection

52.57.−z Laser inertial confinement

52.57.Bc Target design and fabrication

52.57.Fg Implosion symmetry and hydrodynamic instability (Rayleigh-Taylor, Richtmyer-Meshkov, imprint, etc.)

52.57.Kk Fast ignition of compressed fusion fuels

52.58.−c Other confinement methods

52.58.Ei Light-ion inertial confinement

52.58.Hm Heavy-ion inertial confinement

52.58.Lq Z-pinches, plasma focus and other pinch devices

52.58.Qv Electrostatic and high-frequency confinement

52.59.−f Intense particle beams and radiation sources *(see also 29.25.−t and 29.27.−a in instrumentation for particle and nuclear physics)*

52.59.Bi Grid- and ion-diode-accelerated beams

52.59.Dk Magneto-plasma accelerated plasmas

52.59.Fn Multistage accelerated heavy-ion beams

52.59.Hq Dense plasma focus

52.59.Mv High-voltage diodes *(for high-current and high-voltage technology, see 84.70.+p)*

52.59.Px Hard X-ray sources

52.59.Qy Wire array Z-pinches

52.59.Rz Free-electron devices *(for free-electron lasers, see 41.60.Cr)*

52.59.Sa Space-charge-dominated beams

52.59.Tb Moderate-intensity beams

52.59.Wd Emittance-dominated beams

52.59.Ye Plasma devices for generation of coherent radiation

52.65.−y Plasma simulation

52.65.Cc Particle orbit and trajectory

52.65.Ff Fokker-Planck and Vlasov equation

52.65.Kj Magnetohydrodynamic and fluid equation

52.65.Pp Monte Carlo methods

52.65.Rr Particle-in-cell method

52.65.Tt Gyrofluid and gyrokinetic simulations

52.65.Vv Perturbative methods

52.65.Ww Hybrid methods

52.65.Yy Molecular dynamics methods

52.70.−m Plasma diagnostic techniques and instrumentation

52.70.Ds Electric and magnetic measurements

52.70.Gw Radio-frequency and microwave measurements

52.70.Kz Optical (ultraviolet, visible, infrared) measurements

52.70.La X-ray and γ-ray measurements

52.70.Nc Particle measurements

52.72.+v Laboratory studies of space- and astrophysical-plasma processes *(see also 95.30.Qd in fundamental aspects of astrophysics and 94.20.−y and 94.30.−d in aeronomy and magnetospheric physics)*

52.75.−d Plasma devices *(for ion sources, see 29.25.Lg, Ni; for plasma sources, see 52.50.Dg)*

52.75.Di Ion and plasma propulsion

52.75.Fk Magnetohydrodynamic generators and thermionic convertors; plasma diodes *(see also 84.60.Lw, Ny in direct-energy conversion and storage)*

52.75.Hn Plasma torches

52.75.Kq Plasma switches (e.g., spark gaps)

52.75.Xx Thermionic and filament-based sources (e.g., Q machines, double- and triple-plasma devices, etc.)

52.77.−j Plasma applications

52.77.Bn Etching and cleaning *(see also 81.65.Cf Surface cleaning, etching, patterning in surface treatments)*

52.77.Dq Plasma-based ion implantation and deposition *(see also 81.15.Jj Ion and electron beam-assisted deposition)*

52.77.Fv High-pressure, high-current plasmas (plasma spray, arc welding, etc.) *(see also 81.15.Rs Spray coating techniques)*

. . . . *Chemical synthesis; combustion synthesis, see 81.20.Ka*

52.80.−s Electric discharges *(see also 51.50.+v Electrical properties of gases; for plasma reactions including flowing afterglow and electric discharges, see 82.33.Xj in physical chemistry and chemical physics)*

52.80.Dy Low-field and Townsend discharges

52.80.Hc Glow; corona

52.80.Mg Arcs; sparks; lightning; atmospheric electricity *(see also 92.60.Pw in hydrospheric and atomospheric geophysics)*

52.80.Pi High-frequency and RF discharges

52.80.Qj Explosions; exploding wires

52.80.Sm Magnetoactive discharges (e.g., Penning discharges)

52.80.Tn Other gas discharges

52.80.Vp Discharge in vacuum

52.80.Wq Discharge in liquids and solids *(for electric breakdown in liquids, see 77.22.Jp)*

52.80.Yr Discharges for spectral sources (including inductively coupled plasma)

52.90.+z Other topics in physics of plasmas and electric discharges (restricted to new topics in section 52)

60. CONDENSED MATTER: STRUCTURAL, MECHANICAL AND THERMAL PROPERTIES

61. Structure of solids and liquids; crystallography *(for surface, interface, and thin film structure, see section 68)*

61.10.−i **X-ray diffraction and scattering** *(for x-ray diffractometers, see 07.85.Jy; for x-ray studies of crystal defects, see 61.72.Dd, Ff)*

61.10.Dp Theories of diffraction and scattering

61.10.Eq X-ray scattering (including small-angle scattering)

61.10.Ht X-ray absorption spectroscopy: EXAFS, NEXAFS, XANES, etc.

61.10.Kw X-ray reflectometry (surfaces, interfaces, films)

61.10.Nz X-ray diffraction

61.12.−q **Neutron diffraction and scattering**

61.12.Bt Theories of diffraction and scattering

61.12.Ex Neutron scattering (including small-angle scattering)

61.12.Ha Neutron reflectometry

61.12.Ld Neutron diffraction

61.14.−x **Electron diffraction and scattering** *(for electron diffractometers, see 07.78.+s)*

61.14.Dc Theories of diffraction and scattering

61.14.Hg Low-energy electron diffraction (LEED) and reflection high-energy electron diffraction (RHEED)

61.14.Lj Convergent-beam electron diffraction, selected-area electron diffraction, nanodiffraction

61.14.Nm Electron holography

61.14.Qp X-ray photoelectron diffraction

. . . . *Microscopy of surfaces, interfaces, and thin films, see 68.37.−d*

61.18.−j **Other methods of structure determination**

61.18.Bn Atom, molecule, and ion scattering

61.18.Fs Magnetic resonance techniques; Mössbauer spectroscopy

61.20.−p **Structure of liquids**

61.20.Gy Theory and models of liquid structure

61.20.Ja Computer simulation of liquid structure

61.20.Lc Time-dependent properties; relaxation *(for glass transitions, see 64.70.Pf)*

61.20.Ne Structure of simple liquids

61.20.Qg Structure of associated liquids: electrolytes, molten salts, etc.

61.25.−f **Studies of specific liquid structures**

61.25.Bi Liquid noble gases

61.25.Em Molecular liquids

61.25.Hq Macromolecular and polymer solutions; polymer melts; swelling

61.25.Mv Liquid metals and alloys

61.30.−v **Liquid crystals** *(for phase transitions in liquid crystals, see 64.70.Md; for liquid crystals as dielectric materials, see 77.84.Nh; for liquid crystals as optical materials, see 42.70.Df; for liquid crystal devices, see 42.79.Kt)*

61.30.Cz Molecular and microscopic models and theories of liquid crystal structure

61.30.Dk Continuum models and theories of liquid crystal structure

61.30.Eb Experimental determinations of smectic, nematic, cholesteric, and other structures

61.30.Gd Orientational order of liquid crystals; electric and magnetic field effects on order

61.30.Hn Surface phenomena: alignment, anchoring, anchoring transitions, surface-induced layering, surface-induced ordering, wetting, prewetting transitions, and wetting transitions *(see also section 68 Surfaces and interfaces; thin films and low-dimensional systems)*

61.30.Jf Defects in liquid crystals

61.30.Mp Blue phases and other defect-phases

61.30.Pq Microconfined liquid crystals: droplets, cylinders, randomly confined liquid crystals, polymer dispersed liquid crystals, and porous systems

61.30.St Lyotropic phases

61.30.Vx Polymer liquid crystals

61.41.+e **Polymers, elastomers, and plastics** *(see also 81.05.Lg in materials science; for rheology of polymers, see section 83; for polymer reactions and polymenization, see 82.35.−x in physical chemistry and chemical physics)*

61.43.−j **Disordered solids** *(see also 81.05.Gc, 81.05.Kf, and 81.05.Rm in materials science; for photoluminescence of disordered solids, see 78.55.Mb and 78.55.Qr)*

61.43.Bn Structural modeling: serial-addition models, computer simulation

61.43.Dq Amorphous semiconductors, metals, and alloys

61.43.Er Other amorphous solids

61.43.Fs Glasses

61.43.Gt Powders, porous materials

61.43.Hv Fractals; macroscopic aggregates (including diffusion-limited aggregates)

61.44.−n **Semi-periodic solids**

61.44.Br Quasicrystals

61.44.Fw Incommensurate crystals

61.46.+w **Nanoscale materials: clusters, nanoparticles, nanotubes, and nanocrystals** *(see also 36.40.−c Atomic and molecular clusters; for fabrication and characterization of nanoscale materials, see 81.07.−b in materials science)*

61.48.+c **Fullerenes and fullerene-related materials** *(see also 81.05.Tp Fullerenes and related materials in materials science)*

61.50.−f **Crystalline state**

61.50.Ah Theory of crystal structure, crystal symmetry; calculations and modeling

. . . . *Crystal growth, see 81.10.−h*

61.50.Ks Crystallographic aspects of phase transformations; pressure effects *(see also 81.30.Hd in materials science)*

61.50.Lt Crystal binding; cohesive energy

61.50.Nw Crystal stoichiometry

61.66.−f **Structure of specific crystalline solids** *(for surface structure, see 68.35.Bs)*

61.66.Bi Elemental solids

61.66.Dk Alloys

61.66.Fn Inorganic compounds

61.66.Hq Organic compounds

. . . . *Quantum crystals, see 67.80.Cx*

61.68.+n **Crystallographic databases**

61.72.−y **Defects and impurities in crystals; microstructure** *(for radiation induced defects, see 61.80.−x; for defects in surfaces, interfaces and thin films, see 68.35.Dv and 68.55.Ln; see also 85.40.Ry Impurity doping, diffusion and ion implantation technology)*

61.72.Bb Theories and models of crystal defects

61.72.Cc Kinetics of defect formation and annealing

61.72.Dd Experimental determination of defects by diffraction and scattering

61.72.Ff Direct observation of dislocations and other defects (etch pits, decoration, electron microscopy, x-ray topography, etc.)

61.72.Hh Indirect evidence of dislocations and other defects (resistivity, slip, creep, strains, internal friction, EPR, NMR, etc.)

61.72.Ji Point defects (vacancies, interstitials, color centers, etc.) and defect clusters

61.72.Lk Linear defects: dislocations, disclinations

61.72.Mm Grain and twin boundaries

61.72.Nn Stacking faults and other planar or extended defects

61.72.Qq Microscopic defects (voids, inclusions, etc.)

61.72.Ss Impurity concentration, distribution, and gradients *(for impurities in thin films, see 68.55.Ln; see also 66.30.Jt Diffusion of impurities)*

61.72.Tt Doping and impurity implantation in germanium and silicon

61.72.Vv Doping and impurity implantation in III–V and II–VI semiconductors

61.72.Ww Doping and impurity implantation in other materials

61.72.Yx Interaction between different crystal defects; gettering effect

61.80.−x Physical radiation effects, radiation damage (for photochemical reactions, see 82.50.−m)

. . . . *Radiation treatments, see 81.40.Wx*

61.80.Az Theory and models of radiation effects

61.80.Ba Ultraviolet, visible, and infrared radiation effects (including laser radiation)

61.80.Cb X-ray effects

61.80.Ed γ-ray effects

61.80.Fe Electrons and positron radiation effects

61.80.Hg Neutron radiation effects

61.80.Jh Ion radiation effects *(for ion implantation, see 61.72.Tt, Vv, Ww)*

61.80.Lj Atom and molecule irradiation effects

. . . . *Channeling, blocking, and energy loss of particles, see 61.85.+p*

61.82.−d Radiation effects on specific materials

61.82.Bg Metals and alloys

61.82.Fk Semiconductors

61.82.Ms Insulators

61.82.Pv Polymers, organic compounds

61.82.Rx Nanocrystalline materials

61.85.+p Channeling phenomena (blocking, energy loss, etc.)

61.90.+d Other topics in structure of solids and liquids (restricted to new topics in section 61)

62. Mechanical and acoustical properties of condensed matter

(for nonlinear acoustics of solids, see 43.25.Dc—in acoustics appendix; for mechanical and acoustical properties of interfaces and thin films, see 68.35.Gy, 68.35.Iv, and 68.60.Bs; for mechanical properties related to treatment conditions, see 81.40.Jj, Lm, Np—in material science; for mechanical and acoustical properties of superconductors, see 74.25.Ld; for mechanical properties of rocks and minerals, see 91.60.−x)

62.10.+s Mechanical properties of liquids *(for viscosity of liquids, see 66.20.−x)*

62.20.−x Mechanical properties of solids

62.20.Dc Elasticity, elastic constants

62.20.Fe Deformation and plasticity (including yield, ductility, and superplasticity) *(see also 83.50.−v Deformation and flow in rheology)*

62.20.Hg Creep

62.20.Mk Fatigue, brittleness, fracture, and cracks

62.20.Qp Tribology and hardness *(see also 46.55.+d Tribology and mechanical contacts in continuum mechanics of solids)*

62.25.+g Mechanical properties of nanoscale materials

62.30.+d Mechanical and elastic waves; vibrations *(see also 43.40.+s Structural acoustics and vibration; 46.40.−f Vibrations and mechanical waves in continuum mechanics of solids)*

62.40.+i Anelasticity, internal friction, stress relaxation, and mechanical resonances *(see also 81.40.Jj Elasticity and anelasticity)*

. . . . *Thermomechanical effects, see 65.40.De*

. . . . *Magnetomechanical effects, see 75.80.+q*

. . . . *Piezoelectric effects, see 77.65.−j*

. . . . *Elastooptical effects, see 78.20.Hp*

62.50.+p High-pressure and shock wave effects in solids and liquids *(for high pressure apparatus and techniques, see 07.35.+k; for shock wave initiated high-pressure chemistry, see 82.40.Fp)*

62.60.+v Acoustical properties of liquids *(see also 43.35.+d in acoustics)*

. . . . *Lattice dynamics, phonons, see section 63*

. . . . *Second sound in quantum fluids, see 67.40.Pm*

62.65.+k Acoustical properties of solids

. . . . *Magnetoacoustic effects, see 72.55.+s and 73.50.Rb*

. . . . *Acoustoelectric effects, see 72.50.+b, 73.50.Rb, and 77.65.Dq*

. . . . *Acoustooptical effects, see 78.20.Hp*

62.80.+f Ultrasonic relaxation *(see also 43.35.Fj Ultrasonic relaxation processes in liquids and solids—in acoustics appendix; for ultrasonic attenuation in superconductors, see 74.25.Ld)*

62.90.+k Other topics in mechanical and acoustical properties of condensed matter (restricted to new topics in section 62)

63. Lattice dynamics *(see also 78.30.−j Infrared and Raman spectra; for surface and interface vibrations, see 68.35.Ja; for adsorbate vibrations, see 68.43.Pq)*

63.10.+a General theory

63.20.−e Phonons in crystal lattices *(for phonons in superconductors, see 74.25.Kc; see also 43.35.Gk Phonons in crystal lattice, quantum acoustics—in acoustics appendix)*

63.20.Dj Phonon states and bands, normal modes, and phonon dispersion

63.20.Kr Phonon–electron and phonon–phonon interactions

63.20.Ls Phonon interactions with other quasiparticles

63.20.Mt Phonon–defect interactions

63.20.Pw Localized modes

63.20.Ry Anharmonic lattice modes

63.22.+m Phonons or vibrational states in low-dimensional structures and nanoscale materials

63.50.+x Vibrational states in disordered systems

63.70.+h Statistical mechanics of lattice vibrations and displacive phase transitions

63.90.+t Other topics in lattice dynamics (restricted to new topics in section 63)

64. Equations of state, phase equilibria, and phase transitions *(see also 82.60.−s Chemical thermodynamics)*

64.10.+h General theory of equations of state and phase equilibria *(see also 05.70.Ce Thermodynamic functions and equations of state in thermodynamics)*

64.30.+t **Equations of state of specific substances**

64.60.−i **General studies of phase transitions** *(see also 63.70.+h Statistical mechanics of lattice vibrations and displacive phase transitions; for critical phenomena in solid surfaces and interfaces, and in magnetism, see 68.35.Rh, and 75.40.−s, respectively)*

64.60.Ak Renormalization-group, fractal, and percolation studies of phase transitions *(see also 61.43.Hv Fractals; macroscopic aggregates)*

64.60.Cn Order−disorder transformations; statistical mechanics of model systems

64.60.Fr Equilibrium properties near critical points, critical exponents

64.60.Ht Dynamic critical phenomena

64.60.Kw Multicritical points

64.60.My Metastable phases

64.60.Qb Nucleation *(see also 82.60.Nh Thermodynamics of nucleation in physical chemistry and chemical physics)*

64.70.−p **Specific phase transitions**

64.70.Dv Solid−liquid transitions

64.70.Fx Liquid−vapor transitions

64.70.Hz Solid−vapor transitions

64.70.Ja Liquid−liquid transitions

64.70.Kb Solid−solid transitions *(see also 61.50.Ks Crystallographic aspects of phase transformations; pressure effects; 75.30.Kz and 77.80.Bh for magnetic and ferroelectric transitions, respectively; for material science aspects, see 81.30.−t)*

64.70.Md Transitions in liquid crystals

64.70.Nd Structural transitions in nanoscale materials

64.70.Pf Glass transitions

64.70.Rh Commensurate−incommensurate transitions

64.75.+g **Solubility, segregation, and mixing; phase separation** *(see also 82.60.Lf Thermodynamics of solutions)*

64.90.+b **Other topics in equations of state, phase equilibria, and phase transitions (restricted to new topics in section 64)**

65. Thermal properties of condensed matter *(see also 05.70.−a Thermodynamics and section 44 Heat transfer; for thermodynamic properties of quantum fluids and solids, see section 67; for thermal properties of thin films, see 68.60.Dv; for nonelectronic thermal conduction, see 66.60.+a and 66.70.+f; for thermal properties of rocks and minerals, see 91.60.Ki; for thermodynamic properties of superconductors, see 74.25.Bt)*

65.20.+w **Thermal properties of liquids: heat capacity, thermal expansion, etc.**

65.40.−b **Thermal properties of crystalline solids** *(for specific heat of superconductors, see 74.25.Bt; for specific heat of magnetic systems, see 75.40.Cx)*

65.40.Ba Heat capacity

65.40.De Thermal expansion; thermomechanical effects

65.40.Gr Entropy and other thermodynamical quantities

65.60.+a **Thermal properties of amorphous solids and glasses: heat capacity, thermal expansion, etc.**

65.80.+n **Thermal properties of small particles, nanocrystals, nanotubes** *(see also 82.60.Qr Thermodynamics of nanoparticles in physical chemistry and chemical physics)*

65.90.+i **Other topics in thermal properties of condensed matter (restricted to new topics in section 65)**

66. Transport properties of condensed matter (nonelectronic)

66.10.−x **Diffusion and ionic conduction in liquids**

66.10.Cb Diffusion and thermal diffusion *(for osmosis in biological systems, see 82.39.Wj)*

66.10.Ed Ionic conduction

66.20.+d **Viscosity of liquids; diffusive momentum transport**

66.30.−h **Diffusion in solids** *(for surface and interface diffusion, see 68.35.Fx)*

66.30.Dn Theory of diffusion and ionic conduction in solids

66.30.Fq Self-diffusion in metals, semimetals, and alloys

66.30.Hs Self-diffusion and ionic conduction in nonmetals

66.30.Jt Diffusion of impurities

66.30.Lw Diffusion of other defects

66.30.Ny Chemical interdiffusion; diffusion barriers

66.30.Pa Diffusion in nanoscale solids

66.30.Qa Electromigration

66.30.Xj Thermal diffusivity

66.35.+a **Quantum tunneling of defects**

66.60.+a **Thermal conduction in nonmetallic liquids** *(for thermal conduction in liquid metals, see 72.15.Cz)*

66.70.+f **Nonelectronic thermal conduction and heat-pulse propagation in solids; thermal waves** *(for thermal conduction in metals and alloys, see 72.15.Cz and 72.15.Eb)*

66.90.+r **Other topics in nonelectronic transport properties of condensed matter (restricted to new topics in section 66)**

67. Quantum fluids and solids; liquid and solid helium *(see also 05.30.−d Quantum statistical mechanics)*

67.20.+k **Quantum effects on the structure and dynamics of nondegenerate fluids (e.g., normal phase liquid ^{4}He)**

67.40.−w **Boson degeneracy and superfluidity of ^{4}He**

67.40.Bz Phenomenology and two-fluid models

67.40.Db Quantum statistical theory; ground state, elementary excitations

67.40.Fd Dynamics of relaxation phenomena

67.40.Hf Hydrodynamics in specific geometries, flow in narrow channels

67.40.Jg Ions in liquid ^{4}He

67.40.Kh Thermodynamic properties

67.40.Mj First sound

67.40.Pm Transport processes, second and other sounds, and thermal counterflow; Kapitza resistance

67.40.Rp Films and weak link transport

67.40.Vs Vortices and turbulence

67.40.Yv Impurities and other defects

67.55.−s **Normal phase of liquid ^{3}He**

67.55.Cx Thermodynamic properties

67.55.Fa Hydrodynamics

67.55.Hc Transport properties

67.55.Ig Ions in normal liquid ^{3}He

67.55.Jd Collective modes

67.55.Lf Impurities

67.57.−z **Superfluid phase of liquid ^{3}He**

67.57.Bc Thermodynamic properties

67.57.De Superflow and hydrodynamics

67.57.Fg Textures and vortices

67.57.Gh Ions in superfluid ^{3}He

67.57.Hi Transport properties
67.57.Jj Collective modes
67.57.Lm Spin dynamics
67.57.Np Behavior near interfaces
67.57.Pq Impurities

67.60.−g Mixed systems; liquid ^{3}He, ^{4}He mixtures
67.60.Dm He I—^{3}He
67.60.Fp He II—^{3}He
67.60.Hr Dilute superfluid ^{3}He in He II
67.60.Js Ions in liquid ^{3}He—^{4}He mixtures

67.65.+z Spin-polarized hydrogen and helium

67.70.+n Films (including physical adsorption)

67.80.−s Solid helium and related quantum crystals
67.80.Cx Structure, lattice dynamics, and sound propagation
67.80.Gb Thermal properties
67.80.Jd Magnetic properties and nuclear magnetic resonance
67.80.Mg Defects, impurities, and diffusion

67.90.+z Other topics in quantum fluids and solids; liquid and solid helium (restricted to new topics in section 67)

68. Surfaces and interfaces; thin films and low-dimensional systems (structure and nonelectronic properties) *(for surface and interface chemistry, see 82.65.+r, for surface magnetism, see 75.70.Rf)*

68.03.−g Gas-liquid and vacuum-liquid interfaces
68.03.Cd Surface tension and related phenomena
68.03.Fg Evaporation and condensation
68.03.Hj Structure, measurements and simulations
68.03.Kn Dynamics (capillary waves)

68.05.−n Liquid-liquid interfaces
68.05.Cf Structure, measurements and simulations
68.05.Gh Interfacial properties of microemulsions

68.08.−p Liquid-solid interfaces
68.08.Bc Wetting
68.08.De Structure, measurements and simulations
. . . . *Crystal growth, biomineralization, see 81.10.Dn, Fq*

68.15.+e Liquid thin films

68.18.−g Langmuir-Blodgett films on liquids *(for L-B films on solids, see 68.47.Pe)*
68.18.Fg Structure: measurements and simulations
68.18.Jk Phase transitions

68.35.−p Solid surfaces and solid−solid interfaces: Structure and energetics
68.35.Af Atomic scale friction
68.35.Bs Structure of clean surfaces (reconstruction)
68.35.Ct Interface structure and roughness
68.35.Dv Composition, segregation; defects and impurities
68.35.Fx Diffusion; interface formation *(see also 66.30.−h Diffusion in solids, for diffusion of adsorbates, see 68.43.Jk)*
68.35.Gy Mechanical properties; surface strains
68.35.Iv Acoustical properties
68.35.Ja Surface and interface dynamics and vibrations
. . . . *Solid-solid interfaces: transport and optical properties, see 73.40.−c and 78.20.−e respectively*
68.35.Md Surface thermodynamics, surface energies *(see also 05.70.Np Interface and surface thermodynamics in statistical physics, thermodynamics and nonlinear dynamical systems)*
68.35.Np Adhesion *(for polymer adhesion, see 82.35.Gh)*
68.35.Rh Phase transitions and critical phenomena

68.37.−d Microscopy of surfaces, interfaces, and thin films
68.37.Ef Scanning tunneling microscopy (including chemistry induced with STM)
68.37.Hk Scanning electron microscopy (SEM) (including EBIC)
68.37.Lp Transmission electron microscopy (TEM) (including STEM, HRTEM, etc.)
68.37.Nq Low energy electron microscopy (LEEM)
68.37.Ps Atomic force microscopy (AFM)
68.37.Rt Magnetic force microscopy (MFM)
68.37.Tj Acoustic force microscopy
68.37.Uv Near-field scanning microscopy and spectroscopy
68.37.Vj Field emission and field-ion microscopy
68.37.Xy Scanning Auger microscopy, photoelectron microscopy
68.37.Yz X-ray microscopy

68.43.−h Chemisorption/physisorption: adsorbates on surfaces
68.43.Bc Ab initio calculations of adsorbate structure and reactions *(for electronic structure of adsorbates, see 73.20.Hb; for adsorbate reactions, see also 82.65.+r Surface and interface chemistry; heterogeneous catalysis at surfaces)*
68.43.De Statistical mechanics of adsorbates
68.43.Fg Adsorbate structure (binding sites, geometry)
68.43.Hn Structure of assemblies of adsorbates (two- and three-dimensional clustering)
68.43.Jk Diffusion of adsorbates, kinetics of coarsening and aggregation
68.43.Mn Adsorption/desorption kinetics
68.43.Pq Adsorbate vibrations
68.43.Rs Electron stimulated desorption
68.43.Tj Photon stimulated desorption
68.43.Vx Thermal desorption

68.47.−b Solid−gas/vacuum interfaces: types of surfaces
68.47.De Metallic surfaces
68.47.Fg Semiconductor surfaces
68.47.Gh Oxide surfaces
68.47.Jn Clusters on oxide surfaces
68.47.Mn Polymer surfaces
68.47.Pe Langmuir−Blodgett films on solids; polymers on surfaces; biological molecules on surfaces

68.49.−h Surface characterization by particle−surface scattering *(see also 34.50.Dy Interactions of atoms and molecules with surfaces; photon and electron emission; neutralization of ions in atomic and molecular collision processes and interactions)*
68.49.Bc Atom scattering from surfaces (diffraction and energy transfer)
68.49.Df Molecule scattering from surfaces (energy transfer, resonances, trapping)
68.49.Fg Cluster scattering from surfaces
68.49.Jk Electron scattering from surfaces
68.49.Sf Ion scattering from surfaces (charge transfer, sputtering, SIMS)
68.49.Uv X-ray standing waves
. . . . *Surface and interface electron states, see 73.20.−r*
. . . . *Electronic structure of adsorbates, see 73.20.Hb*
. . . . *Vibrational spectroscopy (IR, Raman, ATR), see 78.30.−j*
. . . . *Electron spectroscopy (EELS, Auger, metastable quenching spectroscopy see 79.20.−m*
. . . . *Photoelectron spectroscopy (XPS and UPS), see 79.60.−i*
. . . . *Nonlinear spectroscopy (second harmonic, sum frequency generation, etc.), see 42.65.Ky*
. . . . *Electron diffraction (LEED, RHEED), see 61.14.−x*

· · · · *Surface enhanced spectroscopy, plasmons, see 73.20.Mf*

· · · · *Near-field scanning microscopy and spectroscopy, see 68.37.Uv*

68.55.−a Thin film structure and morphology *(for methods of thin film deposition, film growth and epitaxy, see 81.15.−z)*

68.55.Ac Nucleation and growth: microscopic aspects

68.55.Jk Structure and morphology; thickness; crystalline orientation and texture

68.55.Ln Defects and impurities: doping, implantation, distribution, concentration, etc. *(for diffusion of impurities, see 66.30.Jt)*

68.55.Nq Composition and phase identification

68.60.−p Physical properties of thin films, nonelectronic

68.60.Bs Mechanical and acoustical properties

68.60.Dv Thermal stability; thermal effects

68.60.Wm Other nonelectronic physical properties

68.65.−k Low-dimensional, mesoscopic, and nanoscale systems: structure and nonelectronic properties *(for structure of nanoscale materials, see 61.46.+w; for magnetic properties of interfaces, see 75.70.Cn; for superconducting properties, see 74.78.−w; for optical properties, see 78.67.−n; for transport properties, see 73.63.−b)*

· · · · *Growth of low-dimensional structures, see 81.16.−c*

68.65.Ac Multilayers

68.65.Cd Superlattices

68.65.Fg Quantum wells

68.65.Hb Quantum dots

68.65.La Quantum wires

68.70.+w Whiskers and dendrites (growth, structure, and nonelectronic properties)

68.90.+g Other topics in structure, and nonelectronic properties of surfaces and interfaces; thin films and low-dimensional structures (restricted to new topics in section 68)

70. CONDENSED MATTER: ELECTRONIC STRUCTURE, ELECTRICAL, MAGNETIC, AND OPTICAL PROPERTIES

71. Electronic structure of bulk materials *(see section 73 for electronic structure of surfaces, interfaces, low-dimensional structures, and nanomaterials; for electronic structure of superconductors, see 74.25.Jb)*

71.10.−w Theories and models of many-electron systems
71.10.Ay Fermi-liquid theory and other phenomenological models
71.10.Ca Electron gas, Fermi gas
71.10.Fd Lattice fermion models (Hubbard model, etc.)
71.10.Hf Non-Fermi-liquid ground states, electron phase diagrams and phase transitions in model systems
71.10.Li Excited states and pairing interactions in model systems
71.10.Pm Fermions in reduced dimensions (anyons, composite fermions, Luttinger liquid, etc.) *(for anyon mechanism in superconductors, see 74.20.Mn)*

71.15.−m Methods of electronic structure calculations *(see also 31.15.−p Calculations and mathematical techniques in atomic and molecular physics)*
71.15.Ap Basis sets (LCAO, plane-wave, APW, etc.) and related methodology (scattering methods, ASA, linearized methods, etc.)
71.15.Dx Computational methodology (Brillouin zone sampling, iterative diagonalization, pseudopotential construction)
71.15.Mb Density functional theory, local density approximation, gradient and other corrections
71.15.Nc Total energy and cohesive energy calculations
71.15.Pd Molecular dynamics calculations (Car−Parrinello) and other numerical simulations
71.15.Qe Excited states: methodology *(see also 71.10.Li Excited states and pairing interactions in model systems)*
71.15.Rf Relativistic effects

71.18.+y Fermi surface: calculations and measurements; effective mass, g factor

71.20.−b Electron density of states and band structure of crystalline solids *(for electronic structure of superconductors, see 74.25.Jb)*
71.20.Be Transition metals and alloys
71.20.Dg Alkali and alkaline earth metals
71.20.Eh Rare earth metals and alloys
71.20.Gj Other metals and alloys
71.20.Lp Intermetallic compounds
71.20.Mq Elemental semiconductors
71.20.Nr Semiconductor compounds
71.20.Ps Other inorganic compounds
71.20.Rv Polymers and organic compounds
71.20.Tx Fullerenes and related materials; intercalation compounds
. . . . *Photonic band-gap materials, see 42.70.Qs*

71.22.+i Electronic structure of liquid metals and semiconductors and their alloys

71.23.−k Electronic structure of disordered solids
71.23.An Theories and models; localized states
71.23.Cq Amorphous semiconductors, metallic glasses, glasses
71.23.Ft Quasicrystals

71.27.+a Strongly correlated electron systems; heavy fermions

71.28.+d Narrow-band systems; intermediate-valence solids *(for magnetic aspects, see 75.20.Hr and 75.30.Mb in magnetic properties and materials)*

71.30.+h Metal−insulator transitions and other electronic transitions

71.35.−y Excitons and related phenomena
71.35.Aa Frenkel excitons and self-trapped excitons
71.35.Cc Intrinsic properties of excitons; optical absorption spectra
71.35.Ee Electron-hole drops and electron-hole plasma
71.35.Gg Exciton-mediated interactions
71.35.Ji Excitons in magnetic fields; magnetoexcitons
71.35.Lk Collective effects (Bose effects, phase space filling, and excitonic phase transitions)
71.35.Pq Charged excitons (trions)

71.36.+c Polaritons (including photon−phonon and photon−magnon interactions)

71.38.−k Polarons and electron-phonon interactions *(see also 63.20.Kr Phonon-electron interactions in lattices)*
71.38.Cn Mass renormalization in metals
71.38.Fp Large or Fröhlich polarons
71.38.Ht Self-trapped or small polarons
71.38.Mx Bipolarons

71.45.−d Collective effects
71.45.Gm Exchange, correlation, dielectric and magnetic response functions, plasmons
71.45.Lr Charge-density-wave systems *(see also 75.30.Fv Spin-density waves)*

71.55.−i Impurity and defect levels
71.55.Ak Metals, semimetals, and alloys
71.55.Cn Elemental semiconductors
71.55.Eq III−V semiconductors
71.55.Gs II−VI semiconductors
71.55.Ht Other nonmetals
71.55.Jv Disordered structures; amorphous and glassy solids

71.60.+z Positron states *(for positron annihilation, see 78.70.Bj)*

71.70.−d Level splitting and interactions *(see also 73.20.−r Surface and interface electron states; 75.30.Et Exchange and superexchange interactions)*
71.70.Ch Crystal and ligand fields
71.70.Di Landau levels
71.70.Ej Spin−orbit coupling, Zeeman and Stark splitting, Jahn−Teller effect
71.70.Fk Strain-induced splitting
71.70.Gm Exchange interactions
71.70.Jp Nuclear states and interactions

71.90.+q Other topics in electronic structure (restricted to new topics in section 71)

72. Electronic transport in condensed matter *(for electronic transport in surfaces, interfaces, and thin films, see section 73; for electrical properties related to treatment conditions, see 81.40.Rs; for transport properties of superconductors, see 74.25.Fy)*

72.10.−d Theory of electronic transport; scattering mechanisms
72.10.Bg General formulation of transport theory
72.10.Di Scattering by phonons, magnons, and other nonlocalized excitations *(see also 71.45.−d Collective effects in electronic structure of bulk materials)*
72.10.Fk Scattering by point defects, dislocations, surfaces, and other imperfections (including Kondo effect)

72.15.−v Electronic conduction in metals and alloys

72.15.Cz Electrical and thermal conduction in amorphous and liquid metals and alloys
72.15.Eb Electrical and thermal conduction in crystalline metals and alloys
72.15.Gd Galvanomagnetic and other magnetotransport effects *(see also 75.47.−m Magnetotransport phenomena; materials for magnetotransport)*
72.15.Jf Thermoelectric and thermomagnetic effects
72.15.Lh Relaxation times and mean free paths
72.15.Nj Collective modes (e.g., in one-dimensional conductors)
72.15.Qm Scattering mechanisms and Kondo effect *(see also 75.20.Hr Local moments in compounds and alloys; Kondo effect, valence fluctuations, heavy fermions in magnetic properties and materials)*
72.15.Rn Localization effects (Anderson or weak localization)

72.20.−i Conductivity phenomena in semiconductors and insulators *(see also 66.70.+f Nonelectronic thermal conduction in solids)*
72.20.Dp General theory, scattering mechanisms
72.20.Ee Mobility edges; hopping transport
72.20.Fr Low-field transport and mobility; piezoresistance
72.20.Ht High-field and nonlinear effects
72.20.Jv Charge carriers: generation, recombination, lifetime, and trapping
72.20.My Galvanomagnetic and other magnetotransport effects
72.20.Pa Thermoelectric and thermomagnetic effects

72.25.−b Spin polarized transport *(for ballistic magnetoresistance, see 75.47.Jn; for spin polarized transport devices, see 85.75.−d)*
72.25.Ba Spin polarized transport in metals
72.25.Dc Spin polarized transport in semiconductors
72.25.Fe Optical creation of spin polarized carriers
72.25.Hg Electrical injection of spin polarized carriers
72.25.Mk Spin transport through interfaces
72.25.Pn Current-driven spin pumping
72.25.Rb Spin relaxation and scattering

72.30.+q High-frequency effects; plasma effects

72.40.+w Photoconduction and photovoltaic effects

72.50.+b Acoustoelectric effects

72.55.+s Magnetoacoustic effects *(see also 75.80.+q Magnetomechanical and magnetoelectric effects, magnetostriction)*

72.60.+g Mixed conductivity and conductivity transitions

72.70.+m Noise processes and phenomena

72.80.−r Conductivity of specific materials *(for conductivity of metals and alloys, see 72.15.−v)*
72.80.Cw Elemental semiconductors
72.80.Ey III–V and II–VI semiconductors
72.80.Ga Transition-metal compounds
72.80.Jc Other crystalline inorganic semiconductors
72.80.Le Polymers; organic compounds (including organic semiconductors)
72.80.Ng Disordered solids
72.80.Ph Liquid semiconductors
72.80.Rj Fullerenes and related materials
72.80.Sk Insulators
72.80.Tm Composite materials

72.90.+y Other topics in electronic transport in condensed matter (restricted to new topics in section 72)

73. Electronic structure and electrical properties of surfaces, interfaces, thin films, and low-dimensional structures *(for electronic structure and electrical properties of superconducting films and low-dimensional structures, see 74.78.−w; for computational methodology for electronic structure calculations in condensed matter, see 71.15.−m)*

73.20.−r Electron states at surfaces and interfaces
73.20.At Surface states, band structure, electron density of states
73.20.Fz Weak or Anderson localization
73.20.Hb Impurity and defect levels; energy states of adsorbed species
73.20.Jc Delocalization processes
73.20.Mf Collective excitations (including excitons, polarons, plasmons and other charge-density excitations) *(for collective excitations in quantum Hall effects, see 73.43.Lp)*
73.20.Qt Electron solids

73.21.−b Electron states and collective excitations in multilayers, quantum wells, mesoscopic, and nanoscale systems *(for electron states in nanoscale materials, see 73.22.−f)*
73.21.Ac Multilayers
73.21.Cd Superlattices
73.21.Fg Quantum wells
73.21.Hb Quantum wires
73.21.La Quantum dots

73.22.−f Electronic structure of nanoscale materials: clusters, nanoparticles, nanotubes, and nanocrystals
73.22.Dj Single particle states
73.22.Gk Broken symmetry phases
73.22.Lp Collective excitations

73.23.−b Electronic transport in mesoscopic systems
73.23.Ad Ballistic transport *(see also 75.47.Jn Ballistic magnetoresistance in magnetic properties and materials)*
73.23.Hk Coulomb blockade; single-electron tunneling
73.23.Ra Persistent currents

73.25.+i Surface conductivity and carrier phenomena

73.30.+y Surface double layers, Schottky barriers, and work functions *(see also 82.45.Mp Thin layers, films, monolayers, membranes in electrochemistry)*

73.40.−c Electronic transport in interface structures
73.40.Cg Contact resistance, contact potential
73.40.Ei Rectification
73.40.Gk Tunneling *(for tunneling in quantum Hall effects, see 73.43.Jn)*
73.40.Jn Metal-to-metal contacts
73.40.Kp III–V semiconductor-to-semiconductor contacts, *p–n* junctions, and heterojunctions
73.40.Lq Other semiconductor-to-semiconductor contacts, *p–n* junctions, and heterojunctions
73.40.Mr Semiconductor–electrolyte contacts
73.40.Ns Metal–nonmetal contacts
73.40.Qv Metal–insulator–semiconductor structures (including semiconductor-to-insulator)
73.40.Rw Metal–insulator–metal structures
73.40.Sx Metal–semiconductor–metal structures
73.40.Ty Semiconductor–insulator–semiconductor structures
73.40.Vz Semiconductor–metal–semiconductor structures

73.43.−f Quantum Hall effects
73.43.Cd Theory and modeling
73.43.Fj Novel experimental methods; measurements
73.43.Jn Tunneling
73.43.Lp Collective excitations
73.43.Nq Quantum phase transitions

73.43.Qt Magnetoresistance *(see also 75.47.−m Magnetotransport phenomena; materials for magnetotransport in magnetic properties and materials)*

. . . . *Optical properties, see 78.66.−w*

73.50.−h Electronic transport phenomena in thin films *(for electronic transport in mesoscopic systems, see 73.23.−b; see also 73.40.−c Electronic transport in interface structures; for electronic transport in nanoscale materials and structures, see 73.63.−b)*

73.50.Bk General theory, scattering mechanisms

73.50.Dn Low-field transport and mobility; piezoresistance

73.50.Fq High-field and nonlinear effects

73.50.Gr Charge carriers: generation, recombination, lifetime, trapping, mean free paths

73.50.Jt Galvanomagnetic and other magnetotransport effects (including thermomagnetic effects)

73.50.Lw Thermoelectric effects

73.50.Mx High-frequency effects; plasma effects

73.50.Pz Photoconduction and photovoltaic effects

73.50.Rb Acoustoelectric and magnetoacoustic effects

73.50.Td Noise processes and phenomena

73.61.−r Electrical properties of specific thin films *(for optical properties of thin films, see 78.20.−e and 78.66.−w; for magnetic properties of thin films, see 75.70.−i)*

73.61.At Metal and metallic alloys

73.61.Cw Elemental semiconductors

73.61.Ey III−V semiconductors

73.61.Ga II−VI semiconductors

73.61.Jc Amorphous semiconductors; glasses

73.61.Le Other inorganic semiconductors

73.61.Ng Insulators

73.61.Ph Polymers; organic compounds

73.61.Wp Fullerenes and related materials

73.63.−b Electronic transport in nanoscale materials and structures *(see also 73.23.−b Electronic transport in mesoscopic systems)*

73.63.Bd Nanocrystalline materials

73.63.Fg Nanotubes

73.63.Hs Quantum wells

73.63.Kv Quantum dots

73.63.Nm Quantum wires

73.63.Rt Nanoscale contacts

73.90.+f Other topics in electronic structure and electrical properties of surfaces, interfaces, thin films, and low-dimensional structures (Restricted to new topics in section 73)

74. Superconductivity *(for superconducting devices, see 85.25.−j)*

74.10.+v Occurrence, potential candidates

74.20.−z Theories and models of superconducting state

74.20.De Phenomenological theories (two-fluid, Ginzburg–Landau, etc.)

74.20.Fg BCS theory and its development

74.20.Mn Nonconventional mechanisms (spin fluctuations, polarons and bipolarons, resonating valence bond model, anyon mechanism, marginal Fermi liquid, Luttinger liquid, etc.)

74.20.Rp Pairing symmetries (other than s-wave)

74.25.−q Properties of type I and type II superconductors

74.25.Bt Thermodynamic properties

74.25.Dw Superconductivity phase diagrams

74.25.Fy Transport properties (electric and thermal conductivity, thermoelectric effects, etc.)

74.25.Gz Optical properties

74.25.Ha Magnetic properties

74.25.Jb Electronic structure

74.25.Kc Phonons

74.25.Ld Mechanical and acoustical properties, elasticity, and ultrasonic attenuation

74.25.Nf Response to electromagnetic fields (nuclear magnetic resonance, surface impedance, etc.)

74.25.Op Mixed states, critical fields, and surface sheaths

74.25.Qt Vortex lattices, flux pinning, flux creep

74.25.Sv Critical currents

74.40.+k Fluctuations (noise, chaos, nonequilibrium superconductivity, localization, etc.)

74.45.+c Proximity effects; Andreev effect; SN and SNS junctions

74.50.+r Tunneling phenomena; point contacts, weak links, Josephson effects *(for SQUIDs, see 85.25.Dq; for Josephson devices, see 85.25.Cp; for Josephson junction arrays, see 74.81.Fa)*

74.62.−c Transition temperature variations

74.62.Bf Effects of material synthesis, crystal structure, and chemical composition

74.62.Dh Effects of crystal defects, doping and substitution

74.62.Fj Pressure effects

74.62.Yb Other effects

74.70.−b Superconducting materials *(for cuprates see 74.72.−h)*

74.70.Ad Metals; alloys and binary compounds (including A15, MgB_2, etc.)

74.70.Dd Ternary, quaternary and multinary compounds (including Chevrel phases, borocarbides, etc.)

74.70.Kn Organic superconductors

74.70.Pq Ruthenates

74.70.Tx Heavy-fermion superconductors

74.70.Wz Fullerenes and related materials

74.72.−h Cuprate superconductors (high-T_c and insulating parent compounds)

74.72.Bk Y-based cuprates

74.72.Dn La-based cuprates

74.72.Hs Bi-based cuprates

74.72.Jt Other cuprates, including Tl and Hg-based cuprates

74.78.−w Superconducting films and low-dimensional structures

74.78.Bz High-T_c films

74.78.Db Low-T_c films

74.78.Fk Multilayers, superlattices, heterostructures

74.78.Na Mesoscopic and nanoscale systems

74.81.−g Inhomogeneous superconductors and superconducting systems

74.81.Bd Granular, melt-textured, amorphous and composite superconductors

74.81.Fa Josephson junction arrays and wire networks

74.90.+n Other topics in superconductivity (restricted to new topics in section 74)

75. Magnetic properties and materials *(for magnetic properties related to treatment conditions, see 81.40.Rs; for magnetic properties of superconductors, see 74.25.Ha; for magnetic properties of rocks and minerals, see 91.60.Pn)*

75.10.−b General theory and models of magnetic ordering *(see also 05.50.+q Lattice theory and statistics)*

75.10.Dg Crystal-field theory and spin Hamiltonians

75.10.Hk Classical spin models

75.10.Jm Quantized spin models

75.10.Lp Band and itinerant models

75.10.Nr Spin-glass and other random models

75.10.Pq Spin chain models

75.20.−g Diamagnetism, paramagnetism, and superparamagnetism

75.20.Ck Nonmetals

75.20.En Metals and alloys

75.20.Hr Local moment in compounds and alloys; Kondo effect, valence fluctuations, heavy fermions *(see also 72.15.Qm Scattering mechanisms and Kondo effect in electronic conduction of metals and alloys)*

75.25.+z Spin arrangements in magnetically ordered materials (including neutron and spin-polarized electron studies, synchrotron-source x-ray scattering, etc.) *(for devices exploiting spin polarized transport, see 85.75.−d)*

75.30.−m Intrinsic properties of magnetically ordered materials *(for critical point effects, see 75.40.−s)*

75.30.Cr Saturation moments and magnetic susceptibilities

75.30.Ds Spin waves *(for spin-wave resonance, see 76.50.+g)*

75.30.Et Exchange and superexchange interactions *(see also 71.70.−d Level splitting and interactions)*

75.30.Fv Spin-density waves

75.30.Gw Magnetic anisotropy

75.30.Hx Magnetic impurity interactions

75.30.Kz Magnetic phase boundaries (including magnetic transitions, metamagnetism, etc.)

75.30.Mb Valence fluctuation, Kondo lattice, and heavy-fermion phenomena *(see also 71.27.−a Strongly correlated electron systems, heavy fermions)*

75.30.Sg Magnetocaloric effect, magnetic cooling

75.30.Wx Spin crossover

75.40.−s Critical-point effects, specific heats, short-range order *(see also 65.40.−b Heat capacities of solids)*

75.40.Cx Static properties (order parameter, static susceptibility, heat capacities, critical exponents, etc.)

75.40.Gb Dynamic properties (dynamic susceptibility, spin waves, spin diffusion, dynamic scaling, etc.)

75.40.Mg Numerical simulation studies

75.45.+j Macroscopic quantum phenomena in magnetic systems

75.47.−m Magnetotransport phenomena; materials for magnetotransport *(for spintronics, see 85.75.−d; see also 72.15.Gd, 73.50.Jt, 73.43.Qt, and 72.25.−b in transport phenomena)*

75.47.De Giant magnetoresistance

75.47.Gk Colossal magnetoresistance

75.47.Jn Ballistic magnetoresistance

75.47.Lx Manganites

75.47.Np Metals and alloys

75.47.Pq Other materials

75.50.−y Studies of specific magnetic materials

75.50.Bb Fe and its alloys

75.50.Cc Other ferromagnetic metals and alloys

75.50.Dd Nonmetallic ferromagnetic materials

75.50.Ee Antiferromagnetics

75.50.Gg Ferrimagnetics

75.50.Kj Amorphous and quasicrystalline magnetic materials

75.50.Lk Spin glasses and other random magnets

75.50.Mm Magnetic liquids

75.50.Pp Magnetic semiconductors

75.50.Ss Magnetic recording materials *(see also 85.70.−w Magnetic devices)*

75.50.Tt Fine-particle systems; nanocrystalline materials

75.50.Vv High coercivity materials

75.50.Ww Permanent magnets

75.50.Xx Molecular magnets

75.60.−d Domain effects, magnetization curves, and hysteresis

75.60.Ch Domain walls and domain structure *(for magnetic bubbles, see 75.70.Kw)*

75.60.Ej Magnetization curves, hysteresis, Barkhausen and related effects

75.60.Jk Magnetization reversal mechanisms

75.60.Lr Magnetic aftereffects

75.60.Nt Magnetic annealing and temperature–hysteresis effects

75.70.−i Magnetic properties of thin films, surfaces, and interfaces *(for magnetic properties of nanostructures, see 75.75.+a)*

75.70.Ak Magnetic properties of monolayers and thin films

75.70.Cn Magnetic properties of interfaces (multilayers, superlattices, heterostructures)

75.70.Kw Domain structure (including magnetic bubbles)

75.70.Rf Surface magnetism

75.75.+a Magnetic properties of nanostructures

75.80.+q Magnetomechanical and magnetoelectric effects, magnetostriction

. . . . *Galvanomagnetic effects, see 72.15.Gd and 72.20.My*

. . . . *Magnetooptical effects, see 78.20.Ls*

75.90.+w Other topics in magnetic properties and materials (restricted to new topics in section 75)

76. Magnetic resonances and relaxations in condensed matter, Mössbauer effect

76.20.+q General theory of resonances and relaxations

76.30.−v Electron paramagnetic resonance and relaxation *(see also 33.35.+r Electron resonance and relaxation in atomic and molecular physics)*

76.30.Da Ions and impurities: general

76.30.Fc Iron group (3*d*) ions and impurities (Ti–Cu)

76.30.He Platinum and palladium group (4*d* and 5*d*) ions and impurities (Zr–Ag and Hf–Au)

76.30.Kg Rare-earth ions and impurities

76.30.Lh Other ions and impurities

76.30.Mi Color centers and other defects

76.30.Pk Conduction electrons

76.30.Rn Free radicals

76.40.+b Diamagnetic and cyclotron resonances

76.50.+g Ferromagnetic, antiferromagnetic, and ferrimagnetic resonances; spin-wave resonance *(see also 75.30.Ds Spin waves)*

76.60.−k Nuclear magnetic resonance and relaxation *(see also 33.25.+k Nuclear resonance and relaxation in atomic and molecular physics and 82.56.−b Nuclear magnetic resonance in physical chemistry and chemical physics)*

76.60.Cq Chemical and Knight shifts

76.60.Es Relaxation effects

76.60.Gv Quadrupole resonance

76.60.Jx Effects of internal magnetic fields

76.60.Lz Spin echoes

76.60.Pc NMR imaging *(for medical NMR imaging, see 87.61.−c)*

76.70.−r Magnetic double resonances and cross effects *(see also 33.40.+f Multiple resonances in atomic and molecular physics)*

76.70.Dx Electron–nuclear double resonance (ENDOR), electron double resonance (ELDOR)

76.70.Fz Double nuclear magnetic resonance (DNMR), dynamical nuclear polarization

76.70.Hb Optically detected magnetic resonance (ODMR)

76.75.+i Muon spin rotation and relaxation

76.80.+y Mo?ssbauer effect; other γ-ray

spectroscopy *(see also 33.45.+x Mo?ssbauer spectra—in atomic and molecular physics)*

. . . . *Magnetic resonance spectrometers, 07.57.Pt*

76.90.+d Other topics in magnetic resonances and relaxations (restricted to new topics in section 76)

77. Dielectrics, piezoelectrics, and ferroelectrics and their properties *(for conductivity phenomena, see 72.20.−i and 72.80.−r; for dielectric properties related to treatment conditions, see 81.40.Tv)*

77.22.−d Dielectric properties of solids and liquids

77.22.Ch Permittivity (dielectric function)

77.22.Ej Polarization and depolarization

77.22.Gm Dielectric loss and relaxation

77.22.Jp Dielectric breakdown and space-charge effects

77.55.+f Dielectric thin films

77.65.−j Piezoelectricity and electromechanical effects

77.65.Bn Piezoelectric and electrostrictive constants

77.65.Dq Acoustoelectric effects and surface acoustic waves (SAW) in piezoelectrics *(see also 43.35.Pt Surface waves in solids and liquids—in acoustics appendix; for surface acoustic wave transducers, see 43.38.Rh—in acoustics appendix)*

77.65.Fs Electromechanical resonance; quartz resonators

77.65.Ly Strain-induced piezoelectric fields

77.70.+a Pyroelectric and electrocaloric effects

77.80.−e Ferroelectricity and antiferroelectricity

77.80.Bh Phase transitions and Curie point

77.80.Dj Domain structure; hysteresis

77.80.Fm Switching phenomena

77.84.−s Dielectric, piezoelectric, ferroelectric, and antiferroelectric materials *(for nonlinear optical materials, see 42.70.Mp; for dielectric materials in electrochemistry, see 82.45.Un)*

77.84.Bw Elements, oxides, nitrides, borides, carbides, chalcogenides, etc.

77.84.Dy Niobates, titanates, tantalates, PZT ceramics, etc.

77.84.Fa KDP- and TGS-type crystals

77.84.Jd Polymers; organic compounds

77.84.Lf Composite materials

77.84.Nh Liquids, emulsions, and suspensions; liquid crystals *(for structure of liquid crystals, see 61.30.−v)*

77.90.+k Other topics in dielectrics, piezoelectrics, and ferroelectrics and their properties (restricted to new topics in section 77)

78. Optical properties, condensed-matter spectroscopy and other interactions of radiation and particles with condensed matter

78.20.−e Optical properties of bulk materials and thin films *(for optical properties related to materials treatment, see 81.40.Tv; for optical materials, see 42.70-a; for optical properties of superconductors, see 74.25.Gs; for optical properties of rocks and minerals, see 91.60.Mk)*

78.20.Bh Theory, models, and numerical simulation

78.20.Ci Optical constants (including refractive index, complex dielectric constant, absorption, reflection and transmission coefficients, emissivity)

78.20.Ek Optical activity

78.20.Fm Birefringence

78.20.Hp Piezo-, elasto-, and acoustooptical effects; photoacoustic effects

78.20.Jq Electrooptical effects

78.20.Ls Magnetooptical effects

78.20.Nv Thermooptical and photothermal effects

. . . . *Nonlinear optical properties, see 42.65.−k*

78.30.−j Infrared and Raman spectra *(for vibrational states in crystals and disordered systems, see 63.20.−e and 63.50.+x respectively)*

78.30.Am Elemental semiconductors and insulators

78.30.Cp Liquids

78.30.Er Solid metals and alloys

78.30.Fs III−V and II−VI semiconductors

78.30.Hv Other nonmetallic inorganics

78.30.Jw Organic compounds, polymers

78.30.Ly Disordered solids

78.30.Na Fullerenes and related materials

78.35.+c Brillouin and Rayleigh scattering; other light scattering *(for Raman scattering, see 78.30.−j)*

78.40.−q Absorption and reflection spectra: visible and ultraviolet *(for infrared spectra, see 78.30.−j)*

78.40.Dw Liquids

78.40.Fy Semiconductors

78.40.Ha Other nonmetallic inorganics

78.40.Kc Metals, semimetals, and alloys

78.40.Me Organic compounds and polymers

78.40.Pg Disordered solids

78.40.Ri Fullerenes and related materials

78.45.+h Stimulated emission *(see also 42.55.−f Lasers)*

78.47.+p Time-resolved optical spectroscopies and other ultrafast optical measurements in condensed matter *(see also 42.65.Re—in nonlinear optics; 82.53.−k Femtochemistry in physical chemistry and chemical physics)*

. . . . *Impurity and defect absorption in solids, see 78.30.−j and 78.40.−q*

78.55.−m Photoluminescence, properties and materials

78.55.Ap Elemental semiconductors

78.55.Bq Liquids

78.55.Cr III−V semiconductors

78.55.Et II−VI semiconductors

78.55.Fv Solid alkali halides

78.55.Hx Other solid inorganic materials

78.55.Kz Solid organic materials

78.55.Mb Porous materials

78.55.Qr Amorphous materials; glasses and other disordered solids

78.60.−b Other luminescence and radiative recombination

78.60.Fi Electroluminescence

78.60.Hk Cathodoluminescence, ionoluminescence

78.60.Kn Thermoluminescence

78.60.Mq Sonoluminescence, triboluminescence

78.60.Ps Chemiluminescence *(see also 42.55.Ks Chemical lasers)*

78.66.−w Optical properties of specific thin films *(for optical properties of low-dimensional, mesoscopic, and nanoscale materials, see 78.67.−n; for optical properties of surfaces, see 78.68.+m)*

78.66.Bz Metals and metallic alloys

78.66.Db Elemental semiconductors and insulators

78.66.Fd III−V semiconductors

78.66.Hf II−VI semiconductors

78.66.Jg Amorphous semiconductors; glasses

78.66.Li Other semiconductors

78.66.Nk Insulators

78.66.Qn Polymers; organic compounds

78.66.Sq Composite materials

78.66.Tr Fullerenes and related materials

78.66.Vs Fine-particle systems

78.67.−n Optical properties of low-dimensional, mesoscopic, and nanoscale materials and structures

78.67.Bf Nanocrystals and nanoparticles

78.67.Ch Nanotubes

78.67.De Quantum wells

78.67.Hc Quantum dots

78.67.Lt Quantum wires

78.67.Pt Multilayers; superlattices

78.68.+m Optical properties of surfaces

78.70.−g Interactions of particles and radiation with matter

78.70.Bj Positron annihilation *(for positron states, see 71.60.+z in electronic structure of bulk materials; for positronium chemistry, see 82.30.Gg in physical chemistry and chemical physics)*

78.70.Ck X-ray scattering

78.70.Dm X-ray absorption spectra

78.70.En X-ray emission spectra and fluorescence

78.70.Gq Microwave and radio-frequency interactions

78.70.Nx Neutron inelastic scattering

78.90.+t Other topics in optical properties, condensed matter spectroscopy and other interactions of particles and radiation with condensed matter (restricted to new topics in section 78)

79. Electron and ion emission by liquids and solids; impact phenomena

79.20.−m Impact phenomena (including electron spectra and sputtering)

79.20.Ap Theory of impact phenomena; numerical simulation

79.20.Ds Laser-beam impact phenomena

79.20.Fv Electron impact: Auger emission

79.20.Hx Electron impact: secondary emission

79.20.Kz Other electron-impact emission phenomena

79.20.La Photon- and electron-stimulated desorption

79.20.Mb Positron emission

79.20.Rf Atomic, molecular, and ion beam impact and interactions with surfaces

. . . . *Electron and ion channeling, see 61.85.+p*

79.20.Uv Electron energy loss spectroscopy *(see also 82.80.Pv Electron spectroscopy in physical chemistry and chemical physics; 34.80.−i Electron scattering in atomic and molecular physics)*

79.40.+z Thermionic emission

79.60.−i Photoemission and photoelectron spectra

79.60.Bm Clean metal, semiconductor, and insulator surfaces

79.60.Dp Adsorbed layers and thin films

79.60.Fr Polymers; organic compounds

79.60.Ht Disordered structures

79.60.Jv Interfaces; heterostructures; nanostructures

79.70.+q Field emission, ionization, evaporation, and desorption

79.75.+g Exoelectron emission

79.90.+b Other topics in electron and ion emission by liquids and solids and impact phenomena (restricted to new topics in section 79)

80. INTERDISCIPLINARY PHYSICS AND RELATED AREAS OF SCIENCE AND TECHNOLOGY

81. Materials science

81.05.−t Specific materials: fabrication, treatment, testing and analysis

. . . . *Superconducting materials, see 74.70.−b and 74.72.−h*

. . . . *Magnetic materials, see 75.50.−y*

. . . . *Optical materials, see 42.70.−a*

. . . . *Dielectric, piezoelectric, and ferroelectric materials, see 77.84.−s*

. . . . *Colloids, gels, and emulsions, see 82.70.Dd, Gg, Kj respectively*

. . . . *Biological materials, see 87.14.−g*

. . . . *Molecular sieves, zeolites, and other complex materials, see 82.75.−z*

81.05.Bx Metals, semimetals, and alloys

81.05.Cy Elemental semiconductors *(for semiconductors in electrochemistry, see 82.45.Vp)*

81.05.Dz II–VI semiconductors

81.05.Ea III–V semiconductors

81.05.Gc Amorphous semiconductors

81.05.Hd Other semiconductors

81.05.Je Ceramics and refractories (including borides, carbides, hydrides, nitrides, oxides, and silicides) *(for ceramics in electrochemistry, see 82.45.Yz)*

81.05.Kf Glasses (including metallic glasses)

81.05.Lg Polymers and plastics; rubber; synthetic and natural fibers; organometallic and organic materials *(for polymers and organic materials in electrochemistry, see 82.45.Wx)*

81.05.Mh Cermets, ceramic and refractory composites

81.05.Ni Dispersion-, fiber-, and platelet-reinforced metal-based composites

81.05.Pj Glass-based composites, vitroceramics

81.05.Qk Reinforced polymers and polymer-based composites

81.05.Rm Porous materials; granular materials *(for granular superconductors, see 74.81.Bd)*

81.05.Tp Fullerenes and related materials

81.05.Uw Carbon, diamond, graphite

81.05.Zx New materials: theory, design, and fabrication

81.07.−b Nanoscale materials and structures: fabrication and characterization *(for nanostructured materials in electrochemistry, see 82.45.Yz; for nanoparticles in polymers, see 82.35.Np in physical chemistry and chemical physics)*

81.07.Bc Nanocrystalline materials

81.07.De Nanotubes

81.07.Lk Nanocontacts

81.07.Nb Molecular nanostructures

81.07.Pr Organic-inorganic hybrid nanostructures

81.07.St Quantum wells

81.07.Ta Quantum dots

81.07.Vb Quantum wires

81.07.Wx Nanopowders

81.10.−h Methods of crystal growth; physics of crystal growth *(for crystal structure, see section 61)*

81.10.Aj Theory and models of crystal growth; physics of crystal growth, crystal morphology and orientation

81.10.Bk Growth from vapor

81.10.Dn Growth from solutions

81.10.Fq Growth from melts; zone melting and refining

81.10.Jt Growth from solid phases (including multiphase diffusion and recrystallization)

81.10.Mx Growth in microgravity environments

81.15.−z Methods of deposition of films and coatings; film growth and epitaxy *(for structure of thin films, see 68.55.−a; see also 85.40.Sz Deposition technology in microelectronics)*

81.15.Aa Theory and models of film growth

81.15.Cd Deposition by sputtering

81.15.Ef Vacuum deposition

81.15.Fg Laser deposition

81.15.Gh Chemical vapor deposition (including plasma-enhanced CVD, MOCVD, etc.) *(for chemistry of MOCVD, see 82.33.Ya in physical chemistry and chemical physics)*

81.15.Hi Molecular, atomic, ion, and chemical beam epitaxy

81.15.Jj Ion and electron beam-assisted deposition; ion plating *(see also 52.77.Dq Plasma-based ion implantation and deposition in physics of plasmas)*

81.15.Kk Vapor phase epitaxy; growth from vapor phase

81.15.Lm Liquid phase epitaxy; deposition from liquid phases (melts, solutions, and surface layers on liquids)

81.15.Np Solid phase epitaxy; growth from solid phases

81.15.Pq Electrodeposition, electroplating

81.15.Rs Spray coating techniques

81.16.−c Methods of nanofabrication and processing *(for femtosecond probing of semiconductor nanostructures, see 82.53.Mj in physical chemistry and chemical physics)*

81.16.Be Chemical synthesis methods *(for electrochemical synthesis, see 82.45.Aa)*

81.16.Dn Self-assembly

81.16.Fg Supramolecular and biochemical assembly

81.16.Hc Catalytic methods

81.16.Mk Laser-assisted deposition

81.16.Nd Nanolithography

81.16.Pr Nanooxidation *(see also 82.37.Np Single molecule reaction kinetics in physical chemistry and chemical physics)*

81.16.Rf Nanoscale pattern formation

81.16.Ta Atom manipulation *(see also 82.37.Gk STM and AFM manipulation of a single molecule in physical chemistry and chemical physics; 39.25.+k Atom manipulation in atomic and molecular physics)*

81.20.−n Methods of materials synthesis and materials processing *(for ion implantation and doping, see 61.72.Tt, Vv, and Ww)*

. . . . *Crystal growth, see 81.10.−h*

. . . . *Film deposition, film growth and epitaxy, see 81.15.−z*

81.20.Ev Powder processing: powder metallurgy, compaction, sintering, mechanical alloying, and granulation

81.20.Fw Sol–gel processing, precipitation

81.20.Hy Forming; molding, extrusion etc.

81.20.Ka Chemical synthesis; combustion synthesis *(for electrochemical synthesis, see 82.45.Aa)*

. . . . *Chemical vapor deposition, see 81.15.Gh*

81.20.Rg Aerosols in materials synthesis and processing

81.20.Vj Joining; welding

81.20.Wk Machining, milling

81.20.Ym Purification

81.30.−t Phase diagrams and microstructures developed by solidification and solid−solid phase transformations *(see also 64.70.Kb Solid–solid transitions)*

81.30.Bx Phase diagrams of metals and alloys

81.30.Dz Phase diagrams of other materials *(for phase diagrams of superconductors, see 74.25.Dw)*

81.30.Fb Solidification

81.30.Hd Constant-composition solid–solid phase transformations: polymorphic, massive, and order–disorder

81.30.Kf Martensitic transformations

81.30.Mh Solid-phase precipitation *(see also 64.75.+g Solubility, segregation, and mixing; phase separation)*

81.40.−z Treatment of materials and its effects on microstructure and properties

81.40.Cd Solid solution hardening, precipitation hardening, and dispersion hardening; aging

81.40.Ef Cold working, work hardening; annealing, post-deformation annealing, quenching, tempering recovery, and crystallization

81.40.Gh Other heat and thermomechanical treatments

81.40.Jj Elasticity and anelasticity, stress-strain relations

81.40.Lm Deformation, plasticity, and creep *(see also 83.50.−v Deformation and flow in rheology)*

81.40.Np Fatigue, corrosion fatigue, embrittlement, cracking, fracture and failure

81.40.Pq Friction, lubrication, and wear

81.40.Rs Electrical and magnetic properties (related to treatment conditions)

81.40.Tv Optical and dielectric properties (related to treatment conditions)

81.40.Vw Pressure treatment *(see also 62.50.+p High-pressure and shock-wave effects in solids and liquids)*

81.40.Wx Radiation treatment (particle and electromagnetic) *(see also 61.80.−x Physical radiation effects, radiation damage)*

. . . . *Etching, corrosion, oxidation, and other surface treatments, see 81.65.−b*

81.65.−b Surface treatments *(see also 85.40.−e Microelectronics: LSI, VLSI, ULSI; integrated circuit fabrication technology)*

81.65.Cf Surface cleaning, etching, patterning *(see also 52.77.Bn Etching and cleaning in physics of plasmas)*

81.65.Kn Corrosion protection *(see also 82.45.Bb Corrosion and passivation in electrochemistry)*

81.65.Lp Surface hardening: nitridation, carburization, carbonitridation

81.65.Mq Oxidation

81.65.Ps Polishing, grinding, surface finishing

81.65.Rv Passivation *(see also 82.45.Bb Corrosion and passivation in electrochemistry)*

81.65.Tx Gettering

81.70.−q Methods of materials testing and analysis *(for specific chemical analysis methods, see 82.80.−d)*

81.70.Bt Mechanical testing, impact tests, static and dynamic loads

81.70.Cv *Nondestructive testing: ultrasonic testing, photoacoustic testing*

81.70.Ex Nondestructive testing: electromagnetic testing, eddy-current testing

81.70.Fy Nondestructive testing: optical methods

81.70.Ha Testing in microgravity environments

81.70.Jb Chemical composition analysis, chemical depth and dopant profiling

81.70.Pg Thermal analysis, differential thermal analysis (DTA), differential thermogravimetric analysis

81.70.Tx Computed tomography

81.90.+c Other topics in materials science (restricted to new topics in section 81)

82. Physical chemistry and chemical physics

. . . . *Electronic structure theory, see also 33.15.−p in Atomic and molecular physics, section 71 in Condensed matter, and 87.15.Aa in Biological and medical physics*

82.20.−w Chemical kinetics and dynamics

82.20.Bc State selected dynamics and product distribution *(see also 34.50.Pi State-to-state scattering analyses in scattering of atoms and molecules)*

82.20.Db Transition state theory and statistical theories of rate constants

82.20.Ej Quantum theory of reaction cross section

82.20.Fd Collision theories; trajectory models

82.20.Gk Electronically non-adiabatic reactions

82.20.Hf Product distribution *(for state selected dynamics and product distribution, see 82.20.Bc)*

82.20.Kh Potential energy surfaces for chemical reactions *(for potential energy surfaces for collisions, see 34.20.Mq in atomic and molecular collisions and interactions)*

82.20.Ln Semiclassical theory of reactions and/or energy transfer

82.20.Nk Classical theories of reactions and/or energy transfer

82.20.Pm Rate constants, reaction cross sections, and activation energies

82.20.Rp State to state energy transfer *(see also 31.70.Hq Time-dependent phenomena, and 34.50.Pi state-to-state scattering analyses—in atomic and molecular physics)*

82.20.Sb Correlation function theory of rate constants and its applications

82.20.Tr Kinetic isotope effects including muonium

82.20.Uv Stochastic theories of rate constants

82.20.Wt Computational modeling; simulation

82.20.Xr Quantum effects in rate constants (tunneling, resonances, etc.)

82.20.Yn Solvent effects on reactivity

82.30.−b Specific chemical reactions; reaction mechanisms

82.30.Cf Atom and radical reactions; chain reactions; molecule-molecule reactions

82.30.Fi Ion–molecule, ion–ion, and charge-transfer reactions *(see also 34.70.+e Charge transfer in atomic and molecular collisions)*

. . . . *Charge transfer in enzymes, see 82.39.Jn*

82.30.Gg Positronium chemistry *(see also 36.10.Dr Positronium, muonium, muonic atoms and molecules in atomic and molecular physics; 78.70.Bj Positron annihilation in interactions of particles and radiation with matter)*

82.30.Hk Chemical exchanges (substitution, atom transfer, abstraction, disproportionation, and group exchange)

82.30.Lp Decomposition reactions (pyrolysis, dissociation, and fragmentation)

82.30.Nr Association, addition, insertion, cluster formation

82.30.Qt Isomerization and rearrangement

82.30.Rs Hydrogen bonding, hydrophilic effects

82.30.Vy Homogeneous catalysis in solution, polymers and zeolites *(for heterogeneous catalysis in zeolites, see 82.75.Qt)*

. . . . *Enzyme kinetics, see 82.39.Fk*

. . . . *Protein folding kinetics, see 87.15.Cc in biological and medical physics*

82.33.−z Reactions in various media

82.33.De Reactions in supercritical fluids

82.33.Fg Reactions in clusters *(see also 36.40.Jn Reactivity of clusters in atomic and molecular physics)*

82.33.Hk Reactions on clusters

82.33.Jx Reactions in zeolites

82.33.Ln Reactions in sol gels, aerogels, porous media

82.33.Nq Reactions in micells

82.33.Pt Solid state chemistry

. . . . *Reactions in complex biological systems, see 82.39.Rt*

82.33.Tb Atmospheric chemistry *(see also 92.60.Hp and 94.10.Fa in geophysics)*

82.33.Vx Reactions in flames, combustion, and explosions

82.33.Xj Plasma reactions (including flowing afterglow and electric discharges)

82.33.Ya Chemistry of MOCVD and other

vapor deposition methods *(for methods of vapor deposition of films and coatings, see 81.15.Gh, Kk in materials science)*

82.35.−x Polymers: properties; reactions; polymerization *(for polymers in electrochemistry, see 82.45.Wx)*

82.35.Cd Conducting polymers

82.35.Ej Nonlinear optics with polymers *(see also 42.65.−k in nonlinear optics)*

82.35.Gh Polymers on surfaces; adhesion *(see also 68.35.Np Adhesion in surfaces and interfaces)*

82.35.Jk Copolymers, phase transitions, structure

82.35.Lr Physical properties of polymers

82.35.Np Nanoparticles in polymers *(see also 81.07.−b Nanoscale materials and structures: fabrication and characterization)*

82.35.Pq Biopolymers, biopolymerization *(see also 87.15.Rn Reactions and kinetics; polymerization in biological and medical physics)*

82.35.Rs Polyelectrolytes

. . . . *Protein properties, folding, see 87.15.Cc and 87.14.Ee in biological and medical physics*

. . . . *Enzymes, see 82.39.Fk and 87.14.Ee*

. . . . *DNA/RNA, see 82.39.Pj and 87.14.Gg*

82.37.−j Single molecule kinetics

82.37.Gk STM and AFM manipulations of a single molecule *(for atom manipulation see 39.25.+k in atomic and molecular physics; see also 81.16.Ta Atom manipulation in methods of nanofabrication and processing)*

82.37.Np Single molecule reaction kinetics, dissociation, etc.

82.37.Rs Single molecule manipulation of proteins and other biological molecules

82.37.Vb Single molecule photochemistry

82.39.−k Chemical kinetics in biological systems *(see also 87.15.Rn Reactions and kinetics; polymerization in biological and medical physics, and 82.45.Tv Bioelectrochemistry)*

82.39.Fk Enzyme kinetics

82.39.Jn Charge (electron, proton) transfer in biological systems

. . . . *Protein folding, see 87.15.Cc in biological and medical physics*

82.39.Pj Nucleic acids, DNA and RNA bases

82.39.Rt Reactions in complex biological systems

82.39.Wj Ion exchange, dialysis, osmosis, electro-osmosis, membrane processes

82.40.−g Chemical kinetics and reactions: special regimes and techniques

. . . . *Chemically reactive flows, see 47.70.Fw in fluid dynamics*

82.40.Bj Oscillations, chaos, and bifurcations

82.40.Ck Pattern formation in reactions with diffusion, flow and heat transfer *(see also 47.54.+r Pattern selection; pattern formation and 47.32.Cc Vortex dynamics in fluid dynamics)*

82.40.Fp Shock wave initiated reactions, high-pressure chemistry *(see also 47.40.Nm Shock wave interactions and shock effects in fluid dynamics, and 62.50.+p high-pressure and shock wave effects in solids and liquids)*

82.40.Np Temporal and spatial patterns in surface reactions

82.40.Qt Complex chemical systems *(for complex biological systems, see 82.39.Rt)*

. . . . *Stochastic theories of chemical kinetics, see 82.20.Uv*

82.45.−h Electrochemistry and electrophoresis

82.45.Aa Electrochemical synthesis *(see also 81.16.Be Chemical synthesis methods in nanofabrication and 81.20.Ka Chemical synthesis; combustion synthesis in materials synthesis)*

82.45.Bb Corrosion and passivation *(see also 81.65.Kn Corrosion protection and 81.65.Rv Passivation in surface treatments)*

82.45.Cc Anodic films

82.45.Fk Electrodes

82.45.Gj Electrolytes *(for polyelectrolytes, see also 82.35.Rs and 82.45.Wx; see also 66.30.Hs Self-diffusion and ionic conduction in nonmetals)*

82.45.Hk Electrolysis

82.45.Jn Surface structure, reactivity and catalysis *(see also 82.65.+r Surface and interface chemistry; heterogeneous catalysis at surfaces)*

82.45.Mp Thin layers, films, monolayers, membranes *(for anodic films, see 82.45.Cc; for surface double layers, see 73.30.+y in electronic structure of surfaces)*

82.45.Qr Electrodeposition and electrodissolution *(see also 81.15.Pq Electrodeposition, electroplating in materials science)*

82.45.Rr Electroanalytical chemistry *(see also 82.80.Fk Electrochemical methods in chemical analysis and related physical methods of analysis)*

82.45.Tv Bioelectrochemistry *(see also 82.39.−k Chemical kinetics in biological systems)*

82.45.Un Dielectric materials in electrochemistry *(see also 77.84.−s Dielectric, piezoelectric, ferroelectric, and antiferroelectric materials)*

82.45.Vp Semiconductor materials in electrochemistry *(see also 81.05.Cy, Dz, Ea, Gc, Hd in specific materials)*

82.45.Wx Polymers and organic materials in electrochemistry *(see also 82.35.−x Polymers: properties; reactions; polymerization)*

82.45.Xy Ceramics in electrochemistry *(see also 81.05.Je, Mh in specific materials)*

82.45.Yz Nanostructured materials in electrochemistry *(for nanofabrication, see 81.16.−c in materials science)*

82.47.−a Applied electrochemistry

82.47.Aa Lithium-ion batteries

82.47.Cb Lead-acid, nickel-metal hydride and other batteries *(for lithium-ion batteries, see 82.47.Aa)*

82.47.Ed Solid-oxide fuel cells (SOFC)

82.47.Gh Proton exchange membrane (PEM) fuel cells

82.47.Jk Photoelectrochemical cells, photoelectrochromic and other hybrid electrochemical energy storage devices *(see also 84.60.Jd Photoelectric conversion, solar cells and arrays)*

82.47.Lh Molten-carbonate fuel cells (MCFC)

82.47.Nj Polymer-electrolyte fuel cells (PEFC)

82.47.Pm Phosphoric-acid fuel cells (PAFC); other fuel cells

82.47.Rs Electrochemical sensors

82.47.Tp Electrochemical displays

82.47.Uv Electrochemical capacitors; supercapacitors

82.47.Wx Electrochemical engineering

82.50.−m Photochemistry *(for single molecule photochemistry, see 82.37.Vb)*

. . . . *Optical spectroscopy, see 32.30.−r and 33.20.−t in atomic and molecular physics; 78.30.−j, 78.35.+c, 78.40.−q, and 78.47.+p in condensed matter physics*

82.50.Bc Processes caused by infrared radiation

82.50.Hp Processes caused by visible and UV light

82.50.Kx Processes caused by X-rays or γ-rays

82.50.Nd Control of photochemical reactions

82.50.Pt Multiphoton processes

. . . . *Potential energy surfaces for photochemistry and spectroscopy, see 31.50.Df*

· · · · *Surface crossings, non-adiabatic couplings, see 31.50.Gh*

82.53.−k **Femtochemistry** *(see also 78.47.+p Time-resolved optical spectroscopies and other ultrafast optical measurements in condensed matter; 42.65.Re Ultrafast processes; optical generation and pulse compression in nonlinear optics)*

82.53.Eb Pump probe studies of photodissociation

82.53.Hn Pump probe experiments with bound states

82.53.Kp Coherent spectroscopy of atoms and molecules

82.53.Mj Femtosecond probing of semiconductor nanostructures *(see also 81.16.−c Methods of nanofabrication and processing)*

82.53.Ps Femtosecond probing of biological molecules

82.53.St Femtochemistry of adsorbed molecules *(for adsorbate structure, see 68.43.Bc, Fg in chemisorption/ physisorption: adsorbates on surfaces)*

82.53.Uv Femtosecond probes of molecules in liquids

82.53.Xa Femtosecond probes of molecules in solids and of molecular solids

82.56.−b **Nuclear magnetic resonance** *(see also 33.25.+k Nuclear resonance and relaxation in atomic and molecular physics; 76.60.−k Nuclear magnetic resonance and relaxation; 76.70.−r Magnetic double resonances and cross effects in condensed matter)*

82.56.Dj High resolution NMR

82.56.Fk Multidemensional NMR

82.56.Hg Multinuclear NMR

82.56.Jn Pulse sequences in NMR

82.56.Lz Diffusion

82.56.Na Relaxation

82.56.Pp NMR of biomolecules

82.56.Ub Structure determination with NMR

· · · · *ENDOR(see 76.70.Dx in condensed matter, and 33.40.+f in atomic and molecular physics)*

· · · · *NMR imaging, see 76.60.Pc in condensed matter*

82.60.−s **Chemical thermodynamics** *(see also 05.70.−a Thermodynamics)*

82.60.Cx Enthalpies of combustion, reaction, and formation

82.60.Fa Heat capacities and heats of phase transitions

82.60.Hc Chemical equilibria and equilibrium constants

82.60.Lf Thermodynamics of solutions

82.60.Nh Thermodynamics of nucleation *(see also 64.60.Qb Nucleation—in equations of state, phase equilibria and phase transitions)*

82.60.Qr Thermodynamics of nanoparticles

· · · · *Irreversible thermodynamics, nonequilibrium thermodynamics, see 05.70.Ln*

82.65.+r **Surface and interface chemistry; heterogeneous catalysis at surfaces** *(for temporal and spatial patterns in surface reactions, see 82.40.Np; see also 82.45.Jn Surface structure, reactivity and catalysis in electrochemistry)*

· · · · *Chemisorption/physisorption: adsorbates on surfaces, see 68.43.−h*

82.70.−y **Disperse systems; complex fluids** *(see also 82.33.−z reactions in various media; for quantum optical phenomena in dispersive media, see 42.50.Nn)*

82.70.Dd Colloids

82.70.Gg Gels and sols

82.70.Kj Emulsions and suspensions

82.70.Rr Aerosols and foams

82.70.Uv Surfactants, micellar solutions, vesicles, lamellae, amphiphilic systems, (hydrophilic and hydrophobic interactions) *(see also 82.30.Rs Hydrogen bonding, hydrophilic effects in specific chemical reactions)*

· · · · *Nanoscale materials and structures, see 81.07.−b*

· · · · *Preparation and assembly of nanostructures, see 81.16.−c*

· · · · *Phase transitions of nanostructures, see 64.70.Nd*

· · · · *Spectroscopy of nanostructures, see 78.67.−n*

82.75.−z **Molecular sieves, zeolites, clathrates, and other complex solids**

82.75.Fq Synthesis, structure determination, structure modeling

82.75.Jn Measurements and modeling of molecule migration in zeolites

82.75.Mj Measurements and simulation of properties (optical, structural) of molecules in zeolites

82.75.Qt Mechanism and kinetics of catalysis in zeolites (measurements or simulations)

82.75.Vx Clusters in zeolites

82.80.−d **Chemical analysis and related physical methods of analysis** *(for related instrumentation, see section 07; for chemical analysis techniques in biophysics, see 87.64.−t)*

82.80.Bg Chromatography

82.80.Dx Analytical methods involving electronic spectroscopy

82.80.Ej X-ray, Mössbauer, and other γ-ray spectroscopic analysis methods

82.80.Fk Electrochemical methods *(see also 82.45.Rr Electroanalytical chemistry; for electrochemical sensors, see 82.47.Rs)*

82.80.Gk Analytical methods involving vibrational spectroscopy

82.80.Ha Analytical methods involving rotational spectroscopy

82.80.Jp Activation analysis and other radiochemical methods

82.80.Kq Energy-conversion spectro-analytical methods (e.g., photoacoustic, photothermal, and optogalvanic spectroscopic methods)

82.80.Ms Mass spectrometry (including SIMS, multiphoton ionization and resonance ionization mass spectrometry, MALDI)

82.80.Nj Fourier transform mass spectrometry

82.80.Pv Electron spectroscopy (x-ray photoelectron (XPS), Auger electron spectroscopy (AES), etc.)

82.80.Qx Ion cyclotron resonance mass spectrometry

82.80.Rt Time of flight mass spectrometry

82.80.Yc Rutherford backscattering (RBS), and other methods of chemical analysis

82.90.+j **Other topics in physical chemistry and chemical physics (restricted to new topics in section 82)**

83. Rheology *(see also section 47 Fluid dynamics)*

83.10.−y **Fundamentals and theoretical**

83.10.Bb Kinematics of deformation and flow

· · · · *Fluid dynamics (non-Newtonian fluids), see 47.50.+d*

83.10.Ff Continuum mechanics *(see also section 46 Continuum mechanics of solids)*

83.10.Gr Constitutive relations

83.10.Kn Reptation and tube theories

83.10.Mj Molecular dynamics, Brownian dynamics

83.10.Pp Particle dynamics

83.10.Rs Computer simulation of molecular and particle dynamics

83.10.Tv Structural and phase changes

83.50.−v **Deformation and flow**

83.50.Ax Steady shear flows, viscometric flow

83.50.Ha Flow in channels

83.50.Jf Extensional flow and combined shear and extension

83.50.Lh Slip boundary effects (interfacial and free surface flows) *(see also 47.45.Gx Slip flows in fluid dynamics)*

83.50.Rp Wall slip and apparent slip

83.50.Uv Material processing (extension, molding, etc.)

83.50.Xa Mixing and blending

83.60.−a Material behavior

83.60.Bc Linear viscoelasticity

83.60.Df Nonlinear viscoelasticity

83.60.Fg Shear rate dependent viscosity

83.60.Hc Normal stress differences and their effects (e.g. rod climbing)

83.60.Jk Extrudate swell

83.60.La Viscoplasticity; yield stress

83.60.Np Effects of electric and magnetic fields

83.60.Pq Time-dependent structure (thixotropy, rheopexy)

83.60.Rs Shear rate-dependent structure (shear thinning and shear thickening)

83.60.St Non-isothermal rheology

83.60.Uv Wave propagation, fracture, and crack healing

83.60.Wc Flow instabilities

83.60.Yz Drag reduction

83.80.−k Material type *(see also 82.70.−y Disperse systems; complex fluids and 82.35.−x Polymers: properties; reactions; polymerization in physical chemistry and chemical physics)*

83.80.Ab Solids: e.g., composites, glasses, semicrystalline polymers

83.80.Fg Granular solids

83.80.Gv Electro- and magnetorheological fluids

83.80.Hj Suspensions, dispersions, pastes, slurries, colloids

83.80.Iz Emulsions and foams

83.80.Jx Reacting systems: thermosetting polymers, chemorheology, rheokinetics

83.80.Kn Physical gels and microgels

83.80.Lz Physiological materials (e.g. blood, collagen, etc.)

83.80.Mc Other natural materials (e.g. wood and other vegetable materials)

83.80.Nb Geological materials: Earth, magma, ice, rocks, etc.

83.80.Qr Surfactant and micellar systems, associated polymers

83.80.Rs Polymer solutions

83.80.Sg Polymer melts

83.80.Tc Polymer blends

83.80.Uv Block copolymers

83.80.Va Elastomeric polymers

83.80.Wx Filled elastomers

83.80.Xz Liquid crystals: nematic, cholesteric, smectic, discotic, etc.

83.80.Ya Processed food

83.85.−c Techniques and apparatus

83.85.Cg Rheological measurements—rheometry

83.85.Ei Optical methods; rheo-optics

83.85.Fg NMR/magnetic resonance imaging *(see also 76.60.Pc NMR imaging in condensed matter)*

83.85.Hf X-ray and neutron scattering

83.85.Jn Viscosity measurements

83.85.Lq Normal stress difference measurements

83.85.Ns Data analysis (interconversion of data computation of relaxation and retardation spectra; time-temperature superposition, etc.)

83.85.Pt Computational fluid dynamics *(see also 02.70.−c—in mathematical methods in physics; 47.11.+j Computational methods in fluid dynamics)*

83.85.Rx Extensional flow measurement

83.85.St Stress relaxation

83.85.Tz Creep and/or creep recoil

83.85.Vb Small amplitude oscillatory shear (dynamic mechanical analysis)

83.90.+s Other topics in rheology (restricted to new topics in section 83)

84. Electronics; radiowave and microwave technology; direct energy conversion and storage

84.30.−r Electronic circuits *(for integrated circuits, see 85.40.−e, for microwave circuits, see 84.40.Dc)*

84.30.Bv Circuit theory (including computer-aided circuit design and analysis)

84.30.Jc Power electronics; power supply circuits *(see also 84.70.+p High-current and high-voltage technology; for superconducting high-power technology, see 84.71.−b)*

84.30.Le Amplifiers

84.30.Ng Oscillators, pulse generators, and function generators

84.30.Qi Modulators and demodulators; discriminators, comparators, mixers, limiters, and compressors

84.30.Sk Pulse and digital circuits

84.30.Vn Filters

84.32.−y Passive circuit components *(see also 07.50.+q Electrical and electronic components, instruments, and techniques)*

84.32.Dd Connectors, relays, and switches

84.32.Ff Conductors, resistors (including thermistors, varistors, and photoresistors)

84.32.Hh Inductors and coils; wiring

84.32.Tt *Capacitors (for electrochemical capacitors and supercapacitors, see 82.47.Uv)*

84.32.Vv Fuses

84.35.+i Neural networks *(for optical neural networks, see 42.79.Ta, see also 07.05.Mh Neural networks, fuzzy logic, artificial intelligence in computers in experimental physics; see also 87.18.Sn in multicellular phenomena)*

84.37.+q Electric variable measurements (including voltage, current, resistance, capacitance, inductance, impedance, and admittance, etc.)

84.40.−x Radiowave and microwave (including millimeter wave) technology

· · · · *Microwave, submillimeter wave, and radiowave receivers and detectors, see 07.57.Kp*

· · · · *Microwave and radiowave spectrometers, see 07.57.Pt*

· · · · *Electromagnetic wave propagation, see 41.20.Jb*

84.40.Az Waveguides, transmission lines, striplines

84.40.Ba Antennas: theory, components and accessories *(for plasma interactions with antennas, see 52.40.Fd in plasma physics)*

84.40.Dc Microwave circuits

84.40.Fe Microwave tubes (e.g., klystrons, magnetrons, traveling-wave, backward-wave tubes, etc.)

84.40.Ik Masers; gyrotrons (cyclotron-resonance masers)

84.40.Lj Microwave integrated electronics

84.40.Ua Telecommunications: signal transmission and processing; communication satellites *(for optical communications, see 42.79.Sz in optics)*

84.40.Xb Telemetry: remote control, remote sensing; radar

84.47.+w Vacuum tubes *(see also 85.45.−w Vacuum microelectronics)*

· · · · *Phototubes, see 85.60.Ha*

· · · · *Microwave tubes, see 84.40.Fe*

84.50.+d Electric motors

84.60.−h Direct energy conversion and storage *(see also 89.30.−g Energy resources; for electrochemical conversion, see 82.47.−a)*

84.60.Bk Performance characteristics of energy conversion systems; figure of merit

84.60.Jt Photoelectric conversion: solar cells and arrays *(for solar collectors and concentrators, see 42.79.Ek in optics)*

84.60.Lw Magnetohydrodynamic conversion

(for MHD generators, see 52.75.Fk—in plasma physics)

84.60.Ny Thermionic conversion *(for thermionic generators, see 52.75.Fk—in plasma physics)*

84.60.Rb Thermoelectric, electrogasdynamic and other direct energy conversion

84.60.Ve Energy storage systems, including capacitor banks

84.70.+p High-current and high-voltage technology: power systems; power transmission lines and cables *(for superconducting cables, see 84.71.Fk)*

84.71.−b Superconducting high-power technology *(see also 84.30.Jc Power electronics; power supply circuits)*

84.71.Ba Superconducting magnets; magnetic levitation devices

84.71.Fk Superconducting cables

84.71.Mn Superconducting wires, fibers, and tapes

84.90.+a Other topics in electronics, radiowave and microwave technology, and direct energy conversion and storage (restricted to new topics in section 84)

85. Electronic and magnetic devices; microelectronics

. . . . *Vacuum tubes, see 84.47.+w*

. . . . *Microwave tubes, see 84.40.Fe*

. . . . *Phototubes, see 85.60.Ha*

. . . . *Conductors, resistors, and inductors, see 84.32.Ff, Hh*

85.25.−j Superconducting devices

85.25.Am Superconducting device characterization, design, and modeling

85.25.Cp Josephson devices

85.25.Dq Superconducting quantum interference devices (SQUIDs)

85.25.Hv Superconducting logic elements and memory devices; microelectronic circuits

85.25.Oj Superconducting optical, x-ray, and γ-ray detectors (SIS, NIS, transition edge)

85.25.Pb Superconducting infrared, submillimeter and millimeter wave detectors

. . . . *High power superconducting devices, see 84.71.−b*

85.25.Qc Superconducting surface acoustic wave devices and other superconducting devices

85.30.−z Semiconductor devices *(for photodiodes, phototransistors, and photoresistors, see 85.60.Dw; for laser diodes, see 42.55.Px)*

85.30.De Semiconductor-device characterization, design, and modeling

85.30.Fg Bulk semiconductor and conductivity oscillation devices (including Hall effect devices, space-charge- limited devices, and Gunn effect devices)

85.30.Hi Surface barrier, boundary, and point contact devices

85.30.Kk Junction diodes

85.30.Mn Junction breakdown and tunneling devices (including resonance tunneling devices)

85.30.Pq Bipolar transistors

85.30.Rs Thyristors

85.30.Tv Field effect devices

85.35.−p Nanoelectronic devices

85.35.Be Quantum well devices (quantum dots, quantum wires, etc.)

85.35.Ds Quantum interference devices

85.35.Gv Single electron devices

85.35.Kt Nanotube devices

85.40.−e Microelectronics: LSI, VLSI, ULSI; integrated circuit fabrication technology *(see also 85.45.−w Vacuum microelectronics)*

. . . . *Microwave integrated electronics, see 84.40.Lj*

. . . . *Integrated optics, see 42.82.−m*

. . . . *Superconducting logic elements and memory devices; microelectronic circuits, see 85.25.Hv*

85.40.Bh Computer-aided design of microcircuits; layout and modeling

85.40.Hp Lithography, masks and pattern transfer

. . . . *Micro- and nano-electromechanical systems (MEMS/NEMS) and devices, see 85.85.+j*

85.40.Ls Metallization, contacts, interconnects; device isolation

85.40.Qx Microcircuit quality, noise, performance, and failure analysis

85.40.Ry Impurity doping, diffusion and ion implantation technology

85.40.Sz Deposition technology *(for plasma applications in deposition technology, see 52.77.Dq)*

. . . . *Bipolar integrated circuits, see 85.30.Pq*

. . . . *Field effect integrated circuits, see 85.30.Tv*

85.40.Xx Hybrid microelectronics; thick films

85.45.−w Vacuum microelectronics

. . . . *Microwave vacuum microelectronic devices, see 84.40.−x*

85.45.Bz Vacuum microelectronic device characterization, design, and modeling

85.45.Db Field emitters and arrays, cold electron emitters

85.45.Fd Field emission displays (FEDs)

. . . . *Capacitors, see 84.32.Tt*

85.50.−n Dielectric, ferroelectric, and piezoelectric devices

85.50.Gk Non-volatile ferroelectric memories

85.60.−q Optoelectronic devices *(see also 42.79.−e Optical elements, devices and systems)*

85.60.Bt Optoelectronic device characterization, design, and modeling

85.60.Dw Photodiodes; phototransistors; photoresistors

85.60.Gz Photodetectors (including infrared and CCD detectors) *(for superconducting infrared detectors, see 85.25.Pb; for superconducting optical, x-ray and γ-ray detectors, see 85.25.Oj; see also 07.57.Kp in instruments)*

85.60.Ha Photomultipliers; phototubes and photocathodes

85.60.Jb Light-emitting devices

85.60.Pg Display systems *(for field emission display, see 85.45.Fd, for optical display devices, see 42.79.Kr; for electrochemical displays, see 82.47.Tp see also 07.07.Hj Display and recording equipment, oscilloscopes, TV cameras, etc.)*

85.65.+h Molecular electronic devices

85.70.−w Magnetic devices

. . . . *Molecular magnets, see 75.50.Xx*

. . . . *Magnets, see 07.55.Db*

. . . . *Superconducting magnets and magnetic levitation devices, see 84.71.Ba*

. . . . *Beam bending magnets, see 41.85.Lc*

85.70.Ay Magnetic device characterization, design, and modeling

85.70.Ec Magnetostrictive, magnetoacoustic, and magnetostatic devices *(for magnetostrictive transducers, see 43.38.Ct—in acoustics appendix)*

. . . . *Magnetic recording materials, see 75.50.Ss*

85.70.Ge Ferrite and garnet devices

85.70.Kh Magnetic thin film devices: magnetic heads (magnetoresistive, inductive, etc.); domain-motion devices, etc.

85.70.Li Other magnetic recording and storage devices (including tapes, disks, and drums)

85.70.Rp Magnetic levitation, propulsion and control devices *(for superconducting-magnetic levitation devices, see 84.71.Ba)*

85.70.Sq Magnetooptical devices

85.75.−d **Magnetoelectronics; spintronics: devices exploiting spin polarized transport or integrated magnetic fields**
85.75.Bb Magnetic memory using giant magnetoresistance
85.75.Dd Magnetic memory using magnetic tunnel junctions
85.75.Ff Reprogrammable magnetic logic
85.75.Hh Spin polarized field effect transistors
85.75.Mm Spin polarized resonant tunnel junctions
85.75.Nn Hybrid Hall devices
85.75.Ss Magnetic field sensors using spin polarized transport

85.80.−b **Thermoelectromagnetic and other devices** *(for acoustoelectric devices, see 43.38.−p—in acoustics appendix; for electrochemical devices, see 82.47.−a)*
85.80.Fi Thermoelectric devices
85.80.Jm Magnetoelectric devices
85.80.Lp Magnetothermal devices

85.85.+j **Micro- and nano-electromechanical systems (MEMS/NEMS) and devices**

85.90.+h **Other topics in electronic and magnetic devices and microelectronics (restricted to new topics in section 85)**

87. Biological and medical physics

87.10.+e **General theory and mathematical aspects**

87.14.−g **Biomolecules: types**
87.14.Cc Lipids
87.14.Ee Proteins
87.14.Gg DNA, RNA

87.15.−v **Biomolecules: structure and physical properties**
87.15.Aa Theory and modeling; computer simulation
87.15.By Structure and bonding
87.15.Cc Folding and sequence analysis
87.15.He Dynamics and conformational changes
87.15.Kg Molecular interactions; membrane-protein interactions
87.15.La Mechanical properties
87.15.Mi Spectra, photodissociation, and photoionization; luminescence
87.15.Nn Properties of solutions; aggregation and crystallization of macromolecules
87.15.Rn Reactions and kinetics; polymerization *(see also 82.39.−k Chemical kinetics in biological systems and 82.35.Pq Biopolymers, biopolymerization in physical chemistry and chemical physics)*
87.15.Tt Electrophoresis *(see also 82.45.−h Electrochemistry and electrophoresis)*
87.15.Vv Diffusion
87.15.Ya Fluctuations

87.16.−b **Subcellular structure and processes**
87.16.Ac Theory and modeling; computer simulation
87.16.Dg Membranes, bilayers, and vesicles
87.16.Gj Cell walls
87.16.Ka Filaments, microtubules, their networks, and supramolecular assemblies
87.16.Nn Motor proteins (myosin, kinesin dynein)
87.16.Qp Pseudopods, lamellipods, cilia, and flagella
87.16.Sr Chromosomes, histones
87.16.Tb Organelles
87.16.Uv Active transport processes; ion channels
87.16.Xa Signal transduction
87.16.Yc Regulatory chemical networks

87.17.−d **Cellular structure and processes**
87.17.Aa Theory and modeling; computer simulation
87.17.Ee Growth and division
87.17.Jj Cell locomotion; chemotaxis and related directed motion
87.17.Nn Electrophysiology of nerve cells

87.18.−h **Multicellular phenomena**
87.18.Bb Computer simulation
87.18.Ed Aggregation and other collective behavior of motile cells
87.18.Hf Spatiotemporal pattern formation in cellular populations
87.18.La Morphogenesis
87.18.Pj Chemical waves
87.18.Sn Neural networks

87.19.−j **Properties of higher organisms**
. . . . *Physiological optics, see 42.66.−p*
. . . . *Physiological acoustics, see 43.64.+r*
. . . . *Psychological acoustics, see 43.66.+y*
. . . . *Speech production, see 43.70.+i*
. . . . *Speech perception, see 43.71.+m*
. . . . *Speech processing and communication systems, see 43.72.+g*
87.19.Bb Sensory perceptions
87.19.Dd Information processing in vision and hearing
87.19.Ff Muscles
87.19.Hh Cardiac dynamics
87.19.Jj Circadian rhythms
87.19.La Neuroscience
87.19.Nn Electrophysiology
87.19.Pp Biothermics
87.19.Rr Mechanical properties of tissues and organs
87.19.St Movement and locomotion
87.19.Tt Rheology of body fluids
87.19.Uv Haemodynamics, pneumodynamics
87.19.Xx Diseases

87.23.−n **Ecology and evolution**
87.23.Cc Population dynamics and ecological pattern formation
87.23.Ge Dynamics of social systems
87.23.Kg Dynamics of evolution

87.50.−a **Effects of radiation and external fields on biomolecules, cells and higher organisms**
87.50.Gi Ionizing radiations (ultraviolet, x-rays, γ-rays, ions, electrons, positrons, neutrons, and mesons, etc.)
87.50.Hj Optical radiation (near ultraviolet, visible, and infrared)
87.50.Jk Radio frequency and microwave radiation (power lines)
87.50.Kk Sound and ultrasound
87.50.Mn Magnetic fields
87.50.Rr Electric fields

87.52.−g **Radiation monitoring, control, and safety**
87.52.Df Low LET: therapeutic and diagnostic x-rays and electrons
87.52.Ga Low LET: associated neutron shielding and measurement
87.52.Ln High LET
87.52.Px Risk/benefit analysis
87.52.Tr Regulatory issues

87.53.−j **Ionizing-radiation therapy physics**
87.53.Bn Photon dosimetry: theory and algorithms
87.53.Dq Photon dosimetry: measurements
87.53.Fs Electron and positron dosimetry: theory and algorithms
87.53.Hv Electron and positron dosimetry: measurements
87.53.Jw Brachytherapy
87.53.Kn Conformal radiation treatment
87.53.Ly Stereotactic radiosurgery
87.53.Mr Beam intensity modification: wedges, compensators
87.53.Na Radioimmunotherapy
87.53.Oq Portal imaging in therapy
87.53.Pb Proton, neutron, and heavier particle dosimetry: theory and algorithms
87.53.Qc Proton, neutron, and heavier particle dosimetry: measurements
87.53.Rd Microdosimetry
87.53.St Record and verify systems and applications
87.53.Tf Treatment planning, optimization, tissue response factors, and dose-volume analysis

87.53.Uv Collimation
87.53.Vb Simulation
87.53.Wz Monte Carlo applications
87.53.Xd Quality assurance in radiotherapy

87.54.−n Non-ionizing radiation therapy physics
87.54.Br Thermotherapy (hyperthermia and cryogenic therapy)
87.54.Dt Electrotherapy
87.54.Fj Photodynamic therapy
87.54.Hk Sound and ultrasound therapy/ lithotripsy

87.56.−v Radiation therapy equipment
87.56.By Radiation generators
87.56.Da Ancillary equipment
87.56.Fc Quality assurance equipment

87.57.−s Medical imaging: general
87.57.Ce Image quality: contrast, resolution, noise, etc.
87.57.Gg Image reconstruction and registration
87.57.Nk Image analysis
87.57.Ra Computer-aided diagnosis

87.58.−b Nuclear medicine imaging, dosimetry, labeling, metabolic studies
87.58.Ce Single photon emission computed tomography (SPECT)
87.58.Fg Positron emission tomography (PET)
87.58.Ji Radiopharmaceuticals
87.58.Mj Digital imaging
87.58.Pm Scintillation cameras
87.58.Sp Dosimetry
87.58.Vr Quantitative measurements and scanning
87.58.Xs Bone densitometry

87.59.−e X-ray imaging
87.59.Bh X-ray radiography
87.59.Ci Fluoroscopy
87.59.Dj Angiography
87.59.Ek Mammography
87.59.Fm Computed tomography (CT)
87.59.Hp Digital radiography
87.59.Jq Transmission imaging
87.59.Ls Bone densitometry

87.61.−c Magnetic resonance imaging
87.61.Cd Pulse sequences for imaging
87.61.Ff Instrumentation
87.61.Lh Angiography and macroscopic flow estimation
87.61.Pk Clinical imaging studies

87.62.+n Medical imaging equipment

87.63.−d Non-ionizing radiation equipment and techniques
87.63.Df Ultrasonography
87.63.Hg Thermography
87.63.Lk Visible radiation: diaphanography, transillumination, laser imaging
87.63.Pn Electrical impedance tomography (EIT)

87.64.−t Spectroscopic and microscopic techniques in biophysics and medical physics *(for spectrometers, see section 07 Instruments, apparatus, and components common to several branches of physics and astronomy)*
87.64.Aa Computer simulation
87.64.Bx Electron, neutron and x-ray diffraction and scattering
87.64.Cc Scattering of visible, uv, and infrared radiation
87.64.Dz Scanning tunneling and atomic force microscopy
87.64.Ee Electron microscopy
87.64.Fb EXAFS spectroscopy
87.64.Gb X-ray spectroscopy *(see also 87.64.Fb EXAFS spectroscopy)*
87.64.Hd EPR and NMR spectroscopy
87.64.Je Infrared and Raman spectroscopy
87.64.Lg Electron and photoelectron spectroscopy
87.64.Ni Optical absorption, magnetic circular dichroism, and fluorescence spectroscopy
87.64.Pj Mössbauer spectroscopy
87.64.Rr Light microscopy: bright-field, dark-field, phase contrast, DIC
87.64.Tt Confocal microscopy
87.64.Vv Multiphoton microscopy
87.64.Xx Near-field scanning optical microscopy

87.65.+y Aerospace bio- and medical physics (effects of accelerations, weightlessness, and space environment)

87.66.−a Radiation measurement
87.66.Cd Films: silver bromide based, radiochromic, etc.
87.66.Ff Chemical dosimetry
87.66.Jj Ionization dosimetry
87.66.Na Calorimetric dosimetry
87.66.Pm Solid state detectors
87.66.Sq Thermoluminescence, bioluminescence, etc.
87.66.Uv Magnetic resonance
87.66.Xa Phantoms

87.68.+z Biomaterials and biological interfaces

87.80.−y Biological techniques and instrumentation; biomedical engineering
87.80.Cc Optical trapping
87.80.Fe Micromanipulators
87.80.Jg Patch clamping
87.80.Mj Micromachining
87.80.Pa Morphometry and stereology
87.80.Rb Tissue and cellular engineering and biotechnology
87.80.Tq Biological signal processing and instrumentation
87.80.Vt Dynamical, regulatory, and integrative biology
87.80.Xa Neural engineering

87.83.+a Biomedical applications of nanotechnology

87.90.+y Other topics in biological and medical physics (restricted to new topics in section 87)

89. Other areas of applied and interdisciplinary physics

89.20.−a Interdisciplinary applications of physics
89.20.Bb Industrial and technological research and development
89.20.Dd Military technology and weapons systems; arms control
89.20.Ff Computer science and technology
89.20.Hh World Wide Web, Internet
89.20.Kk Engineering *(for electrochemical engineering, see 82.47.Wx)*

89.30.−g Energy resources *(see also 84.60.−h Direct energy conversion and storage)*
89.30.Aa Fossil fuels
89.30.Cc Solar power
89.30.Ee Hydroelectric, hydrothermal, geothermal and wind power
89.30.Gg Nuclear fission power *(for fission reactors, see 28.41.−i and 28.50.−k in nuclear physics)*
89.30.Jj Nuclear fusion power *(for fusion reactors, see 28.52.−s in nuclear physics)*

89.40.−a Transportation
89.40.Bb Land transportation
89.40.Cc Water transportation
89.40.Dd Air transporation

89.60.−k Environmental studies *(for ecology, see 87.23.−n in biological and medical physics)*
89.60.Ec Environmental safety
89.60.Fe Environmental regulations
89.60.Gg Impact of natural and man-made disasters

89.65.−s Social and economic systems
89.65.Cd Demographic studies
89.65.Ef Social organizations; anthropology
89.65.Gh Economics; econophysics, financial markets, business and management
89.65.Lm Urban planning and construction

89.70.+c Information theory and communication theory *(for telecommunications, see 84.40.Ua; for optical communications, see 42.79.Sz)*

89.75.−k **Complex systems**

89.75.Da Systems obeying scaling laws

89.75.Fb Structures and organization in complex systems

89.75.Hc Networks and genealogical trees

89.75.Kd Patterns

89.90.+n **Other topics in areas of applied and interdisciplinary physics(restricted to new topics in section 89)**

90. GEOPHYSICS, ASTRONOMY, AND ASTROPHYSICS

91. Solid Earth physics

91.10.−v **Geodesy and gravity**
91.10.By Mathematical geodesy; general theory
91.10.Da Cartography
91.10.Fc Space geodetic surveys
91.10.Jf Topography; geometric observations
91.10.Kg Crustal movements
91.10.Lh Photogrammetry
91.10.Nj Rotational variations; polar wobble
91.10.Pp Gravimetric measurements and instruments
91.10.Qm Harmonics of the gravity potential field
. . . . *Relations of gravity observations to tectonics and isostasy, see 91.45.Sx*
91.10.Rn Rheology of lithosphere and mantle
91.10.Sp Satellite orbits
91.10.Tq Earth tides
91.10.Vr Ocean/Earth/atmosphere interaction
91.10.Ws Reference systems

91.25.−r **Geomagnetism and paleomagnetism; geoelectricity**
91.25.Cw Origins and models of the magnetic field; dynamo theories
91.25.Dx Archeomagnetism
91.25.Ey Interactions between exterior sources and interior properties
91.25.Ga Spatial variations: all harmonics and anomalies
91.25.Jc Spatial variations attributed to sea floor spreading
91.25.Le Time variations: diurnal to secular
91.25.Mf Reversals
91.25.Ng Paleomagnetism
91.25.Ph Magnetostratigraphy
91.25.Qi Geoelectricity; electromagnetic induction and conductivity (magnetotelluric effects)

91.30.−f **Seismology**
91.30.Bi Seismic sources (mechanisms, magnitude, moment frequency spectrum)
91.30.Dk Seismicity: space and time distribution
91.30.Fn Surface and body waves
91.30.Ks Free oscillations (periods less than 12 hours)
91.30.Mv Strong motions and shock waves
91.30.Nw Tsunamis *(for dynamics of oceans, see 92.10.Dh and 92.10.Fj)*
91.30.Px Phenomena related to earthquake prediction
91.30.Rz Explosion seismology
91.30.Tb Volcano seismology
91.30.Vc Continental crust seismology
91.30.Ye Oceanic crust seismology

91.35.−x **Earth's interior structure and properties**
91.35.Cb Models of interior structure
91.35.Dc Heat flow; geothermy
91.35.Ed Structure of the Earth's interior below the upper mantle
91.35.Gf Structure of the crust and upper mantle
91.35.Lj Composition of Earth's interior
91.35.Nm Geochronology
91.35.Pn Tomography of the Earth's interior *(see also 91.30.−f Seismology)*

91.40.−k **Volcanology**
91.40.Bp Ash deposits
91.40.Dr Atmospheric effects *(see also 92.60.Mt Particles and aerosols—in Meteorology)*
91.40.Ft Eruptions
91.40.Hw Lava

91.45.−c **Physics of plate tectonics**
91.45.Cg Continental margins
91.45.Dh Plate tectonics
91.45.Ei Neotectonics
91.45.Fj Convection currents
91.45.Pt Slow vertical crustal movements (including isostasy and postglacial phenomena)
91.45.Qv Tomography of plate tectonics
91.45.Sx Relations of gravity observations to tectonics and isostasy
91.45.Ty Folds and Folding
91.45.Vz Fractures and faults
91.45.Yb Pluton emplacement

91.50.−r **Marine geology and geophysics**
91.50.Cw Beach, coastal, and shelf processes
91.50.Ey Ocean bottom processes *(for ocean basin thermometry, see 43.30.Qd—in acoustics appendix)*
91.50.Ga Bathymetry and noncoastal underwater morphology
91.50.Jc Turbidity currents, sedimentation *(for acoustics of sediments, see 43.30.Ma in acoustics appendix)*

91.60.−x **Physical properties of rocks and minerals** *(for rheological properties of geological materials, see 83.80.Nb)*
91.60.Ba Elasticity, fracture, and flow
91.60.Dc Creep and deformation
91.60.Ed Crystal structure and defects
91.60.Fe Equations of state
91.60.Gf High-pressure behavior
91.60.Hg Phase changes
91.60.Ki Thermal properties
91.60.Lj Acoustic properties
91.60.Mk Optical properties
91.60.Pn Magnetic and electric properties; environmental magnetism

91.65.−n **Geophysical aspects of geology, mineralogy, and petrology** *(for geophysical prospecting, see 43.40.Ph—in acoustics appendix)*
91.65.Br Geochemical cycles
91.65.Dt Isotopic composition/chemistry
91.65.Fw Low-temperature geochemistry
91.65.Hy Organic geochemistry
91.65.Nd Trace elements
91.65.Rg Mineral occurrences and deposits
91.65.Ti Sedimentary petrology
91.65.Vj Major element composition

91.70.−c **Information related to geologic time**
91.70.Bf Cenozoic
91.70.Dh Mesozoic
91.70.Fj Paleozoic
91.70.Hm Precambrian

91.90.+p **Other topics in solid Earth physics (restricted to new topics in section 91)**

92. Hydrospheric and atmospheric geophysics

92.10.−c **Physics of the oceans**
92.10.Bf Physical properties of seawater
92.10.Cg Capillary waves
92.10.Dh Dynamics of the deep ocean
92.10.Ei Coriolis effects
92.10.Fj Dynamics of the upper ocean
92.10.Gk El Nino
92.10.Hm Surface waves, tides, and sea level
92.10.Jn Seiches
92.10.Kp Sea−air energy exchange processes
92.10.Lq Turbulence and diffusion
92.10.Mr Thermohaline structure and circulation
92.10.Ns Fine structure and microstructure
92.10.Pt Optical properties of sea water
92.10.Rw Sea ice
92.10.Sx Coastal and estuarine oceanography
92.10.Ty Fronts and jets
92.10.Vz Underwater sound *(see also 43.30.+m in acoustics)*
92.10.Wa Sediment transport
92.10.Yb Hydrography *(for ocean parameter estimation by acoustical methods, see 43.30.Pc —in acoustics appendix)*
. . . . *Marine geology and geophysics, see 91.50.−r*

92.20.−h **Interdisciplinary aspects of oceanography**

92.20.Bk Aerosols
92.20.Cm Chemistry of the ocean
92.20.Gr Ocean energy extraction
92.20.Hs Anoxic environments
92.20.Jt Biological aspects of oceanography
92.20.Kv Photochemistry
92.20.Lw Photosynthesis
92.20.Mx Physicochemical properties
92.20.Ny Marine pollution
92.20.Pz Bacteria
92.20.Rb Plankton
92.20.Td Radioactivity

92.40.−t Hydrology and glaciology
92.40.Cy Modeling; general theory
92.40.Ea Precipitation
92.40.Fb Rivers, runoff, and streamflow
92.40.Gc Erosion and sedimentation
92.40.Je Evaporation
92.40.Kf Groundwater
92.40.Lg Soil moisture
92.40.Ni Limnology
92.40.Qk Water quality and water resources
92.40.Rm Snow
92.40.Sn Ice
92.40.Vq Glaciers

92.60.−e Meteorology *(see also 43.28.+h Aeroacoustics and atmospheric sound; 42.68.−w Atmospheric optics; 94.10.Dy Atmospheric structure, pressure, density, and temperature)*
92.60.Bh General circulation
92.60.Dj Gravity waves, tides, and compressional waves
92.60.Ek Convection, turbulence, and diffusion
92.60.Fm Boundary layer structure and processes
92.60.Gn Winds and their effects
92.60.Hp Chemical composition and chemical interactions
92.60.Jq Water in the atmosphere (humidity, clouds, evaporation, precipitation)
92.60.Ls Ionic interactions and processes
92.60.Mt Particles and aerosols *(see also 94.20.−y Physics of the ionosphere)*
92.60.Nv Cloud physics; stratus and cumulus clouds
92.60.Pw Atmospheric electricity
92.60.Qx Storms
92.60.Ry Climatology
92.60.Sz Air quality and air pollution
92.60.Ta Interaction of atmosphere with electromagnetic waves; propagation
92.60.Vb Solar radiation
92.60.Wc Weather analysis and prediction

92.70.−j Global change *(see also 92.60.−e Meteorology)*
92.70.Cp Atmosphere
92.70.Er Biogeochemical processes
92.70.Gt Climate dynamics
92.70.Jw Oceans
92.70.Ly Water cycles

92.90.+x Other topics in hydrospheric and atmospheric geophysics (restricted to new topics in section 92)

93. Geophysical observations, instrumentation, and techniques

93.30.−w Information related to geographical regions
93.30.Bz Africa
93.30.Ca Antarctica
93.30.Db Asia
93.30.Fd Australia
93.30.Ge Europe
93.30.Hf North America
93.30.Jg South America
93.30.Kh Large islands (e.g., Greenland)
93.30.Li Arctic Ocean
93.30.Mj Atlantic Ocean
93.30.Nk Indian Ocean
93.30.Pm Pacific Ocean
93.30.Qn Southern Ocean
93.30.Rp Regional seas
93.30.Sq Polar regions
93.30.Tr Temperate regions
93.30.Vs Tropical regions

93.55.+z International organizations, national and international programs

93.65.+e Data acquisition and storage

93.85.+q Instrumentation and techniques for geophysical research

94. Aeronomy and magnetospheric physics

94.10.−s Physics of the neutral atmosphere *(for atmospheres of the planets, see 96.35.Hv)*
94.10.Bw General properties of the high atmosphere
94.10.Dy Atmospheric structure, pressure, density, and temperature (stratosphere, mesosphere, thermosphere, exosphere) *(see also 92.60.−e Meteorology and 92.70.−j Global change)*
94.10.Fa Atmospheric composition (atomic or molecular), chemical reactions and processes *(see also 82.33.Tb Atmospheric chemistry in physical chemistry and chemical physics)*
94.10.Gb Absorption and scattering of radiation
94.10.Jd Tides, waves, and winds
94.10.Lf Convection, diffusion, mixing, turbulence, and fallout
94.10.Nh Cosmic dust
94.10.Rk Aurora and airglow

94.20.−y Physics of the ionosphere *(for ionospheres of the planets, see 96.35.Kx; for radiowave propagation, see 41.20.Jb in electromagnetism; see also section 52 Physics of plasmas and electric discharges)*
94.20.Bb Wave propagation
94.20.Dd Ionospheric structure (*D, E, F,* and topside regions) including steady-state ion densities and temperatures
94.20.Ee *D* region
94.20.Gg *E* region
94.20.Ji *F* region
94.20.Kj Polar cap ionosphere
94.20.Lk Topside region
94.20.Mm Plasmasphere
94.20.Pp Plasmapause
94.20.Qq Particle precipitation
94.20.Rr Interactions between waves and particles
94.20.Ss Electric fields
94.20.Tt Ionospheric soundings
94.20.Vv Ionospheric disturbances and modifications
94.20.Ww Plasma motion, convection, or circulation
94.20.Yx Interaction between ionosphere and magnetosphere

94.30.−d Physics of the magnetosphere *(for magnetospheres of the planets, see 96.35.Kx; for radiowave propagation, see 41.20.Jb in electromagnetism; see also section 52 Physics of plasmas and electric discharges)*
94.30.Bg Magnetic coordinate systems
94.30.Ch Magnetospheric configuration
94.30.Di Magnetopause
94.30.Ej Magnetic tail
94.30.Fk Plasma motion, convection, or circulation
94.30.Gm Plasma instabilities
94.30.Hn Trapped particles
94.30.Jp Ring currents
94.30.Kq Electric fields
94.30.Lr Magnetic storms, substorms
94.30.Ms Magnetic pulsations
94.30.Tz Waves: propagation and excitation
94.30.Va Magnetosheath; interaction with interplanetary space (including solar wind) *(for cosmic-ray interactions, see 13.85.Tp in elementary particle physics; see also 96.40.−z Cosmic rays—in Astronomy)*

94.80.+g Instrumentation for aeronomy

and magnetospheric studies *(see also 95.55.−n Astronomical and space-research instrumentation in astronomy; 07.87.+v spaceborne and space research instruments, apparatus, and components in instruments)*

94.90.+m Other topics in aeronomy and magnetospheric physics (restricted to new topics in section 94)

95. Fundamental astronomy and astrophysics; instrumentation, techniques, and astronomical observations

95.10.−a Fundamental astronomy

95.10.Ce Celestial mechanics (including *n*-body problems) (see also 45.50.Pk in classical mechanics of discrete systems)

. . . . *Dynamics and kinematics of stellar systems, see 98.10.+z*

95.10.Eg Orbit determination and improvement

95.10.Fh Chaotic dynamics *(see also 05.45.−a Nonlinear dynamics and nonlinear dynamical systems)*

95.10.Gi Eclipses, transits, and occultations

95.10.Jk Astrometry and reference systems

95.10.Km Ephemerides, almanacs, and calendars

95.30.−k Fundamental aspects of astrophysics

95.30.Cq Elementary particle processes *(see also section 26 Nuclear astrophysics)*

95.30.Dr Atomic processes and interactions *(see also section 32 Atomic properties and interactions with photons; section 34 Atomic and molecular collision processes and interactions)*

95.30.Ft Molecular and chemical processes and interactions *(see also section 33 Molecular properties and interactions with photons; section 34 Atomic and molecular collision processes and interactions)*

95.30.Gv Radiation mechanisms; polarization

95.30.Jx Radiative transfer; scattering

95.30.Ky Atomic and molecular data, spectra, and spectral parameters (opacities, rotation constants, line identification, oscillator strengths, *gf* values, transition probabilities, etc.) *(see also 32.10.−f, 32.30.−r, 32.70.−n, 33.15.−e, 33.20.−t, and 33.70.−w in atomic and molecular physics)*

95.30.Lz Hydrodynamics

95.30.Qd Magnetohydrodynamics and plasmas *(see also 52.30.Cv and 52.72.+v in physics of plasmas)*

95.30.Sf Relativity and gravitation *(see also section 04 General relativity and gravitation; 98.80.Jk Mathematical and relativistic aspects of cosmology)*

95.30.Tg Thermodynamic processes, conduction, convection, equations of state *(see also 05.70.−a Thermodynamics)*

95.30.Wi Dust processes (condensation, evaporation, sputtering, mantle growth, etc.)

95.35.+d Dark matter (stellar, interstellar, galactic, and cosmological) *(see also 95.30.Cq Elementary particle processes; for brown dwarfs, see 97.20.Vs; for galactic halos, see 98.35.Gi or 98.62.Gq; for models of the early Universe, see 97.10.Fy)*

95.40.+s Artificial Earth satellites *(for lunar and planetary probes, see 95.55.Pe; see also 07.87.+v in instruments, apparatus, and components common to several branches of physics and astronomy)*

95.45.+i Observatories and site testing

95.55.−n Astronomical and space-research instrumentation *(see also 94.80.+g Instrumentation for aeronomy and magnetospheric studies; 07.87.+v Spaceborne and space research instruments, apparatus, and components)*

95.55.Aq Charge-coupled devices, image detectors, and IR detector arrays *(see also 85.60.Gz Photodetectors)*

95.55.Br Astrometric and interferometric instruments

95.55.Cs Ground-based ultraviolet, optical and infrared telescopes

95.55.Ev Solar instruments

95.55.Fw Space-based ultraviolet, optical, and infrared telescopes

95.55.Jz Radio telescopes and instrumentation; heterodyne receivers

95.55.Ka X- and γ-ray telescopes and instrumentation

95.55.Pe Lunar, planetary, and deep-space probes

95.55.Qf Photometric, polarimetric, and spectroscopic instrumentation *(see also 07.60.−j Optical instruments, equipment, and techniques)*

95.55.Rg Photoconductors and bolometers *(see also 07.57.Kp Bolometers, infrared submillimeter wave, microwave, and radiowave receivers and detectors in instruments)*

95.55.Sh Auxiliary and recording instruments; clocks and frequency standards

95.55.Vj Neutrino, muon, pion, and other elementary particle detectors; cosmic ray detectors *(see also 29.40.−n Radiation detectors-in nuclear physics)*

95.55.Ym Gravitational radiation detectors; mass spectrometers; and other instrumentation and techniques *(see also 04.80.−y Experimental studies of gravity in general relativity and gravitation)*

95.75.−z Observation and data reduction techniques; computer modeling and simulation

95.75.De Photography and photometry (including microlensing techniques)

95.75.Fg Spectroscopy and spectrophotometry

95.75.Hi Polarimetry

95.75.Kk Interferometry

95.75.Mn Image processing (including source extraction)

95.75.Pq Mathematical procedures and computer techniques

95.75.Qr Adaptive and segmented optics *(see also 42.68.Wt Remote sensing; LIDAR and adaptive systems in atmospheric optics)*

95.75.Rs Remote observing techniques

95.75.Tv Digitization techniques *(see also 07.05.Pj Image processing in instruments)*

95.75.Wx Time series analysis, time variability

95.80.+p Astronomical catalogs, atlases, sky surveys, databases, retrieval systems, archives, etc.

95.85.−e Astronomical observations (additional primary heading(s) must be chosen with these entries to represent the astronomical objects and/or properties studied)

95.85.Bh Radio, microwave (>1 mm)

95.85.Fm Submillimeter (300 m–1 mm)

95.85.Gn Far infrared (10–300 m)

95.85.Hp Infrared (3–10 m)

95.85.Jq Near infrared (0.75–3 m)

95.85.Kr Visible (390–750 nm)

95.85.Ls Near ultraviolet (300–390 nm)

95.85.Mt Ultraviolet (10–300 nm)

95.85.Nv X-ray

95.85.Pw γ-ray

95.85.Ry Neutrino, muon, pion, and other elementary particles; cosmic rays

95.85.Sz Gravitational radiation, magnetic fields, and other observations

95.90.+v Historical astronomy and archaeoastronomy; and other topics in fundamental astronomy and astrophysics; instrumentation, techniques, and astronomical observations

96. Solar System *(for the Earth, see sections 91–94)*

96.10.+i General, solar nebula, and cosmogony

96.20.−n Moon

96.20.Br Origin, formation, and age

96.20.Dt Features, landmarks, mineralogy, petrology, and atmosphere

96.20.Jz Gravitational field, selenodesy, magnetic fields

96.20.Ka Cratering

96.30.−t Planets, their satellites and rings; asteroids *(for comets, see 96.50.Gn)*

96.30.Dz Mercury

96.30.Ea Venus

96.30.Gc Mars

96.30.Kf Jupiter

96.30.Mh Saturn

96.30.Pj Uranus

96.30.Rm Neptune

96.30.Sn Pluto

96.30.Wr Planetary rings

96.30.Ys Asteroids (minor planets)

96.35.−j Planetary, asteroid, cometary, and satellite characteristics and properties *(see also 97.82.−j for extrasolar planetary systems)*

96.35.Cp Origin, formation, evolution, and ages

96.35.Er Chemical composition

96.35.Fs Mass, size; gravitational fields; rotation; orbits

96.35.Gt Surface features, cratering, and topography

96.35.Hv Neutral atmospheres

96.35.Kx Ionospheres; magnetospheres

96.35.Mz Interiors

96.35.Na Volcanism and tectonics

96.35.Pb Electric and magnetic fields

96.35.Se Interplanetary comparisons

96.40.−z Cosmic rays *(for cosmic rays outside the Solar System, see 98.70.Sa; for cosmic-ray interactions, see 13.85.Tp in hadron-induced high- and super high-energy interactions)*

96.40.Cd Interplanetary propagation and effects

96.40.De Composition, energy spectra, and interactions

96.40.Fg Energetic solar particles and photons

96.40.Kk Solar modulation and geophysical effects

96.40.Pq Extensive air showers

96.40.Tv Neutrinos and muons

96.40.Vw Cosmic-ray effects in meteorites and terrestrial matter

96.50.−e Interplanetary space *(for asteroids, see 96.30.Ys)*

96.50.Bh Solar and interplanetary electric and magnetic fields (including solar wind fields)

96.50.Ci Solar wind plasma

96.50.Dj Interplanetary gas and dust (including gegenschein and zodiacal light)

96.50.Ek Solar wind interactions with planets, satellites, and comets (for interactions with Earth, see 94.30.Va)

96.50.Fm Shock waves

96.50.Gn Comets

96.50.Hp Oort cloud

96.50.Jq Kuiper belt

96.50.Kr Meteors, meteoroids, and meteor streams

96.50.Mt Meteorites, micrometeorites, and tektites

96.50.Pw Particle acceleration

96.50.Qx Stream-stream interactions

96.50.Ry Waves and discontinuities

96.60.−j Solar physics

96.60.Bn Diameter, figure, rotation, mass

96.60.Fs Chemical composition

96.60.Hv Electric and magnetic fields

96.60.Jw Solar interior *(for solar neutrinos, see 26.65.+t in nuclear astrophysics)*

96.60.Ly Oscillations and waves; helioseismology

96.60.Mz Photosphere, granulation

96.60.Na Chromosphere and chromosphere−corona transition; spicules

96.60.Pb Corona; coronal loops, streamers, and holes

96.60.Qc Sunspots, faculae, plages

96.60.Rd Flares, bursts, and related phenomena

96.60.Se Prominences

96.60.Tf Solar electromagnetic radiation *(see also 92.60.Vb Solar radiation in meteorology)*

96.60.Vg Particle radiation, solar wind, and solar neutrinos *(see also 96.50.Ci Solar wind plasma and 96.50.Ek Solar wind interactions with planets, satellites, and comets; see also 26.65.+t Solar neutrinos in nuclear astrophysics)*

96.60.Wh Coronal mass ejection

96.90.+c Other topics on the solar system (restricted to new topics in section 96)

97. Stars

97.10.−q Stellar characteristics and properties *(see also 04.40.Dg Relativistic stars in general relativity and gravitation and section 26 Nuclear astrophysics)*

97.10.Bt Star formation

97.10.Cv Stellar structure, interiors, evolution, nucleosynthesis, ages

97.10.Ex Stellar atmospheres (photospheres, chromospheres, coronae, magnetospheres); radiative transfer; opacity and line formation

97.10.Fy Circumstellar shells, clouds, and expanding envelopes; circumstellar masers *(for interstellar masers, see 98.38.Er or 98.58.Ec)*

97.10.Gz Accretion and accretion disks

97.10.Jb Stellar activity

97.10.Kc Stellar rotation

97.10.Ld Magnetic and electric fields; polarization of starlight

97.10.Me Mass loss and stellar winds

97.10.Nf Masses

97.10.Pg Radii

97.10.Qh Surface features (including starspots)

97.10.Ri Luminosities; magnitudes; effective temperatures, colors, and spectral classification

97.10.Sj Pulsations, oscillations, and stellar seismology

97.10.Tk Abundances, chemical composition

97.10.Vm Distances, parallaxes

97.10.Wn Proper motions and radial velocities (line-of-sight velocities); space motions *(see also 95.10.Jk Astrometry and reference systems)*

97.10.Xq Luminosity and mass functions

97.10.Yp Star counts, distribution, and statistics

97.10.Zr Hertzsprung-Russell, color-magnitude, and color-color diagrams

97.20.−w Normal stars (by class): general or individual

97.20.Ec Main-sequence: early-type stars (O and B)

97.20.Ge Main-sequence: intermediate-type stars (A and F)

97.20.Jg Main-sequence: late-type stars (G, K, and M)

97.20.Li Giant and subgiant stars

97.20.Pm Supergiant stars

97.20.Rp Faint blue stars (including blue stragglers), white dwarfs, degenerate stars, nuclei of planetary nebulae *(for planetary nebulae, see 98.38.Ly or 98.58.Li)*

97.20.Tr Population II stars (horizontal branch, metal poor, etc.)

97.20.Vs Low luminosity stars, subdwarfs, and brown dwarfs

97.20.Wt Population III stars

97.21.+a Pre-main sequence objects, young stellar objects (YSO's) and protostars (T Tauri stars, Orion population, Herbig−Haro objects, Bok globules, bipolar outflows,

cometary nebulae, etc.) *(see also 98.38.Fs and 98.58.Fd Jets, outflows and bipolar flows in the Milky Way and external galaxies respectively)*

97.30.−b Variable and peculiar stars (including novae)

97.30.Dg Low-amplitude blue variables (alpha Cygni, beta Cephei, delta Scuti, delta Delphini, delta Canis Majoris, SX Phoenicius, etc.)

97.30.Eh Emission-line stars (Of, Be, Luminous Blue Variables, Wolf−Rayet, etc.)

97.30.Fi Chemically peculiar stars (Ap, Am, etc.)

97.30.Gj Cepheids (delta Cephei, W Virginis)

97.30.Hk Carbon stars, S stars, and related types (C, S, R, and N)

97.30.Jm Long-period variables (Miras) and semiregulars

97.30.Kn RR Lyrae stars; RV Tauri and PV Telescopii variables

97.30.Nr Flare stars (UV Ceti, RS Canum Venaticorum, FU Orionis, R Coronae Borealis variables, etc.)

97.30.Qt Novae, dwarf novae, recurrent novae,and other cataclysmic (eruptive) variables *(see also 97.80.Gm, Jp Cataclysmic binaries and X-ray binaries)*

97.30.Sw Unusual and peculiar variables

97.60.−s Late stages of stellar evolution (including black holes) *(see also 04.40.Dg Relativistic stars in general relativity and gravitation)*

97.60.Bw Supernovae *(see also 26.30.+k Nucleosynthesis in novae, supernovae and other explosive stars and 26.50.+x Nuclear physics aspects of supernovae evolution)*

97.60.Gb Pulsars

97.60.Jd Neutron stars *(see also 26.60.+c Nuclear matter aspects of neutron stars in nuclear physics)*

97.60.Lf Black holes *(see also 04.70.−s Physics of black holes in general relativity and gravitation; for galactic black holes, see 98.35.Jk and 98.62.Js)*

97.80.−d Binary and multiple stars

97.80.Af Astrometric and interferometric binaries

97.80.Di Visual binaries

97.80.Fk Spectroscopic binaries; close binaries

97.80.Gm Cataclysmic binaries (novae, dwarf novae, recurrent novae, and nova-like objects); symbiotic stars *(see also 97.30.Qt Novae)*

97.80.Hn Eclipsing binaries

97.80.Jp X-ray binaries *(see also 98.70.Qy X-ray sources and 97.60.Gb Pulsars)*

97.80.Kq Multiple stars

97.82.−j Extrasolar planetary systems

97.82.Cp Photometric and spectroscopic detection; coronographic detection; interferometric detection

97.82.Fs Substellar companions; planets

97.82.Jw Infrared excess; debris disks; protoplanetary disks; exo-zodiacal dust

97.90.+j Other topics on stars (restricted to new topics in section 97)

98. Stellar systems; interstellar medium; galactic and extragalactic objects and systems; the Universe

98.10.+z Stellar dynamics and kinematics

98.20.−d Stellar clusters and associations

98.20.Af Associations of stars (OB, T, R) in the Milky Way

98.20.Bg Associations of stars (OB, T, R) in external galaxies

98.20.Di Open clusters in the Milky Way

98.20.Fk Open clusters in external galaxies

98.20.Gm Globular clusters in the Milky Way

98.20.Jp Globular clusters in external galaxies

98.35.−a Characteristics and properties of the Milky Way galaxy

98.35.Ac Origin, formation, evolution, age, and star formation

98.35.Bd Chemical composition and chemical evolution

98.35.Ce Mass and mass distribution

98.35.Df Kinematics, dynamics, and rotation

98.35.Eg Electric and magnetic fields

98.35.Gi Galactic halo

98.35.Hj Spiral arms and galactic disk

98.35.Jk Galactic center, bar, circumnuclear matter, and bulge (including black hole and distance measurements) *(see also 04.70.−s Physics of black holes in general relativity and gravitation)*

98.35.Ln Stellar content and populations; morphology and overall structure

98.35.Mp Infall and accretion

98.35.Nq Galactic winds and fountains

98.35.Pr Solar neighborhood

98.38.−j Interstellar medium (ISM) and nebulae in Milky Way

98.38.Am Physical properties (abundances, electron density, magnetic fields, scintillation, scattering, kinematics, dynamics, turbulence, etc.)

98.38.Bn Atomic, molecular, and chemical, and grain processes

98.38.Cp Interstellar dust grains; diffuse emission; infrared cirrus

98.38.Dq Molecular clouds, H_2 clouds, dense clouds, and dark clouds

98.38.Er Interstellar masers *(for circumstellar masers, see 97.10.Fy)*

98.38.Fs Jets, outflows, and bipolar flows *(for pre-main sequence objects, see 97.21.+a)*

98.38.Gt H I regions and 21-cm lines; diffuse, translucent, and high-velocity clouds

98.38.Hv H II regions; emission and reflection nebulae

98.38.Jw Infrared emission

98.38.Kx Intercloud medium (ICM); hot and highly ionized gas; bubbles

98.38.Ly Planetary nebulae *(for nuclei of planetary nebulae, see also 97.20.Rp)*

98.38.Mz Supernova remnants

98.52.−b Normal galaxies; extragalactic objects and systems (by type)

98.52.Cf Classification and classification systems

98.52.Eh Elliptical galaxies

98.52.Lp Lenticular (S0) galaxies

98.52.Nr Spiral galaxies

98.52.Sw Irregular and morphologically peculiar galaxies

98.52.Wz Dwarf galaxies (elliptical, irregular, and spheroidal)

98.54.−h Quasars; active or peculiar galaxies, objects, and systems

98.54.Aj Quasars *(for quasar absorption and emission-line systems; Lyman forest, see 98.62.Ra)*

98.54.Cm Active and peculiar galaxies and related systems (including BL Lacertae objects, blazars, Seyfert galaxies, Markarian galaxies, and active galactic nuclei)

98.54.Ep Starburst galaxies and infrared excess galaxies

98.54.Gr Radio galaxies

98.54.Kt Protogalaxies; primordial galaxies

98.56.−p Local group; Magellanic Clouds

98.56.Ew Elliptical galaxies

98.56.Ne Spiral galaxies (M31 and M33)

98.56.Si Magellanic Clouds and other irregular galaxies

98.56.Tj Magellanic stream

98.56.Wm Dwarf galaxies (elliptical, irregular, and spheroidal)

98.58.−w Interstellar medium (ISM) and nebulae in external galaxies

98.58.Ay Physical properties (abundances, electron density, magnetic fields, scintillation, scattering, kinematics, dynamics, turbulence, etc.)

98.58.Bz Atomic, molecular, chemical, and grain processes

98.58.Ca Interstellar dust grains; diffuse emission; infrared cirrus

98.58.Db Molecular clouds, H_2 clouds, dense clouds, and dark clouds

98.58.Ec Interstellar masers *(for circumstellar masers, see 97.10.Fy)*

98.58.Fd Jets, outflows and bipolar flows *(for pre-main sequence objects, see 97.21.+a)*

98.58.Ge H I regions and 21-cm lines; diffuse, translucent, and high-velocity clouds

98.58.Hf H II regions; emission and reflection nebulae

98.58.Jg Infrared emission

98.58.Kh Intercloud medium (ICM); hot and highly ionized gas; bubbles

98.58.Li Planetary nebulae *(for nuclei of planetary nebulae, see also 97.20.Rp)*

98.58.Mj Supernova remnants

98.58.Nk Tidal tails; H I shells

98.62.−g Characteristics and properties of external galaxies and extragalactic objects *(for the Milky Way, see 98.35.−a)*

98.62.Ai Origin, formation, evolution, age, and star formation

98.62.Bj Chemical composition and chemical evolution

98.62.Ck Masses and mass distribution

98.62.Dm Kinematics, dynamics, and rotation

98.62.En Electric and magnetic fields

98.62.Gq Galactic halos

98.62.Hr Spiral arms and bars; galactic disks

98.62.Js Galactic nuclei (including black holes), circumnuclear matter, and bulges *(see also 04.70.−s Physics of black holes in general relativity and gravitation)*

98.62.Lv Stellar content and populations; radii; morphology and overall structure

98.62.Mw Infall, accretion, and accretion disks *(see also 04.70.−s Physics of black holes in general relativity and gravitation)*

98.62.Nx Jets and bursts; galactic winds and fountains

98.62.Py Distances, redshifts, radial velocities; spatial distribution of galaxies *(see also 98.80.Es Observational cosmology)*

98.62.Qz Magnitudes and colors; luminosities

98.62.Ra Intergalactic matter; quasar absorption and emission-line systems; Lyman forest *(for quasars, see 98.54.Aj; for intracluster matter see 98.65.Hb)*

98.62.Sb Gravitational lenses and luminous arcs *(see also 95.30.Sf Relativity and gravitation in fundamental aspects of astrophysics and section 04 General relativity and gravitation)*

98.62.Tc Astrometry; identification

98.62.Ve Statistical and correlative studies of properties (luminosity and mass functions; mass-to-light ratio; Tully-Fisher relation, etc.)

98.65.−r Galaxy groups, clusters, and superclusters; large scale structure of the Universe

98.65.At Interacting galaxies; galaxy pairs, and triples

98.65.Bv Small and compact galaxy groups

98.65.Cw Galaxy clusters

98.65.Dx Superclusters; large-scale structure of the Universe (including voids, pancakes, great wall, etc.)

98.65.Fz Galaxy mergers, collisions, and tidal interactions

98.65.Hb Intracluster matter; cooling flows

98.70.−f Unidentified sources of radiation outside the Solar System

98.70.Dk Radio sources

. . . . *Quasars, see 98.54.Aj*

98.70.Lt IR sources *(for IR sources in interstellar medium, see 98.38.Jw and/or 98.58.Jg)*

98.70.Qy X-ray sources; X-ray bursts *(see also 97.30.Qt Novae, dwarf novae, 97.80.Jp X-ray binaries)*

98.70.Rz γ-ray sources; γ-ray bursts

98.70.Sa Cosmic rays (including sources, origin, acceleration, and interactions) *(see also 26.40.+r Cosmic ray nucleosynthesis in nuclear astrophysics)*

98.70.Vc Background radiations

98.80.−k Cosmology *(see also section 04 General relativity and gravitation; for origin and evolution of galaxies, see 98.62.Ai; for elementary particle and nuclear processes, see 95.30.Cq; for dark matter, see 95.35.+d; for superclusters and large-scale structure of the Universe, see 98.65.Dx)*

98.80.Bp Origin and formation of the Universe

98.80.Cq Particle-theory and field-theory models of the early Universe (including cosmic pancakes, cosmic strings, chaotic phenomena, inflationary universe, etc.) *(see also 11.25.−w Strings and branes, and 11.10.−z in general theory of fields and particles)*

98.80.Es Observational cosmology (including Hubble constant, distance scale, cosmological constant, early Universe, etc)

98.80.Ft Origin, formation, and abundances of the elements *(see also 26.35.+c Big Bang nucleosynthesis in nuclear astrophysics)*

98.80.Jk Mathematical and relativistic aspects of cosmology

98.80.Qc Quantum cosmology *(see also 04.60.−m Quantum gravity in general relativity and gravitation)*

98.90.+s Other topics on stellar systems; interstellar medium; galactic and extragalactic objects and systems; the Universe (restricted to new topics in section 98)

99.10.−x Errata and other corrections

99.10.Cd Errata

99.10.Fg Publisher's note

99.10.Jk Corrected article
